# Protein-based pan-RAS inhibitor induces tumor regression in female mice via IFNγ and CD8^+^ T cell-dependent tumor necrosis

**DOI:** 10.1038/s41467-026-73300-z

**Published:** 2026-05-16

**Authors:** Teiko Komori Nomura, Kazuki Heishima, Hidefumi Mukai, Kosuke Arai, Abdelazim Elsayed Elhelaly, Hirobumi Fuchigami, Shota Warashina, Tsuyoshi Tahara, Fuminori Hyodo, Masayuki Matsuo, Masahiro Yasunaga, Kazunori Aoki, Ryo Honda

**Affiliations:** 1 United Graduate School of Drug Discovery and Medical Information Sciences, Gifu University, Gifu, Japan; 2 Center for One Medicine Innovative Translational Research (COMIT), Gifu University, Gifu, Japan; 3 Institute for Advanced Study, Gifu University, Gifu, Japan; 4 Department of Pharmaceutical Informatics, Graduate School of Biomedical Sciences, Nagasaki University, Nagasaki, Japan; 5 Department of Immune Medicine, National Cancer Center Research Institute, Tokyo, Japan; 6 Department of Radiology, Gifu University, Gifu, Japan; 7 Department of Frontier Science for Imaging, School of Medicine, Gifu University, Gifu, Japan; 8 Department of Food Hygiene and Control, Faculty of Veterinary Medicine, Suez Canal University, Ismailia, Egypt; 9 Division of Developmental Therapeutics, Exploratory Oncology Research and Clinical Trial Center, National Cancer Center, Kashiwa, Japan; 10 Department of In vivo Imaging, Advanced Research Promoting Center, Tokushima University, Tokushima, Japan; 11 Innovation Research Center for Quantum Medicine, Gifu University, Gifu, Japan; 12 Department of Pharmacology, School of Medicine, Gifu University, Gifu, Japan

**Keywords:** Targeted therapies, Recombinant protein therapy, Biochemistry, Biophysics

## Abstract

Mutations in K/H/N-RAS occur in approximately 30% of human cancers, yet most RAS mutants remain undruggable in clinical settings. Here, we describe a protein-based pan-RAS inhibitor, RRSP-RBD, that combines a RAS/Rap1A-specific endopeptidase (RRSP) with a RAS-binding domain (RBD). This engineered fusion protein localizes to RAS on the plasma membrane, where it cleaves RAS, disrupts RAS-effector interactions, and effectively inhibits downstream RAS signaling. To achieve intracellular delivery of RRSP-RBD in vivo, we engineer two cell-permeable variants. The diphtheria toxin-based version (RRSP-RBD-DTB) demonstrates femtomolar anti-tumor potency and induces tumor regression in a xenograft mouse model. The cell-permeable peptide-based version (RRSP-RBD-TAT) exhibits robust anti-tumor activity in syngeneic models without inducing irreversible toxicity in normal tissues. Interestingly, anti-tumor efficacy of RRSP-RBD-TAT critically depends on the tumor microenvironment, requiring infiltration by IFNγ^+^ CD8^+^ T cells to mediate tumor regression. Pharmacokinetic and toxicity evaluations indicate that RRSP-RBD is tolerated under the conditions tested and support further investigation as a protein-based pan-RAS inhibitor (all mouse studies were performed in female mice).

## Abstract (web summary)

Many cancers depend on RAS signaling, but blocking diverse RAS mutants remains challenging. Here, authors engineer a protein-based pan-RAS inhibitor that induces tumor regression in mouse models and reveals an immune-dependent mechanism of action

## Introduction

Activating mutations in RAS (KRAS, HRAS, and NRAS) are implicated in 20–30% of all human cancers, including lung cancer, colorectal cancer, and pancreatic ductal adenocarcinoma^1^. These mutations, predominantly occurring in codons 12, 13, or 61, impair GTP hydrolysis or GDP/GTP exchange activity, shifting the cellular equilibrium toward RAS-GTP. This leads to the constitutive activation of the ERK and AKT pathways, driving uncontrolled proliferation and survival in cancer cells. Consequently, RAS has been a top therapeutic target for over four decades. Recent advancements in drug discovery have led to the approval of small-molecule inhibitors targeting the KRAS-G12C, as well as the development of KRAS-G12D inhibitors (e.g., ASP3082 and RMC-9805) that are currently undergoing clinical evaluation. However, these inhibitors often afford only short-lived clinical benefits due to acquired resistance^2^. Moreover, other RAS mutations, including G12V, G13X, and Q61X, remain difficult to target, presenting significant therapeutic challenges.

To address these limitations, the development of pan-RAS inhibitors that can simultaneously target multiple RAS mutants and wild-type RAS has emerged as a promising strategy. To date, RMC-6236, a small-molecule pan-RAS inhibitor currently in Phase II/III trials, has demonstrated encouraging clinical activity^3,4^. Other pan-RAS or pan-KRAS inhibitors, including ADT-1004 and BI-2865, have shown significant anti-tumor activity in preclinical models without toxicity^5–7^. Pan-RAS inhibitors offer distinct advantages over allele-specific inhibitors, including broader applicability across diverse RAS mutants and the ability to forestall acquired resistance by concurrently blocking compensatory activation of ERK and AKT signaling mediated by wild-type RAS or RAS harboring second-site mutations^1,8^. Despite these advantages, potential toxicity arising from targeting wild-type RAS in normal tissues remains a critical concern. Although RAS-mutant cancer cells rely heavily on RAS signaling for survival—indeed, a Phase I/Ib study of RMC-6236 reported tolerable adverse events—further preclinical studies with multiple pan-RAS inhibitors are needed to validate the therapeutic potential of the pan-RAS approach.

A major obstacle in developing direct RAS inhibitors is the limited availability of druggable binding pockets on the RAS protein surface. Small-molecule inhibitors typically target the switch-II pocket of RAS; however, this approach is vulnerable to resistance arising from secondary mutations within this region. Protein-based therapeutics provide an alternative by circumventing these structural constraints. Multiple RAS-binding proteins with low-nanomolar affinities, including naturally occurring RAS-binding domains (RBDs)^9^ and engineered scaffolds such as DARPins^10^, monobodies^11^, nanobodies^12^, and antibodies^13^, have been identified. These proteins inhibit RAS signaling independently of conventional binding sites, and in some instances have been further engineered for proteolysis-dependent degradation by fusion with E3 ligases^10,11^. Additionally, bacterial toxins have yielded unique enzymes capable of irreversibly inactivating RAS through post-translational modifications (PTMs). For example, Ras/Rap1A-specific endopeptidase (RRSP) cleaves RAS at residues Y32 and E33^14,15^; TpeL glycosylates RAS at residue T35^16^; and ExoS ADP-ribosylates RAS at residue R41^17^.

Despite their potential, the clinical application of protein-based RAS inhibitors has been limited primarily by their poor cell permeability. Strategies such as cell-permeable peptides (CPPs)^9,12,13^ and toxin-based delivery systems^16,18^ have been employed to improve cytosolic delivery, yet their efficacy remains modest, particularly in in vivo animal model. Indeed, we previously developed a protein-based pan-RAS inhibitor by fusing a high affinity RBD (cRaf-v1, with dissociation constant of 34 nM to KRAS-GTP and 1.9 µM to KRAS-GDP) to a CPP^9^. Although this inhibitor demonstrated in vitro activity against multiple K/H/N-RAS mutant cell lines at micromolar concentrations, it failed to exhibit anti-tumor efficacy in a Colon-26 syngeneic mouse model. This result underscores the need to design more potent protein constructs that are able to surmount poor membrane permeability.

Here, we present a protein-based pan-RAS inhibitor that fuses RBDs with RAS-targeting PTM enzymes. This inhibitor exhibits robust anti-tumor activity in vivo and induces durable tumor regression as a monotherapy when delivered via toxin- or CPP-based systems. Furthermore, we show that this pan-RAS inhibitor not only promotes tumor regression but also synergizes with anti-tumor immunity, notably via interactions with IFNγ^+^ CD8^+^ T cells. Pharmacokinetic and toxicity evaluations further demonstrate a favorable safety profile, underscoring the therapeutic potential of RRSP-RBD as a protein-based pan-RAS inhibitor.

## Results

### Development of RRSP-RBD fusion protein

**Fig. 1:**
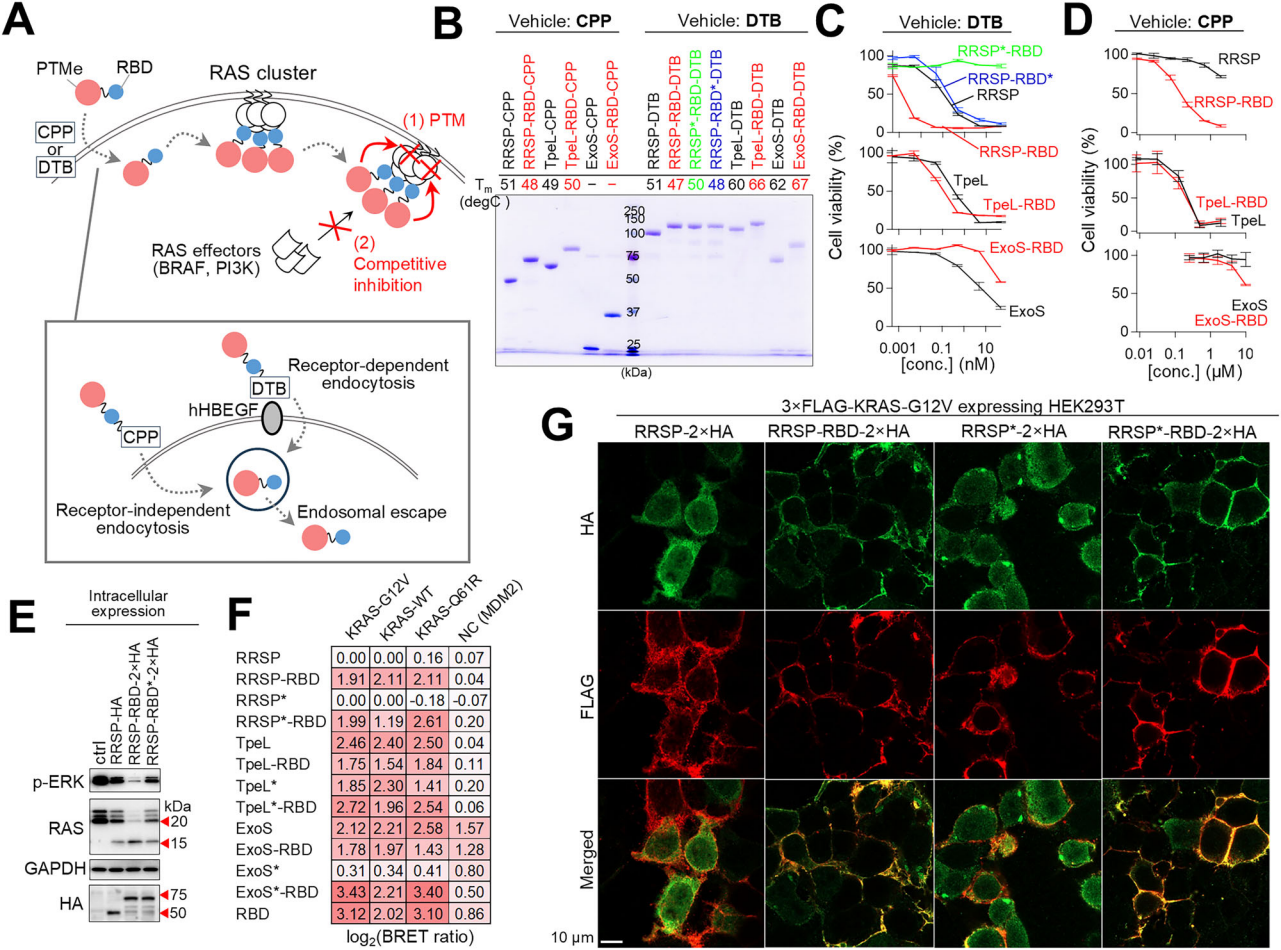
Design and characterization of PTMe-RBD fusion proteins. **A** Proposed mechanism of action and intracellular delivery of PTMe (RAS-targeting post-translational modification enzyme)-RBD (RAS-binding domain) fusion proteins. PTMe-RBD is delivered into the cytoplasm via endocytic pathways mediated by receptor-independent CPP (cell-penetrating peptides) or human HBEGF-dependent DTB (diphtheria toxin fragment B). Once in cytoplasm, PTMe-RBD accumulates at membrane-bound RAS, inactivating it via enzymatic cleavage and competitive inhibition. **B** CBB-stained SDS-PAGE image and melting temperature (T_m_) of synthesized PTMe-RBD proteins. **C** In vitro activities of DTB-conjugated PTMe-RBDs in HCT-116 cells (human KRAS-G12D mutant). Asterisks indicate inactive mutants (RRSP*, H4030A; RBD*, R88A/H89A). Data are mean ± SD of *n* = 3 technical replicate wells. **D** In vitro activities of CPP-conjugated PTMe-RBDs in CT-26 cells (mouse KRAS-G12D mutant) using TAT(47–57) as the CPP. Data are mean ± SD of *n* = 3 technical replicate wells. **E** Western blot analysis of lysates from PANC-1 cells (human KRAS-G12D mutant) expressing 2×HA-tagged PTMe-RBD. Representative immunoblot from two independent experiments with similar results. **F** BRET ratios of HEK293T cells expressing Nanoluc-tagged KRAS-G12V, KRAS-WT, KRAS-Q61R, or MDM2 (negative control) with Halotag-fused PTMe-RBDs. Asterisks indicate catalytically inactive mutants (RRSP*, H4030A; TpeL*, D286A/D288A; ExoS*, E379A/E381A). **G** Immunofluorescence staining of HEK293T cells co-expressing 3×FLAG-KRAS-G12V and 2×HA-tagged PTMe-RBD. Representative images from two independent experiments with similar results. Additional fields of view are shown in Fig. S4.

In this study, we engineered fusion proteins composed of RBDs and RAS-targeting PTM enzymes (PTMe), including RRSP (aa. 3596–4080), TpeL (aa. 1–542), and ExoS (aa. 233–455). These fusion proteins were designed to inhibit RAS signaling through two complementary mechanisms (Fig. 1A): (1) the RBD component localizes and concentrates PTMe-RBD at RAS clusters on the plasma membrane, where the PTMe component can effectively inactivate RAS; and (2) the RBD competitively inhibits RAS-effector interactions, further suppressing RAS-driven signaling^9^.

To test this hypothesis, we synthesized 14 recombinant fusion proteins combining RBD and PTMe, each conjugated to either CPP or diphtheria toxin fragment B (aa. 180–535) (DTB) for intracellular delivery (Fig. 1B). CPP mediates receptor-independent endocytosis^19^, while DTB enables the transport of conjugated proteins into cells expressing human HBEGF^20^, a widely expressed antigen^21^. These fusion proteins were expressed in a soluble fraction using a standard *E. coli* expression system, and their stability was verified via thermal denaturation and circular dichroism assays (Fig. S1).

We evaluated their anti-tumor efficacy in RAS-mutant cell lines and identified RRSP-RBD-DTB as the most potent construct (Fig. 1C). RRSP-RBD-DTB, which integrates RRSP, RBD, and DTB, exhibited 100-fold greater potency than RRSP-DTB lacking the RBD component. By contrast, inactive variants of RRSP-RBD—RRSP*-RBD (H4030A mutation in RRSP, rendering it enzymatically inactive^15^) and RRSP-RBD* (R88A/H89A mutation in RBD, impairing its RAS-binding ability^9^)—did not display synergistic effects. Fusion proteins combining RBD with TpeL or ExoS showed minimal synergy. A similar trend was observed for CPP-based systems, although overall activity was six orders of magnitude lower than in the DTB-based system (Fig. 1D). Furthermore, intracellular expression of HA‑tagged RRSP‑RBD in PANC‑1 (KRAS‑G12D) confirmed robust on‑target activity: endogenous KRAS was cleaved and ERK phosphorylation (p‑ERK) was suppressed more effectively than with RRSP or RRSP‑RBD* (Fig. 1E). In complementary HEK293T co‑expression assays, RRSP‑RBD also cleaved exogenously expressed HRAS (WT and G12V), KRAS‑G12V, and NRAS‑G12V (Fig. S2). These observations highlight that the fusion of RBD with PTMe, particularly RRSP, confers a synergistic inhibition of H/K/N-RAS signaling.

To evaluate the RAS binding by PTMe-RBD constructs, we expressed NanoLuc-tagged KRAS and Halotag-labeled PTMe-RBD in HEK293T cells and then measured bioluminescence resonance energy transfer (BRET) (Figs. 1F and S3). Both RRSP and RRSP* were intrinsically weak RAS-binders; however, fusing them with RBD compensated for this low affinity, converting them into strong RAS-binders. Consistent with the binding properties of RBD^9^, RRSP-RBD bound KRAS-WT, G12V, and Q61R, underscoring its potential as a pan-RAS inhibitor. In contrast, TpeL and ExoS were already RAS-binders, and fusing them with RBD conferred no additional advantage.

We further investigated the subcellular localization of RRSP-RBD in HEK293T cells co-expressing HA-tagged RRSP-RBD and FLAG-tagged KRAS-G12V by immunofluorescence (Figs. 1G and S4). In line with the BRET data, RRSP-RBD and RRSP*-RBD, but not RRSP or RRSP*, colocalized with RAS on the plasma membrane. These findings demonstrate that adding an RBD to RRSP compensates for its weak binding affinity, thereby substantially enhancing its inhibitory effect on RAS signaling.

### RRSP-RBD-DTB is a femtomolar pan-RAS inhibitor that induces tumor regression in vivo

**Fig. 2:**
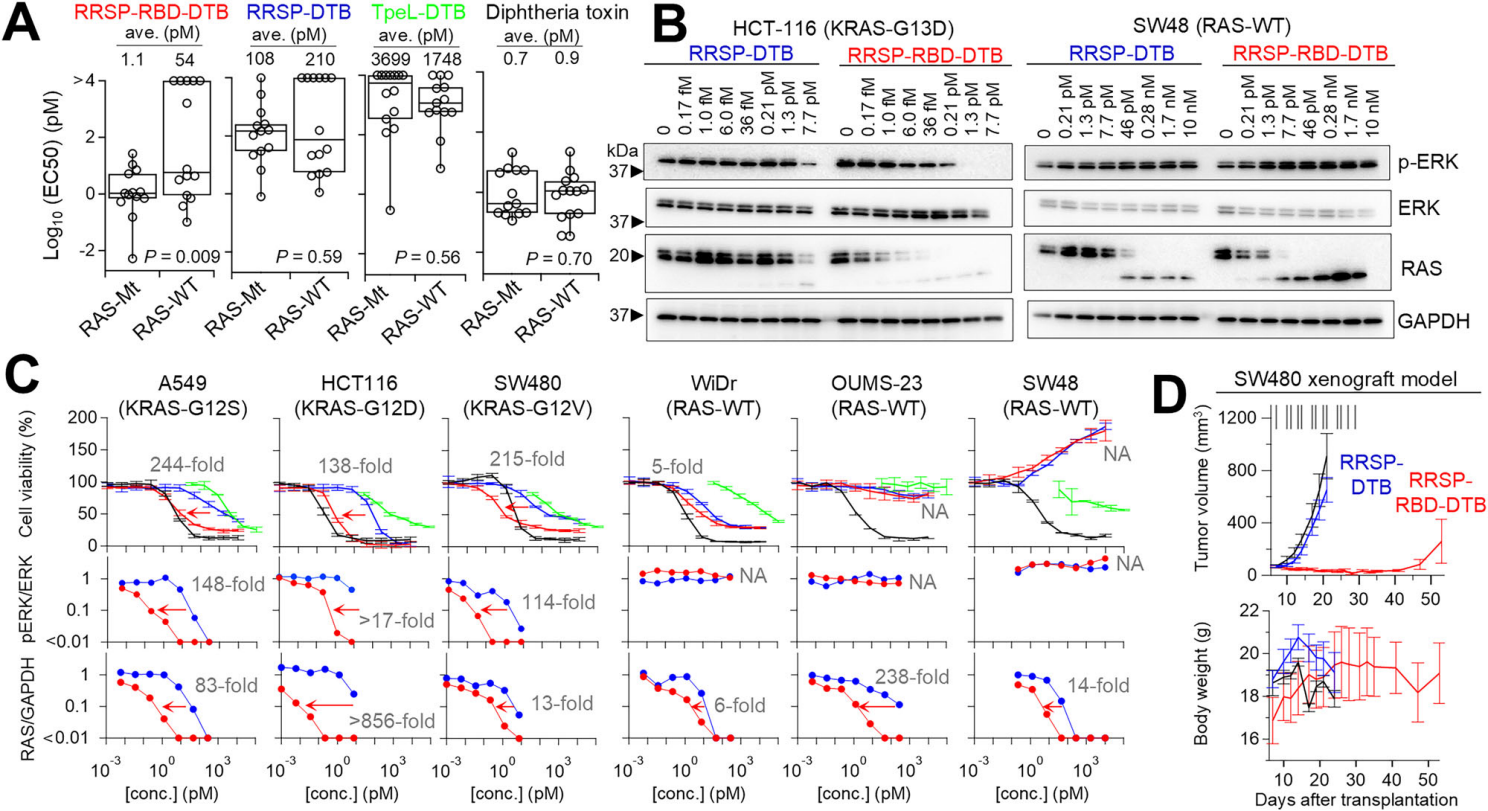
RRSP-RBD-DTB as a femtomolar Pan-RAS inhibitor. **A** EC50 values of diphtheria toxin-based proteins against 27 cultured cancer cell lines, including 13 RAS-mutant and 14 RAS-wild-type human cell lines. Box plots show the median (center line), 25th and 75th percentiles (box limits), and minimum and maximum values (whiskers). *P*-values were determined by an unpaired two-tailed t-test. **B** Western blot analysis of cell lysates collected 6 h after fusion proteins administration. **C** In vitro activities of fusion proteins in six cell lines. Cell viability was assessed by WST-8 assays, whereas the p-ERK/total ERK and RAS/GAPDH ratios were determined by Western blotting. Numbers within the figure denote the differences in activity between RRSP-RBD-DTB and RRSP-DTB. **D** Tumor volume and body weight changes in an SW480 xenograft model treated intravenously with fusion proteins at 10 mg/kg/day for 13 doses. Vertical lines indicate dosing days. Data represent mean ± SE (*n* = 5 mice per group).

We assessed the anti-tumor efficacy of RRSP-RBD-DTB and three other diphtheria toxin-based constructs (RRSP-DTB, TpeL-DTB, and full-length diphtheria toxin) across 27 human cancer cell lines (Fig. 2A). Among these 27 cell lines, 13 cancer cell lines harbor various types of RAS mutations (e.g., G12 C, G12V, G12D) (Supplementary Data 1). Notably, RRSP-RBD-DTB demonstrated a 50-fold selectivity toward RAS-mutated cancers versus RAS-wild type cell lines. Similar to other pan-RAS inhibitors^4,5^, some RAS-wild type cancers also showed vulnerability to RRSP-RBD-DTB, implying the presence of RAS addiction driven by mechanisms independent of RAS mutations. In contrast, the other constructs did not show selectivity toward RAS-mutated cancers. The average EC50 value for RAS-mutated cancers was 1.1 pM, comparable to that of full-length diphtheria toxin.

Immunoblot analysis of cell lysates collected 6 h after RRSP-RBD-DTB treatment demonstrated that the inhibitor cleaves RAS and inhibits p-ERK at concentrations of only a few femtomolar in RAS-mutated cancers (Figs. 2B, C and S5A, B). In RAS wild-type cancers, although RRSP-RBD-DTB cleaved RAS, the effect on p-ERK inhibition was less pronounced, likely reflecting their low dependency on RAS for ERK signaling. When compared to RRSP-DTB, the RAS-cleaving and p-ERK inhibitory activities of RRSP-RBD-DTB were enhanced by up to 868-fold and 148-fold, respectively, depending on the cell line.

In a subcutaneous SW480 transplanted xenograft model harboring the KRAS-G12V mutation, intravenous administration of RRSP-RBD-DTB (10 mg/kg/day, 13 doses) produced significant tumor regression without evident toxicity (Fig. 2D). Moreover, a single intravenous dose of RRSP‑RBD‑DTB (10 mg/kg) induced detectable RAS cleavage in tumor tissue 3 h after administration (Fig. S5C). In contrast, treatment with RRSP-DTB did not exhibit anti-tumor effects. Collectively, these data demonstrated that coupling RBD to RRSP significantly improves RAS inhibition both in vitro and in vivo.

### RRSP-RBD-TAT induced tumor regression in vivo but only in syngeneic models

Despite its potency, the DTB-based delivery system cannot enter mouse cells due to the E141H variant of HBEGF^22^ (see Supplementary Data 1 for micromolar EC50 values against mouse cancer cells). Consequently, in xenograft mouse models, this system exclusively targets transplanted human cancer cells, limiting toxicity and pharmacokinetic (PK) evaluations, as well as potential TME interactions, in mouse models. However, mouse models are ideal for examining RRSP-RBD because the amino acid sequences surrounding the RAS cleavage site and RBD binding site are perfectly conserved between humans and mice (Fig. S6). To overcome this limitation, we shifted our focus to a CPP-based isoform, RRSP-RBD-CPP.

**Fig. 3:**
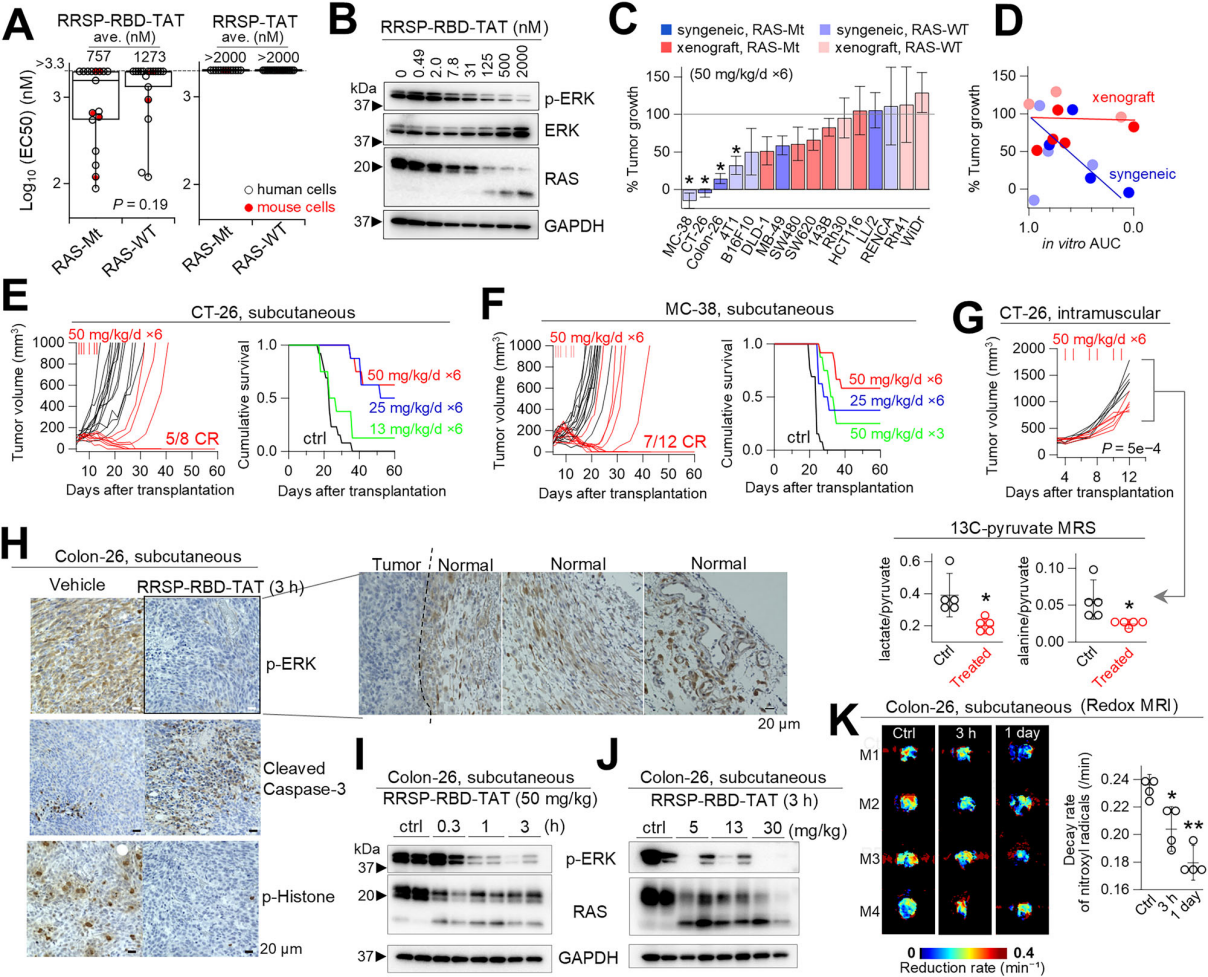
RRSP-RBD-TAT induces tumor regression exclusively in syngeneic models. **A** EC50 values of TAT(47–57)-based fusion proteins against 35 cultured cancer cell lines (27 human and 8 mouse). Box plots show the median (center line), 25th and 75th percentiles (box limits), and minimum and maximum values (whiskers). *P*-values were determined by an unpaired two-tailed t-test. **B** Western blot analysis of CT-26 cell lysates 24 h after RRSP-RBD-TAT administration. **C** Anti-tumor efficacy of 50 mg/kg/day RRSP-RBD-TAT for six doses in 16 tumor-bearing mouse models. Here, 100% denotes the tumor volume of the vehicle control, whereas percentages below 0% indicate that tumor has shrunk below its initial size at the start of treatment. Data are mean ± SE. Individual tumor-volume trajectories for each mouse in the corresponding models are shown in Supplementary Fig. S11. Each group included *n* = 6 mice for BALB/c and C57BL/6 models and *n* = 5 mice for BALB/c-nu/nu model. *P < 0.05, unpaired two-tailed t-test vs. control. Exact *P* values are provided in Supplementary Data 4. **D** Correlation between in vivo anti-tumor efficacy (% tumor growth from Fig. 3C) and in vitro anti-tumor efficacy (AUC, area under curve) from Supplementary Data 1. **E**, **F** Tumor volume changes and Kaplan-Meier survival curves in CT-26 and MC-38 syngeneic subcutaneous models treated with RRSP-RBD-TAT under various dosing protocols. The Kaplan-Meier endpoint was defined as the day tumor volume reached 1000 mm³. CR indicates the number of mice achieving complete tumor regression. **G** Tumor volume changes in the CT-26 syngeneic intramuscular model treated with RRSP-RBD-TAT (50 mg/kg/day, six doses). Tumor metabolism data were obtained using hyperpolarised ^13^C pyruvate magnetic resonance spectroscopy (MRS) 1–3 days after the final treatment. *P < 0.05, unpaired two-tailed t-test. **H** IHC staining of tumor and adjacent normal tissues 3 h after a single 50 mg/kg RRSP-RBD-TAT dose in the Colon-26 syngeneic subcutaneous model. Representative images from two independent experiments with similar results. Additional time-course images are shown in Supplementary Fig. S15. **I**, **J** Western blot analysis of tumor lysates collected 0.3–3 h after a single 5–50 mg/kg RRSP-RBD-TAT dose in the Colon-26 syngeneic subcutaneous model. Representative immunoblots from two independent experiments with similar results (total *N* = 4 tumors). **K** Redox metabolism of tumors analyzed by nitroxyl radical and in vivo DNP-MRI in the Colon-26 syngeneic subcutaneous model. Data are mean ± SD of *n* = 4 tumors. Vehicle vs. 3 h, *P* = 0.020; vehicle vs. 24 h, *P* = 0.006 (two-sided unpaired t-test).

To obtain more potent derivatives, we screened 29 RRSP-RBD-CPP constructs with various RBD/CPP combinations. In line with our previous work^9^, cRaf-v1 emerged as the most effective RBD, and TAT(47–57), polyarginines (R8 and R10), glycosaminoglycan-binding enhanced transduction (GET), and CPP2 were among the most potent CPPs (Fig. S7 and Supplementary Data 2). Of these, TAT(47–57) was chosen for its clinical availability^19^. We evaluated RRSP-RBD-TAT against 27 human and 8 mouse cancer cells (Fig. 3A and Supplementary Data 1). RRSP-RBD-TAT targeted both human and mice cancer cells with similar efficacy, whereas RRSP-TAT lacking the RBD component showed no activity. Unlike RRSP-RBD-DTB, RRSP-RBD-TAT did not exhibit a statistically significant preference for RAS-mutated cell lines, in part because in 20 of 35 cell lines, the EC50 exceeded the solubility limit of the protein (2 µM) and therefore could not be determined. Nevertheless, in responsive cells including CT-26 and 143B, RRSP-RBD-TAT induced RAS cleavage and p-ERK inhibition at as concentrations as low as 31–125 nM (Figs. 3B and S8). Additionally, RNA microarray analysis showed that 250 nM RRSP-RBD-TAT triggered gene expression changes consistent with KRAS inhibition (Fig. S9)^9^. These findings confirm that RRSP-RBD-TAT functions as a pan-RAS inhibitor, similar to RRSP-RBD-DTB, yet with broader applicability in mouse models.

We tested the in vivo efficacy of RRSP-RBD-TAT in eight subcutaneous xenograft models (immunodeficient athymic nude BALB/C mice transplanted with human cancers) and eight syngeneic models (immunocompetent BALB/C or C57BL/6 mice transplanted with mouse cancers). Endotoxin-free RRSP-RBD-TAT (≤ 13 EU/mg of protein, Fig. S10) was intravenously administered at 50 mg/kg/day for 6 days. In pilot studies, more aggressive treatment regimens resulted in transient weight loss or poor coat condition. Strikingly, RRSP-RBD-TAT induced significant tumor regression exclusively in syngeneic models (Figs. 3C and S11). In these syngeneic models, the in vivo anti-tumor activity roughly correlated with in vitro efficacy (Fig. 3D), whereas xenograft models showed no response—even in cell lines highly sensitive in vitro, such as 143B.

Among the syngeneic models, CT-26 responded most strongly, followed by MC-38 and Colon-26 (Figs. 3E, F and S12A). More than half of the mice in the CT-26 model achieved complete tumor regression after treatment cessation, even at a reduced dose (25 mg/kg/day), and the cured mice remained tumor‑free for over one year without adverse events. Furthermore, a delayed‑start regimen—initiating treatment at a mean tumor volume of 120 mm^3^ instead of 60 mm^3^—produced similar tumor‑regression effects (Fig. S12B). Additionally, RRSP-RBD-TAT suppressed tumor growth in a highly aggressive intramuscular CT-26 syngeneic model, accompanied by decreased lactate/pyruvate and alanine/pyruvate ratios (Figs. 3G and S13). Together, these findings underscore the robust anti‑tumor activity of RRSP‑RBD‑TAT in syngeneic models.

Histopathological examination of the Colon-26 tumors in a syngeneic model revealed that RRSP-RBD-TAT induced extensive tumor necrosis within 3–24 h post-treatment (Fig.　S14). By 3 h, RRSP-RBD-TAT had inhibited p-ERK and histone H3 phosphorylation and induced Caspase-3 cleavage (Figs. 3H and S15). Notably, the adjacent normal tissues did not display p-ERK inhibition, demonstrating the tumor-specific efficacy of RRSP-RBD-TAT. Immunoblot analysis of Colon-26 tumor lysates demonstrated that RRSP-RBD-TAT triggered RAS cleavage and p-ERK inhibition by 0.3 and 1 h, respectively (Fig. 3I), and these effects were detected at doses as low as 5 mg/kg (Fig. 3J). Furthermore, in vivo redox MRI showed slow reduction of nitroxyl radicals in tumors at 3 h after post-treatment (Fig. 3K), indicating altered redox homeostasis in cancer cells^23^. Collectively, these data demonstrate that RRSP-RBD-TAT cleaves RAS and inhibits p-ERK within 1 h, ultimately driving tumor-specific necrosis within 24 h.

### RRSP-RBD-TAT induced IFNγ- and CD8^+^ T cell-dependent tumor necrosis

**Fig. 4:**
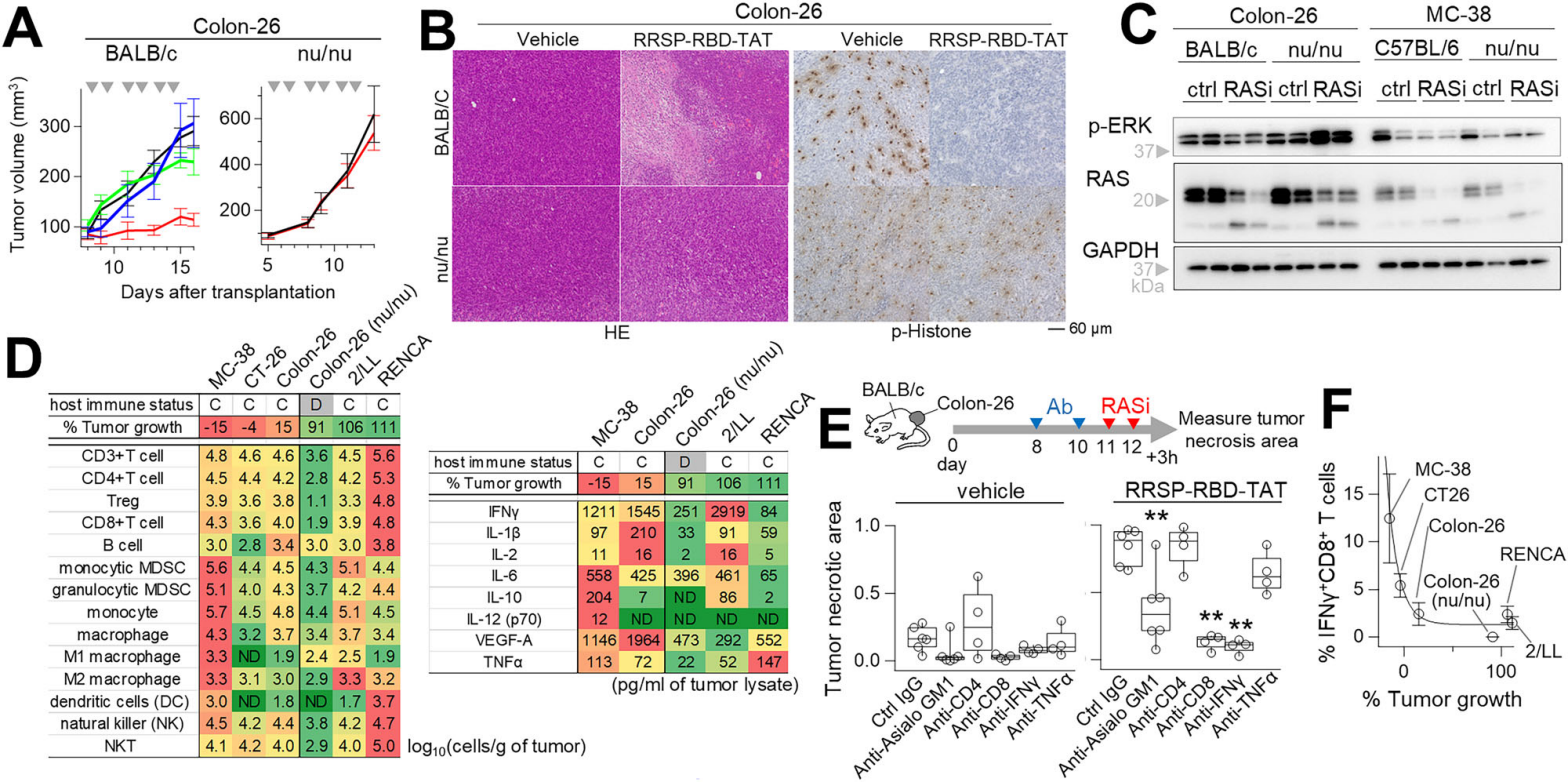
Tumor necrosis via IFNγ and CD8^+^ T cells in RRSP-RBD-TAT-treated mice. **A** Tumor volume changes in Colon-26 transplanted into immunocompetent BALB/C mice and immunodeficient nu/nu mice treated with 50 mg/kg/day RRSP-RBD-TAT (red), inactive mutants (RRSP*-RBD-TAT, blue; RRSP-RBD*-TAT, green), or vehicle control (black) for six doses. Data represent mean ± SE (*n* = 5 mice per group). **B** H&E and IHC staining of tumor tissues obtained 3 h after a single 50 mg/kg RRSP-RBD-TAT dose. Representative H&E and IHC images from one experiment; similar findings were observed across the analyzed mice in the cohort. **C** Western blot analysis of tumor lysates 3 h after a single 50 mg/kg RRSP-RBD-TAT (RASi) dose in CT-26 or MC-38 transplanted into immunocompetent (BALB/C or C57BL/6) or immunodeficient mice (nu/nu). **D** Quantification of tumor-infiltrating lymphocytes and cytokine concentrations in five immunocompetent (denoted by “C”) and one immunodeficient (denoted by “D”) model. **E** Tumor necrotic areas in the Colon-26 syngeneic model depleted of various immune factors using specific antibodies. Tumor tissues were excised 3 h after two doses of 50 mg/kg/day RASi (RRSP-RBD-TAT). Box plots show the median (center line), 25th and 75th percentiles (box limits), and minimum and maximum values (whiskers) (*n* = 4 or 6 mice per group). *P* values were calculated by one-way ANOVA followed by Tukey’s multiple-comparisons test (*P < 0.05, **P < 0.005). Exact *P* values are provided in Supplementary Data 4. **F** Correlation between the proportion of IFNγ^+^ CD8^+^ T cells and in vivo anti-tumor efficacy (% tumor growth from Fig. 3C) in the six models. Data are mean ± SD of *n* = 4 tumors.

We next investigated whether a host factor specific to syngeneic models underlies RRSP‑RBD‑TAT’s anti‑tumor activity. To this end, Colon‑26 and MC‑38 cells were transplanted into immunodeficient athymic nu/nu mice instead of the immunocompetent hosts used for the syngeneic studies. Notably, RRSP‑RBD‑TAT did not inhibit tumor growth in Colon‑26 or MC‑38 nu/nu mice (Figs. 4A and S16), nor did it induce tumor necrosis (Fig. 4B). By contrast, immunoblotting confirmed robust intratumoral target engagement—RAS cleavage—in both contexts, whereas in Colon‑26 nu/nu tumors p‑ERK showed a tendency to increase (Figs. 4C and S17A). Consistently, we observed clear RAS cleavage across non‑responding syngeneic models (RENCA and LL/2) and human xenografts (SW480, HCT‑116, MiaPaCa‑2), but no consistent p‑ERK inhibition (Fig. S17B, C). Together, these data indicate that target engagement (RAS cleavage) is model‑independent, but translation to durable p‑ERK suppression and tumor necrosis is model‑dependent. Furthermore, inactive variants (RRSP*‑RBD‑TAT and RRSP‑RBD*‑TAT) did not inhibit tumor growth even in the immunocompetent setting, indicating that effective on‑target RAS inhibition is required for tumor necrosis. These findings suggest that a host factor unique to immunocompetent mice—likely T‑cell immunity lacking in athymic nu/nu mice—is critical for triggering tumor necrosis following RAS cleavage.

To identify key immune factors, we measured tumor-infiltrating lymphocytes (TILs) and cytokines in the immunocompetent and immunodeficient Colon-26 models, as well as in four syngeneic models exhibiting various drug sensitivity (highly sensitive: MC-38 and CT-26; insensitive: 2/LL and RENCA) (Fig. 4D). We performed this analysis pre-treatment because RRSP-RBD-TAT significantly reduced TIL populations in Colon-26 tumors 3–24 h post-treatment (Fig. S18), likely due to extensive tumor necrosis. As a result, the immunodeficient Colon-26 model exhibited markedly lower baseline levels of CD3^+^, CD4^+^, Treg, CD8^+^ T cells in tumors (< 10-fold) and a moderately reduced levels of natural killer (NK) cells, IFNγ, IL-2, TNFα, and VEGF-A (< 3-fold) relative to its immunocompetent counterpart.

To further refine the key immune factors, we performed in vivo depletion experiments using specific antibodies. Remarkably, the removal of CD8^+^ T cells or IFNγ in immunocompetent Colon-26 and MC-38 mice completely abrogated RRSP-RBD-TAT-induced tumor necrosis (Figs. 4E, S19 and S20), implicating IFNγ^+^ and CD8^+^ T cells as essential mediators. Depletion of NK cells/monocytes using an anti-Asialo-GM1 antibody partially mitigated tumor necrosis, although the precise significance of this observation remains unclear; it may indicate that removal of these cells indirectly diminishes IFNγ and CD8^+^ T cell responses. Indeed, the population of IFNγ^+^ CD8^+^ T cell correlated strongly with drug efficacy across the six models (Fig. 4F), while NK cells, monocytes, bulk CD8^+^ T cells, bulk IFNγ, and the other TILs/cytokines did not (Fig. 4D). Thus, the pre-treatment TME—and particularly IFNγ^+^ CD8^+^ T cells—emerges as a critical determinant for RRSP-RBD-TAT-driven tumor necrosis.

### PK and safety analysis of RRSP-RBD-TAT

**Fig. 5:**
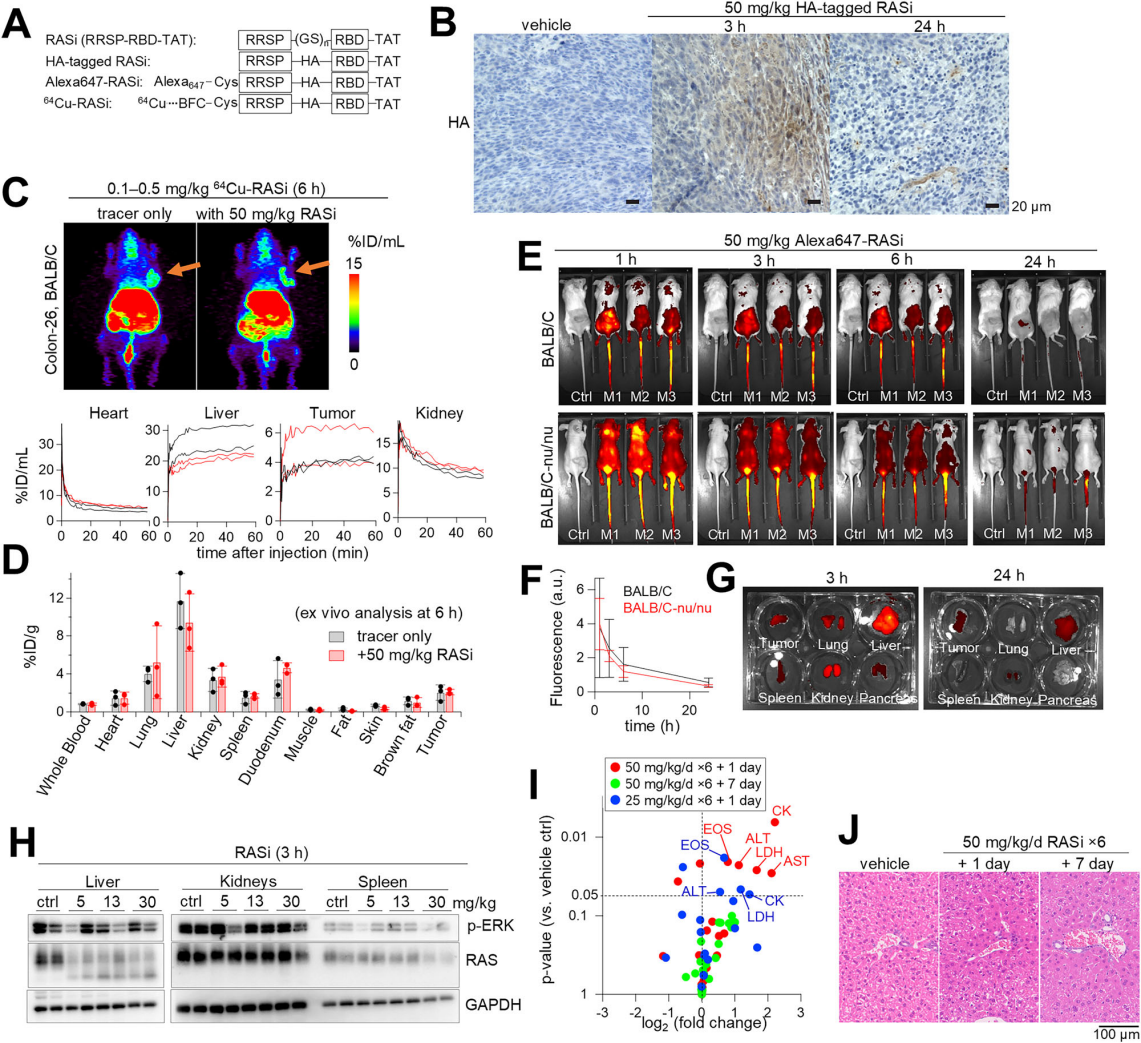
Pharmacokinetics, biodistribution, and safety profile of RRSP-RBD-TAT. **A** Schematic representation of the protein structure used for PK analyses. **B** IHC images of tumors in the Colon-26 syngeneic model treated with 50 mg/kg HA-tagged RRSP-RBD-TAT (RASi). Representative images from one experiment; similar findings were observed across the analyzed mice in the cohort. **C** Representative maximum intensity projection PET images obtained 6 h after administration of a ^64^Cu-RASi (0.1–0.5 mg/kg) with/without non-radioactive RASi (50 mg/kg). The arrow indicates the location of the subcutaneously transplanted tumor. Scale bars represent the injected dose per milliliter (ID/mL). The bottom panel shows the time course of radioactivity levels in various tissues (0.5 mg/kg ^64^Cu-RASi only, black; 0.5 mg/kg ^64^Cu-RASi plus 50 mg/kg non, red). **D** Ex vivo radioactivity levels in various tissues excised 6 h after administration (0.1 mg/kg, black; 50 mg/kg, red). Data are mean ± SD (*n* = 3 per group). **E** Fluorescence imaging of Alexa647-RASi following administration in Colon-26 syngeneic and immunodeficient nu/nu models. **F** Quantification of tumor fluorescence changes from Fig. 5E (BALB/C, black; BALB/C-nu/nu, red). Data are mean ± SD (*n* = 3 per group). **G** Representative ex vivo fluorescence imaging of various tissues excised 3 and 24 h after administration. **H** Western blot analysis of liver, kidney, and spleen lysates from non-tumor-bearing C57BL/6 mice, 3 h after a single RASi injection. Representative immunoblots from two independent experiments with similar results. **I** Blood test results from non-tumor-bearing C57BL/6 mice treated with RASi at 50 or 25 mg/kg/day for six doses. Blood was collected 1 day or 1 week after the final dose. Parameters with significant differences (*p* < 0.05, unpaired two-tailed t-test) vs. vehicle control are highlighted. **J**H&E-stained images of liver sections showing hepatocellular hypertrophy 1 day after the final dose. Representative images from one experiment; similar findings were observed across the analyzed mice in the cohort.

We next examined PK of RRSP-RBD-TAT in the Colon-26 syngeneic model using tagged proteins (Fig. 5A). Immunohistochemistry (IHC) of HA-tagged RRSP-RBD-TAT revealed intracellular localization in both tumor and adjacent normal cells within 3 h post-treatment (Figs. 5B and S21). PET analysis of 0.1–0.5 mg/kg ^64^Cu-labeled RRSP-RBD-TAT revealed that the tumoral drug concentration reached equilibrium within 5 min of injection at both tracer with/without non-radioactive RRSP-RBD-TAT (50 mg/kg) (Figs. 5C and S22). PET analysis further indicated that RRSP-RBD-TAT preferentially accumulates in the liver, followed by the kidney, lung, and duodenum, and to a lesser extent in the tumor, spleen, and heart (Fig. 5D), in line with the typical biodistribution of 60–80 kDa macromolecules^24^. Nevertheless, the injected dose per milliliter (ID/mL) in the tumor region reached approximately 4% at 1–6 h, corresponding to an intratumoral concentration of approximately 0.86 µM when administered at 50 mg/kg. Although this concentration is comparable to in vitro EC50 values (Fig. 3A), it is sufficient for anti-tumor activity in vivo because the inhibitor localizes intracellularly (Fig. 5B), while over 90% remained in the extracellular space in vitro^9^. Fluorescence imaging with Alexa-647-labeled RRSP-RBD-TAT corroborated the IHC and PET analyses, both in vivo and ex vivo (Figs. 5E, F, G and S23). Moreover, comparing PK profiles in immunocompetent Colon-26 and athymic nu/nu models revealed no significant differences in tumor drug retention half-lives (5.4 ± 1.0 vs 4.3 ± 0.75 h; Fig. 5F). This finding aligns with our observation that IFNγ^+^ CD8^+^ T cell infiltration, rather than PK, is the principal driver of drug sensitivity. Overall, these data demonstrate that RRSP-RBD-TAT possesses a favorable PK profile for cancer therapy.

We next evaluated the toxicity profile in the tissues where RRSP-RBD-TAT preferentially accumulated. Immunoblot analysis indicated that RRSP-RBD-TAT cleaves RAS in the liver with efficacy similar to that observed in tumors (Fig. 3J), but it did not affect RAS in kidneys or spleen (Fig. 5H). Blood tests performed one day after the final dose in the treatment regimen (25 or 50 mg/kg/day × 6 doses) showed transient elevations of AST, ALT, LDH, CK, and eosinophils—up to fourfold higher (Fig. 5I, Supplementary Data 3). However, these parameters returned to baseline within a week. Histopathological analysis of the liver, kidneys, and spleen revealed only mild, well-tolerated adaptive changes, including hepatocellular hypertrophy and basophilic tubules (Figs. 5J and S24). Furthermore, in vivo redox MRI detected faster oxidation of nitroxyl radicals in the liver at 3 h post-injection, which reverted after 1 day (Fig. S25). Altogether, these observations indicate that while RRSP-RBD-TAT accumulates in the liver and cleaves RAS, any resulting tissue damage is transient and tolerable.

## Discussion

In this study, we have developed a protein-based pan-RAS inhibitor, RRSP-RBD, by combining an RBD with RAS-targeting PTM enzymes. Initially, we discovered that incorporating the RBD into these enzymes—particularly RRSP—overcame their low intrinsic RAS-binding affinity, which enabled synergistic and potent RAS inhibition (Fig. 1). RRSP‑RBD operates as a pan-RAS (K/H/N-RAS) inhibitor (Figs. 1F, 2A, 3A and S3), because the RBD binds K/H/N-RAS across wild‑type and multiple oncogenic mutants^9^, while RRSP recognizes Switch I and cleaves RAS between Y32–E33^15^, a motif conserved among K/H/N‑RAS (Fig. S6). Subsequently, we developed two cell-permeable variants—RRSP-RBD-DTB and RRSP-RBD-TAT. The DTB-based construct demonstrated femtomolar anti-tumor potency against various RAS-mutant cancer cells and induced tumor regression in a KRAS-G12V mutant xenograft mouse model (Fig. 2), thereby providing in vivo proof-of-concept for RRSP-RBD as a pan-RAS inhibitor. However, this construct is effective only in human cells, which limits evaluations of toxicity, PK, and interactions with anti-tumor immunity in mouse models. To overcome this limitation, we developed CPP-based construct, RRSP-RBD-TAT (Fig. 3), which also exhibited potent anti-tumor potency in vivo, achieving a 60% complete tumor regression rate in a CT-26 syngeneic mouse model (Fig. 3E), comparable to the efficacy of AMG-510 (80%)^25^ and RMC-6236 (60%) in the same model^3^.

Detailed analysis of RRSP-RBD-TAT in the context of anti-tumor immunity revealed a marked dependence on IFNγ and CD8^+^ T cells for tumor-specific necrosis (Fig. 4E, F)—a phenomenon not observed with small-molecule RAS inhibitors. It is well established that, in addition to its cell-autonomous effects, RAS signaling exerts non-cell-autonomous influences on the TME by modulating cancer-associated fibroblasts (CAFs), extracellular matrix (ECM), and immune cell infiltration^26^. Consequently, many RAS inhibitors showed enhanced efficacy in immunocompetent models^8^. However, the mechanisms by which these agents enhance anti-tumor efficacy appear to differ; small-molecule RAS inhibitors have not shown a strict dependence on IFNγ and CD8^+^ T cells. For example, KRAS-G12C inhibitors like AMG510 promote CD8^+^ T cell infiltration and macrophage activation in immunocompetent models, driving durable tumor regression^25^. Similarly, KRAS-G12D inhibitors remodel CAFs/ECM while increasing CD8^+^ T cell infiltration^27,28^. Pan-RAS inhibitors such as RMC-6236 have also been shown to augment T cell infiltration and MHC class II expression within tumors^3^. The basis for the apparent discrepancies between these agents and RRSP‑RBD‑TAT remains unclear at this stage.

However, at least three non-mutually exclusive possibilities can be considered. First, RRSP‑RBD‑TAT is a pan‑RAS inhibitor that targets K/H/N‑RAS (Fig. S3), but it may also cleave noncanonical RAS‑family members such as MRAS or Rap1A, potentially eliciting a distinct pattern of immune activation. In this regard, the sequences surrounding the RRSP substrate recognition site (around Y32 and D33) are conserved in Rap1 and MRAS, whereas the C‑Raf RBD has been reported to bind Rap1 and MRAS with ~10–100‑fold lower affinity than canonical RAS isoforms^29,30^. Thus, RRSP‑RBD‑TAT is expected to act preferentially on K/N/H‑RAS, but a definitive assessment of its full target spectrum will require proteomics‑based analyses. Second, RRSP‑RBD‑TAT cleaves RAS between Y32 and D33, and the resulting RAS fragments may themselves trigger secondary immune responses distinct from those induced by small‑molecule inhibitors that merely block RAS activity without generating such cleavage products. Finally, the PK properties of RRSP-RBD-TAT as a cell-permeable macromolecule may uniquely modulate the tumor immune landscape.

RRSP-RBD-TAT displayed promising pharmacokinetic and safety profiles for cancer therapy. It accumulates efficiently in tumors (Fig. 5C, D, F) and is delivered intracellularly (Fig. 5B), underscoring its robust in vivo anti-tumor efficacy. Consistent with the typical biodistribution of 60–80 kDa macromolecules^24^, RRSP-RBD-TAT also localized to the liver and kidneys, and cleaved RAS in liver (Fig. 5D, G, H). Nevertheless, any resulting tissue damage was mild and transient (Fig. 5I, J), likely reflecting the low dependence of normal tissues on RAS signaling for survival and/or minimal infiltration by IFNγ^+^ CD8^+^ T cells. These findings are significant because they support the overall safety of pan-RAS inhibition, including RRSP-RBD-DTB and other emerging pan-RAS inhibitors such as RMC-6236.

## Methods

### Ethical statement

All mouse experiments were approved by the Committees for Animal Research and Welfare of Gifu University and Tokushima University (protocol nos. 2019-168, 2021-142, and 2024-126) and conducted in accordance with institutional guidelines. The maximal tumor burden permitted by the institutional animal ethics committees was 2000 mm³, and this limit was not exceeded in any experiment.

### Antibody and cell lines

Antibodies and cell lines used and their sources are listed in key resource files.

### Protein expression and purification

Genes encoding the target proteins were synthesized by gBlocks (Integrated DNA Technology) or GeneArt (Thermo Fisher) and cloned into the pRSET-A vector. RRSP (aa 3596–4080)^14^, TpeL (aa 1–542)^31^, or ExoS (aa 233–455)^32^ were each fused with cRaf-v1 RBD (aa. 51–131)^9^ via a GSSGGGGSGGGSSMG linker at the C-terminus, then further linked with either TAT(47–57, YGRKKRRQRRR) or diphtheria toxin (aa 180–535)^20^ using a 15 amino acids GS linker at the C-terminus. Inactivating mutations (RRSP*, H4030A^14^; TpeL*, D286A/D288A^31^; ExoS*, E379A/E381A^32^; cRaf-v1 RBD*, R88A/H89A^9^) were introduced by inverse PCR, as necessary. Plasmids generated in this study are available from the corresponding author.

Plasmids were transformed into *E. coli* BL21(DE3) and cultured in LB medium containing ampicillin. Protein expression was induced at OD600 ~ 0.6 by adding 0.1 mM IPTG, followed by shaking overnight at 16 °C. Bacterial cells were harvested and resuspended in buffer A (50 mM Tris-HCl, pH 8.0, 300 mM NaCl), then lysed using a VP-300N ultrasonic processor (TAITEC, #0075955-001). After sequential centrifugation at 3410 × *g* (30 min) and 30,000 × *g* (30 min) at 4 °C, the supernatant was collected and applied to Ni Sepharose 6 Fast Flow resin (Cytiva). The resin was washed three times with W1 buffer (buffer A plus 1% Triton X-114 and 20 mM imidazole) for endotoxin removal, three times with W2 buffer (buffer A plus 1.7 M NaCl and 20 mM imidazole), and twice with W3 buffer A plus 20 mM imidazole). Subsequently, the protein was eluted with elution buffer (buffer A plus 500 mM imidazole). For screening proteins (Fig. S6), eluates were dialyzed twice against PBS and used for in vitro cytotoxicity assay.

Eluates were further purified by size-exclusion chromatography using ProteoSEC 16 mm × 60 cm 6–600 kDa columns (M&S TechnoSystems) or Superdex™ 200 Increase 5/150 GL columns (Cytiva) in D-PBS(−) running buffer. Purified proteins were concentrated using Amicon® Ultra Centrifugal Filters (30 kDa MWCO), filtered through 0.22 µm filters, and stored at −80 °C. Protein quality was verified by MALDI-TOF-MS (MALDI-8030, Shimadzu), CBB-stained SDS-PAGE, and thermal denaturation assay coupled with circular dichroism. Endotoxin levels were measured using the ToxinSensor Chromogenic LAL Endotoxin Assay Kit (GenScript #L00350C) at protein concentrations of 1–100 µg/mL in D-PBS(−).

### Circular dichroism (CD) and thermal denaturation experiments

The secondary structure of 50 µg/mL PTMe-RBD was assessed at 20 °C using a Chirascan-plus CD spectrometer (Applied Photophysics) with a 1 mm path-length quartz cuvette (Hellma, 100-QS). The protein was diluted from stock solutions into a low-salt buffer (10 mM sodium phosphate, pH 7.2). After recording the spectra at 20 °C, the temperature was increased to 94 °C at a rate of 4 °C/min, and changes in the CD signals were monitored.

### Cell viability assay

Cells were maintained in DMEM (High Glucose) (Wako 043-30085) supplemented with 10% FBS in a 37 °C incubator with 8% CO_2_. For viability assays, cells were seeded in 96-well plates (Nunc™ Edge™ 96-Well, Nunclon Delta-Treated, Flat-Bottom Microplate) and cultured in RPMI-1640 (Wako 189-02025) plus 10% FBS. The following day, media containing inhibitors at twice the desired final concentration were prepared and mixed 1:1 with the existing medium. After 3 days, cell viability was measured using the Cell Counting Kit-8 (Dojindo). EC50 values and the area under the curve (AUC) were calculated from dose-response plots, obtained from three replicates.

### NanoBRET assay

Genes encoding KRAS and PTMe-RBDs were cloned into pFN31K Nluc CMV-neo Flexi® and pFC14K HaloTag® CMV Flexi® vectors, respectively. HEK293T cells were then transfected using Lipofectamine 3000. After 24 h, HaloTag NanoBRET 618 (Halo618) was added according to the manufacturer’s protocol. On the next day, luminescence was measured using the NanoBRET® Nano-Glo® Detection System (N1661, Promega). Signals at 485 nm and 620 nm were detected using a SpectraMax iD5 (Molecular Devices), and BRET ratios were calculated.

### Confocal imaging

Genes encoding KRAS and PTMe-RBD were cloned into pcDNA3.1( + ). 3×FLAG or 2×HA tag was introduced by inverse PCR.These vectors were transfected into HEK293T cells using Lipofectamine 3000, grown on poly-L-lysine-coated coverslips. The following day, cells were washed with PBS, fixed with 4% paraformaldehyde for 15 min at room temperature, and washed three times with PBS. The cells were then blocked with PBS containing 0.3% Triton X-100 and 5% BSA for 60 min at room temperature, incubated with primary antibodies in PBS + 1% BSA for 60 min at room temperature, washed three times with PBS, and incubated with secondary antibodies in PBS + 1% BSA for 60 min at room temperature in dark. Following three additional PBS washes, cells were mounted on glass slides with SlowFade™ Diamond Antifade Mountant with DAPI (Thermofisher). Images were captured using a Zeiss LSM910 confocal laser microscope in Airyscan 2 mode.

### Western blotting

For intracellular expression experiments (Fig. 1E), pcDNA3.1 plasmids encoding RRSP‑RBD‑2×HA were transfected into PANC‑1 cells using Lipofectamine 3000 according to the manufacturer’s instructions. After 24 h, the medium was replaced with DMEM + 10% FBS and cells were incubated for an additional 72 h. Cells were washed once with ice‑cold PBS and lysed in SDS sample buffer (2% SDS, 62.5 mM Tris‑HCl [pH 6.8], 10% glycerol). Protein concentrations were determined with the Protein Assay Rapid Kit Wako II using bovine serum albumin as the standard. Lysates were normalized for total protein and processed for western blotting as described previously^9^. Band intensities were quantified in ImageJ.

For isoform co‑expression experiments (Fig. S3), HEK293T cells were co‑transfected with pcDNA3.1 RRSP‑RBD‑2×HA and pcDNA3.1 constructs encoding HRAS‑WT, HRAS‑G12V, KRAS‑G12V, or NRAS‑G12V. At 24 h post‑transfection, cells were washed and lysed as above, followed by immunoblotting as above.

For drug‑treatment experiments (e.g., Fig. 2C), cells were exposed to the indicated protein concentrations and durations prior to lysis, followed by immunoblotting as above.

### Microarray and GSEA

PANC-1 cells were treated with RPMI-1640 + 10% FBS containing 250 nM RRSP-RBD-TAT, RRSP-TAT, or vehicle for 48 h. RNA extraction and expression profiling were performed at Gifu University Division of Genomics Research, using Agilent SurePrint G3 Human GE 8 × 60 K Ver. 3.0 Microarray. For GSEA, gene expression data were queried against the MSigDB v7.5.1 database for the collections of hallmark gene sets (H) and oncogenic signatures (C6) with 1000 permutations (gene set) on GSEA 4.2.2 using default parameters.

### Mouse experiments

All in vivo experiments were performed in female mice. Sex was not evaluated as an experimental variable in this study, as a single sex was used consistently across models to minimize inter-experimental variability. A suspension of 3–15 × 10^6^ cancer cells in 100 μL DPBS(−) was injected subcutaneously into the right flank of 6–8-week-old BALB/C, C57BL/6, or BALB/C-nu/nu mice (Japan SLC). For intramuscular transplantation models, CT-26 cells were injected into the right thigh of BALB/C mice. Animals were randomized 4–16 days post‑inoculation when the mean tumor volume reached ~60 mm³ (or ~120 mm³ for delayed‑start cohorts, as specified), and assigned to receive vehicle or protein formulations via the tail vein (*n* ≥ 5 per group). Proteins were prepared in DPBS(−) at 2–10 mg/mL. Tumor volume was calculated using the formula: major axis × (minor axis)²/2. Tumor growth rate was defined as the ratio of the tumor volume difference (one day after the final dose vs. pre-treatment) in the treated vs. vehicle group. Mice were maintained in a specific pathogen-free (SPF) environment with constant temperature and humidity, a 12-h light/dark cycle, and free access to food and water.

### Depletion experiments using specific antibodies

Subcutaneous syngeneic models of Colon‑26 (BALB/c) or MC‑38 (C57BL/6) were established. On day 8 (Colon‑26) or day 12 (MC‑38) post‑inoculation, mice (*n* ≥ 4 per group) were injected intraperitoneally with depleting/neutralizing antibodies twice, 24 h apart at the following doses: anti-asialo‑GM1, 1 mg/kg; anti‑CD4, 7.5 mg/kg; anti‑CD8, 7.5 mg/kg; isotype‑matched IgG control, 7.5 mg/kg; anti‑TNFα, 10 mg/kg; anti‑IFNγ, 10 mg/kg. One day after the second antibody dose, RRSP‑RBD‑TAT was administered intravenously on two consecutive days at 50 mg/kg/day for Colon‑26 cohorts or 125 mg/kg/day for MC‑38 cohorts. Tissues were collected 3 h (Colon‑26) or 24 h (MC‑38) after the final RRSP‑RBD‑TAT dose. Splenic lymphocytes were isolated and analyzed by flow cytometry as described below. Tumor sections were H&E‑stained for necrotic‑area quantification and immunostained for CD4 and CD8.

### Tissue collection and processing

Tumor, blood, liver, kidneys, and spleen were collected under isoflurane anesthesia. Blood samples were collected into heparin-coated tubes and analyzed by FUJIFILM VET Systems Co., Ltd., for blood analysis. For immunoblotting, tissue samples were homogenized in SDS buffer (2% SDS, 62.5 mM Tris-HCl [pH 6.8], 10% glycerol) at 20 µL/mg tissue using a disposable pestle, and immediately boiled at 98 °C for 5 min. Protein concentrations were quantified using the Protein Assay Rapid Kit Wako II with bovine serum albumin as a standard, diluted to 1 mg/mL, and analyzed by immunoblotting.

### Immunohistochemistry (IHC)

IHC was conducted as described previously^9^. Briefly, tissues were fixed in 4% paraformaldehyde, paraffin-embedded, sectioned into 2-µm-thick slices, deparaffinized in lemosol, and rehydrated through graded ethanol to water. Antigen retrieval was performed by autoclaving sections at 120 °C for 1 min in antigen retrieval buffer (pH 6.0, Matsunami Glass #IA6500). Sections were treated with 3% H₂O₂ in methanol, washed with distilled water, blocked with 2.5% normal horse serum (Vector Laboratories #S-2012), and incubated overnight with anti-HA antibody at 4 °C. After washing with TBST, sections were incubated with an anti-rabbit secondary antibody for 15 min and developed using ImmPACT DAB Substrate (Vector Laboratories #SK-4105). Section was counterstained with Hematoxylin 3 G (Sakura Finetek #8656), then dehydration, cleared with lemosol, and mounted with Mount-Quick (Daido Sangyo #DM01). Some sample preparation (paraffin-block making sectioning, staining) was outsourced to Morphotechnology Co., Ltd.

### PET analysis

As illustrated in Fig. 5A, the GS linker sequence in pRSET A-RRSP-RBD-TAT was replaced with an HA-tagged sequence (SLGSSGGGGSGGGSSMG → SLTMSGYPYDVPDYAGSMG) via inverse PCR. Subsequently, a cysteine mutation was introduced between the N-terminal His-tag and RRSP sequence (MRGSHHHHHHGMASLVPRGS-[RRSP] → MRGSHHHHHHGMACLVPRGS-[RRSP]). The plasmid was expressed in BL21(DE3) and purified using Ni resin and size-exclusion chromatography as described above. ^64^Cu-RASi was synthesized with a slight modification of the previous method for antibody labeling^33^, as illustrated in Fig. S22A–D. Briefly, purified protein (10 μM) in DPBS(−) was incubated with 0.5 mM TCEP at room temperature for 30 min. Next, 1 mM bromoacetamido-PEG3-azide (Sigma #QBD11217-100MG) was added and incubated for 2 h at room temperature. The protein was washed five times with DPBS(−) using an Amicon-30k, reacted with 3 equivalents of DBCO-PEG4-CBTE1K1P overnight at room temperature, and washed with distilled water using an Amicon-10k. Finally, [^64^Cu]CuCl_2_ (50.2 MBq/nmol of protein; PDRadiopharma Inc., Tokyo, Japan) was added and incubated at 37 °C for 1 h. The protein was washed three times with 0.2 M glycine buffer (pH 6.8) and three times with PBS-T using an Amicon-10k. The radiochemical purity and molar radioactivity at the end of synthesis were approximately 7.8 MBq/nmol and 94.7%, respectively.

PET studies were performed as described previously^34^, with slight modifications. During the PET scans, mice were anesthetized with 1.5% isoflurane. ^64^Cu-RASi (approximately 1 MBq, 0.5 mg/kg) was intravenously injected into Colon-26 syngeneic model mice with/without non-radioactive RASi (50 mg/kg). Emission data were immediately acquired for 60 min using a Siemens Inveon PET/CT (Siemens Co., Knoxville, TN, USA) in three-dimensional list mode. The emission data were sorted into 38 dynamic sinograms (6 × 10, 6 × 30, 11 × 60, and 15 × 180 s). Approximately 6 h after administration, the mice were again anesthetized and 15-min emission scans were performed. The emission data were converted into static sinograms. PET images were reconstructed by Fourier rebinning and standard 2-D filtered back projection using microPET manager (Siemens Co.). Time-activity curve analyses were performed using ASIPro VM image-processing software (Siemens Co.). For ex vivo measurements of radioactivity biodistribution, ^64^Cu-RASi (approximately 0.3–1.0 MBq, 0.1–0.5 mg/kg) was intravenously injected into mice with/without non-radioactive RASi (50 mg/kg). The radioactivity in blood and tissue samples was determined using the automatic gamma counter, corrected for decay from the time of injection, and converted the percentage of injected dose per gram of tissue (%ID/g) as described previously^34^.

### In vivo and ex vivo fluorescence imaging

The cysteine-mutant HA-tagged RRSP-RBD-TAT (10 μM) was prepared as described above, and incubated with 0.5 mM TCEP at room temperature for 30 min. Alexa Fluor 647 C2-maleimide (50 μM; Invitrogen #A20347) was then added and incubated for 2 h at room temperature in the dark. The protein was washed five times with DPBS(−) using Amicon-30k and concentrated to 10 mg/mL. Absorbance at 280 nm and 650 nm was measured, and labeling efficiency was calculated by:

1 $$\left[{protein}\right]=\frac{{A}_{280}-{A}_{650}\times 0.03}{55,330}$$

2 $$[{{{\rm{Alexa}}}}647]=\frac{{A}_{650}}{{{\mathrm{239,000}}}}$$

Labeling efficiencies ranged from 60% to 80%. For in vivo imaging, 50 mg/kg of Alexa 647-labeled RRSP-RBD-TAT was injected into Colon-26 syngeneic subcutaneous (BALB/C) or immunodeficient model (BALB/C-nu/nu), and fluorescence signals were obtained with IVIS Lumina S5 (Revvity #CLS148588) at 1, 3, 6, and 24 h after injection. For ex vivo imaging, tumor, lung, liver, spleen kidney and pancreas were resected 3 or 24 h after injection, then fluorescence signals were obtained with IVIS Lumina S5. The fluorescence signal was analyzed using Living Image Software (Revvity 128110). Tumor drug retention half-time was estimated by fitting fluorescence vs. time data to an exponential function.

For immunofluorescence (IF), tumors were resected 3 h after injection of 50 mg/kg Alexa 647-labeled RRSP-RBD-TAT. Tumors were fixed in 10% neutral buffered formalin and embedded in paraffin. Formalin-fixed, paraffin-embedded blocks were then sectioned into 4-µm sections using a microtome (Yamato Kohki Industrial #REM-710). Before staining, antigens were retrieved using Target Retrieval Solution (10×), pH 6.1 (Agilent #S1699) for HA-tag staining over 20 min at 98 °C. The sections were blocked with 4% Block Ace (KAC #UKB40) at room temperature for 30 min and incubated with HA-Tag (C29F4) Rabbit mAb (1/1000; CST #3724) at 4 °C overnight. The sections were then incubated with a Donkey anti-rabbit Alexa Fluor Plus 647 (1/200; Thermo Fisher #A32795) at room temperature for 1 h. Nuclei were stained with DAPI, and the sections were mounted with the ProLong Glass Antifade Mountant (Thermo Fisher #P36980). All images were obtained using a Virtual Slide Scanner (Olympus #VS-120).

### Hyperpolarized ^13^C pyruvate MRS and proton MR Imaging in CT-26 intramuscular syngeneic model

CT-26 intramuscular tumor-bearing mice in right thigh were treated with 50 mg/kg/day RRSP-RBD-TAT for six doses. Hyperpolarized ^13^C pyruvate MRS and proton MRI imaging was performed within 1–3 days after the final dose. For hyperpolarized ^13^C pyruvate MRS, hyperpolarization was achieved by using 15 μL of 14 M [1-¹³C]-pyruvate with 25 mmol/L of OX063 trityl radical. A 6.7 T SpinAligner polarizer (Polarize, Denmark) was operated at a microwave irradiation frequency of 188.030 GHz, 5 mW and 1.2 K for 40 min. Subsequently, the hyperpolarized probe solution was mixed with 3 mL of dissolution buffer containing 50 mmol/L trisaminomethane, 0.3 mmol/L EDTA, and 50 mmol/L NaOH. Following the dissolution process, the hyperpolarized probe solution was intravenously administered into the pre-anesthetized mice at a dose of 15 μL/g body weight. Spectral acquisition of ^13^C pyruvate and its downstream metabolites was performed for 100 s using a 1.5 T MRI scanner with a flip angle of 10 and a repetition time (TR) of 1 s.

Proton MRI was conducted using a 1.5 T MR VioLVA small animal MRI system (Japan Redox Ltd., Fukuoka, Japan). Mice were anesthetized with isoflurane (2% for induction and 1.0% for maintenance in 350 mL/min air). The mouse body was then fixed in a supine position on a customized holder using adhesive skin tape. The holder was inserted into the MR scanner inside a volume coil. Proton MR scanning was performed using gradient echo coronal or transverse 2D imaging. Proton images were acquired for the chest, abdomen, and tumor-bearing legs following a brief evaluation of the target ROI position using a fast low-angle shot pilot sequence. Six 2-mm-thick coronal or transverse T1-weighted images were obtained using a 64 * 64 matrix, 500 ms repetition time (TR), 10 ms echo time (TE), and four accumulations. Additionally, spin echo sequences with TR of 3000 ms, TE of 70 ms, two accumulations, six 2-mm slices, and a 64 * 64 matrix were used to obtain coronal and transverse T2-weighted proton images.

### Redox imaging using in vivo DNP-MRI

CT-26 subcutaneous syngeneic models were treated with 50 mg/kg/day RRSP-RBD-TAT once or twice. In vivo redox imaging was performed 3 h after the final dose with a low-field DNP-MRI system (Keller) obtained from Japan Redox Ltd. (Fukuoka, Japan). The external magnetic field B_0_ for EPR irradiation and MRI was fixed at 15 mT, and the radiofrequencies of EPR irradiation and MRI were 458 MHz and 689 kHz, respectively. A single-turn surface coil (inner diameter 19 mm) for EPR irradiation was used for tumor imaging in this study. During imaging, mice were anesthetized by isoflurane inhalation (4% for induction and 1.5% for maintaining anesthesia) in medical air (400 mL/min). The animal holder was placed in the center of the resonator, and the in vivo DNP-MRI scanning of the tumor-bearing legs was started immediately after the intravenous injection of carbamoyl-PROXYL (300 mM CmP in saline, 5 μL/g body weight). Pharmacokinetic DNP-MRI images were obtained at 0.5, 2, 4, 6, 8, 10, and 14 min after administration. Normal MRI images with 10 accumulations were obtained without EPR irradiation. The decay rate was calculated according to the intensity changes in tumor images during the time course from 0.5 to 14 min after injection of CmP. The pharmacokinetic images were used to obtain the in vivo redox map from the slope of the enhanced DNP image intensity in each pixel, using an Excel macro program. The scanning conditions for the in vivo DNP-MRI experiment were as follows: power of EPR irradiation, 5 W; flip angle, 90°; repetition time (TR) × echo time (TE) × EPR irradiation time (TEPR), 1000 × 20 × 500 ms; accumulation number, 1; slice thickness, 100 mm, including the whole thickness of the mouse; phase-encoding steps, 32; field of view (FOV), 60 × 60 mm; and matrix size, 64 × 64 after reconstruction. The in vivo DNP-MRI data were analyzed using ImageJ software.

### Measurement of tumor-infiltrating lymphocytes (TILs)

Tumors (~ 300 mm³) were excised, weighed, minced on ice with scissors and a scalpel, and passed through a 40-μm cell strainer. The filtrate was centrifuged at 400 × *g* for 5 min and resuspended in 5 mL RPMI-1640 + 10% FBS. Live cells was counted by trypan blue staining, centrifuged at 400 × *g* for 5 min, and the cell pellets were resuspended in 1 mL CELLBANKER® 1 (Takara #11910) and cryopreserved in liquid nitrogen. Cells were stained with a fixable viability dye (Life Technologies #65-0866-14) and surface antibodies. Intracellular staining was performed using intracellular antibodies and a fixation/permeabilization kit (eBiosciences #8-8824-00) in accordance with the manufacturer’s instructions. Data were acquired using a Fortessa™ flow cytometer (BD Bioscience), and FlowJo software (version 10.8.1 Tree Star Inc.) was used for all data analyses. Negative controls were included with irrelevant immunoglobulin G monoclonal antibodies. The detailed gating strategies for the lineage panel and T-cell IFNγ panel are provided in Supplementary Figs. S26 and S27, respectively.

### Measurement of tumor cytokines

Tumors (~ 300 mm³) were excised, snap-frozen in liquid nitrogen, and stored at −80 °C. For cytokine measurements, the samples were crushed into powder under liquid nitrogen and solubilized in RIPA buffer (Millipore, #20-188). Cytokine levels, including IFN-γ, IL-1β, IL-2, IL-6, IL-10, IL-12 (p70), VEGF-A, and TNF-α, were measured using the MILLIPLEX® Mouse Cytokine/Chemokine Magnetic Bead Panel (Millipore, #MCYTOMAG-70K-08C) and the Luminex 200 system, following the manufacturer’s protocol (Luminex, Austin, TX). Briefly, 25 µl of each sample extract was incubated with Premixed Beads overnight at 4 °C on a plate shaker. The beads were then washed and incubated with Detection Antibodies for 1 h at room temperature, followed by a 30-min incubation with Streptavidin-Phycoerythrin. After an additional wash step, the beads were resuspended in a wash buffer for 5 min and analyzed.

### 3D structure analysis

Structural analysis was based on the 3D structure of NRAS-G12V-GppNHp and cRaf-v1 complex (PDB: 6NTC). Contact interactions were identified using the Molecular Operating Environment (MOE, MOLSIS) in the Contact Analysis mode to pinpoint key residues involved in RAS binding.

### Reporting summary

Further information on research design is available in the Nature Portfolio Reporting Summary linked to this article.

## Supplementary information

Supplementary Information

Description of Additional Supplementary Files

Supplementary Data 1

Supplementary Data 2

Supplementary Data 3

Supplementary Data 4

Reporting Summary

Transparent Peer Review file

## Source data

Source Data

## Data Availability

The microarray data generated in this study have been deposited in ArrayExpress under accession code E-MTAB-16847. The published structural data used in this study are available in the Protein Data Bank under accession code 6NTC (NRAS-G12V-GppNHp in complex with cRaf-v1). The source data underlying the main figures and relevant Supplementary figures are provided as a Source data file. All other data supporting the findings of this study are available within the Article, Supplementary Information, and Supplementary data. Source data are provided with this paper.
