# Supplementary material for: Protein-based pan-RAS inhibitor induces tumor regression in female mice via IFNγ and CD8+ T cell-dependent tumor necrosis
Source: Nat Commun. 2026 May 16;17:6495. doi: 10.1038/s41467-026-73300-z (PMC13376784; doi:10.1038/s41467-026-73300-z)
Supplement: Supplementary file 1 — Supplementary Information [file 41467_2026_73300_MOESM1_ESM.pdf]

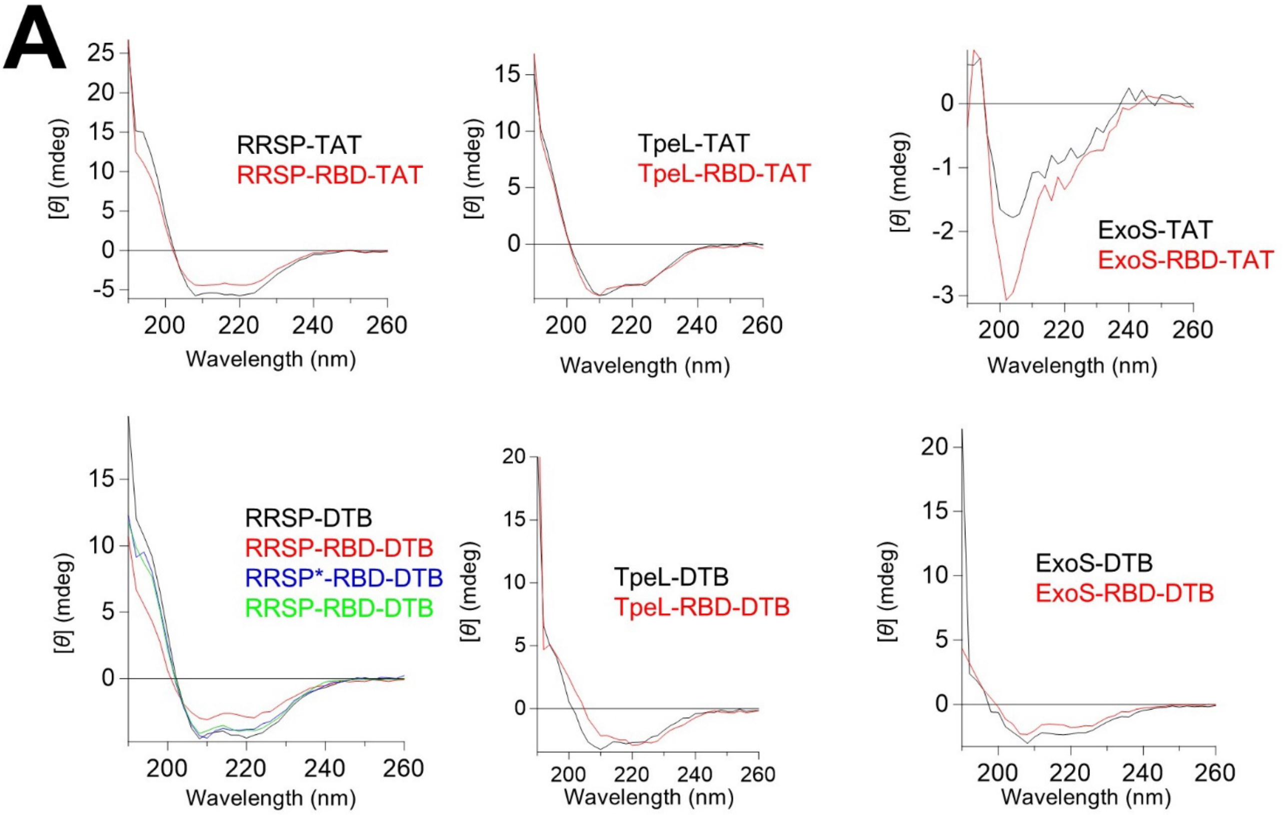
A
RRSP-TAT
RRSP-RBD-TAT
[θ] (mdeg)
Wavelength (nm)
TpeL-TAT
TpeL-RBD-TAT
ExoS-TAT
ExoS-RBD-TAT
RRSP-DTB
RRSP-RBD-DTB
RRSP*-RBD-DTB
RRSP-RBD-DTB
TpeL-DTB
TpeL-RBD-DTB
ExoS-DTB
ExoS-RBD-DTB

1

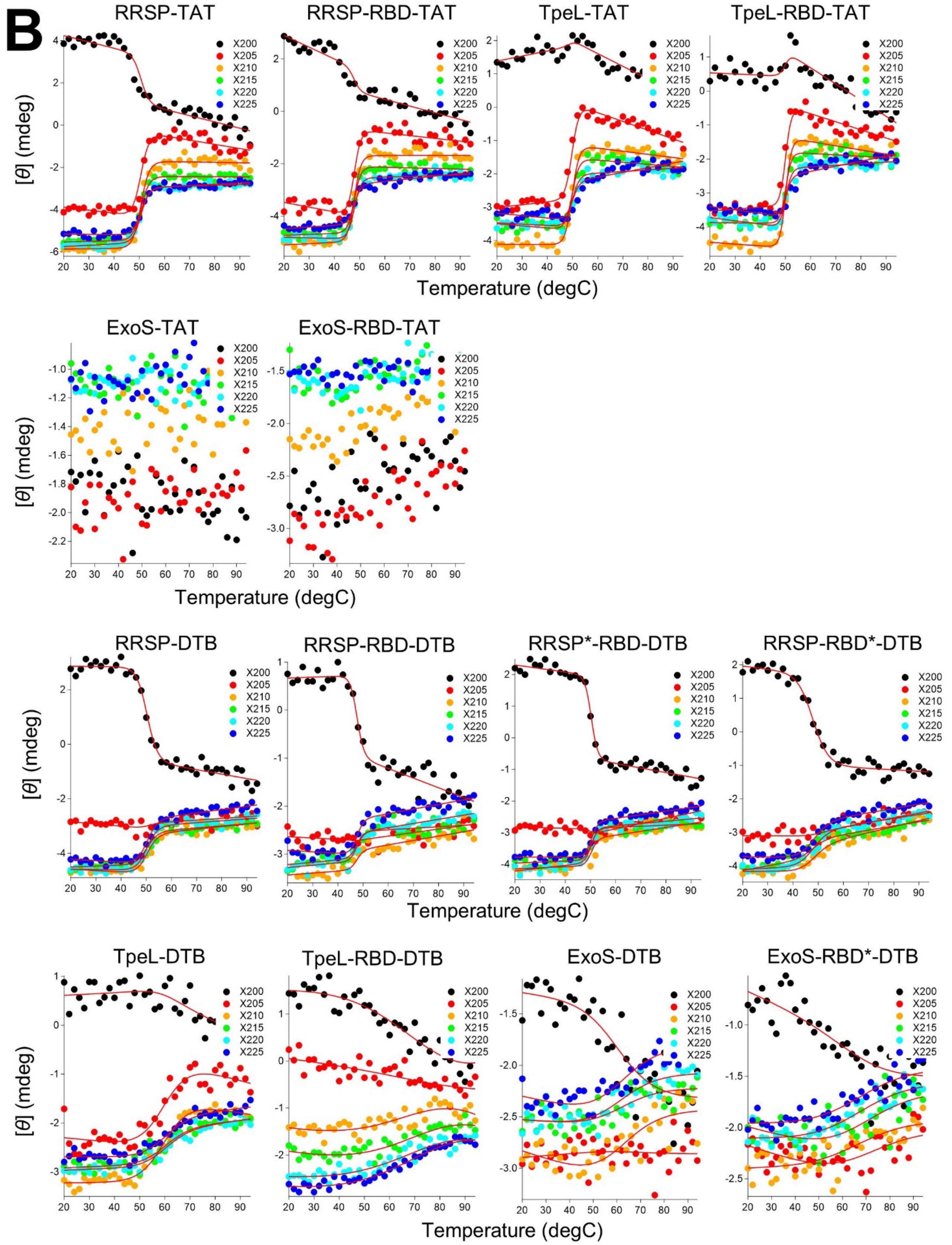

Figure S1. (A) CD spectra of PTMe-RBD proteins recorded at 20°C at a concentration of 50 μg/mL in a buffer composed of 90% 10 mM sodium phosphate (pH 7.2) and 10% DPBS(−). (B)

Thermal denaturation curves of PTMe-RBDs. Changes in CD signals were measured at six wavelengths (200, 205, 210, 215, 220, 225, 230 nm) with a heating rate of 4°C/min.

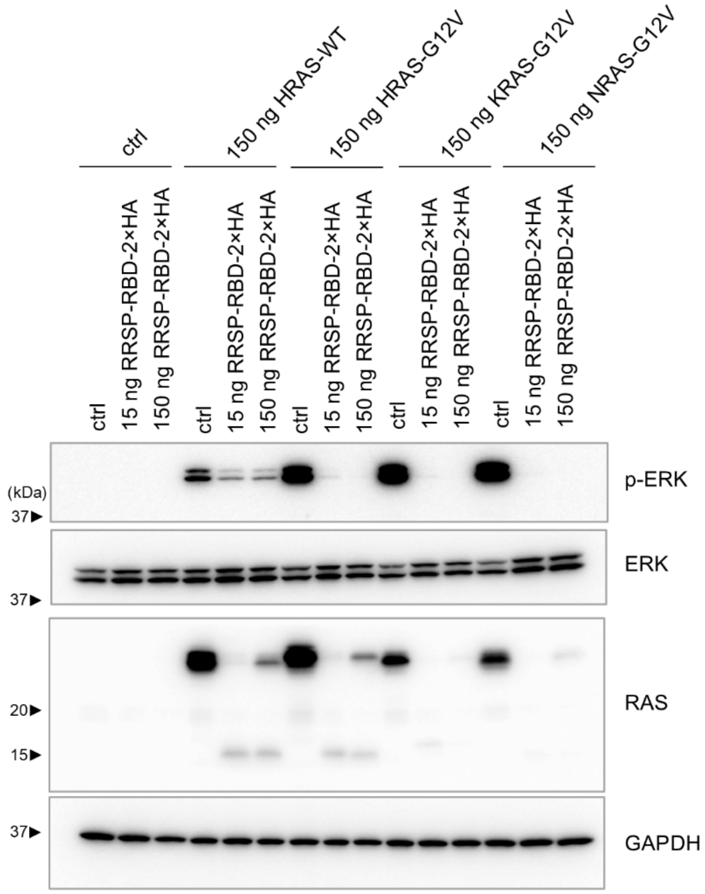

Figure S2. Immunoblot analysis of HEK293T lysates collected 24 h after co-transfection with RRSP-RBD-2×HA and the indicated RAS isoforms (HRAS-WT, HRAS-G12V, KRAS-G12V, NRAS-G12V) at the specified plasmid ratios. Across all isoforms tested, RRSP-RBD induced RAS cleavage and p-ERK suppression. Representative immunoblots from two independent experiments with similar results. Full uncropped blots are provided in the Source Data file.

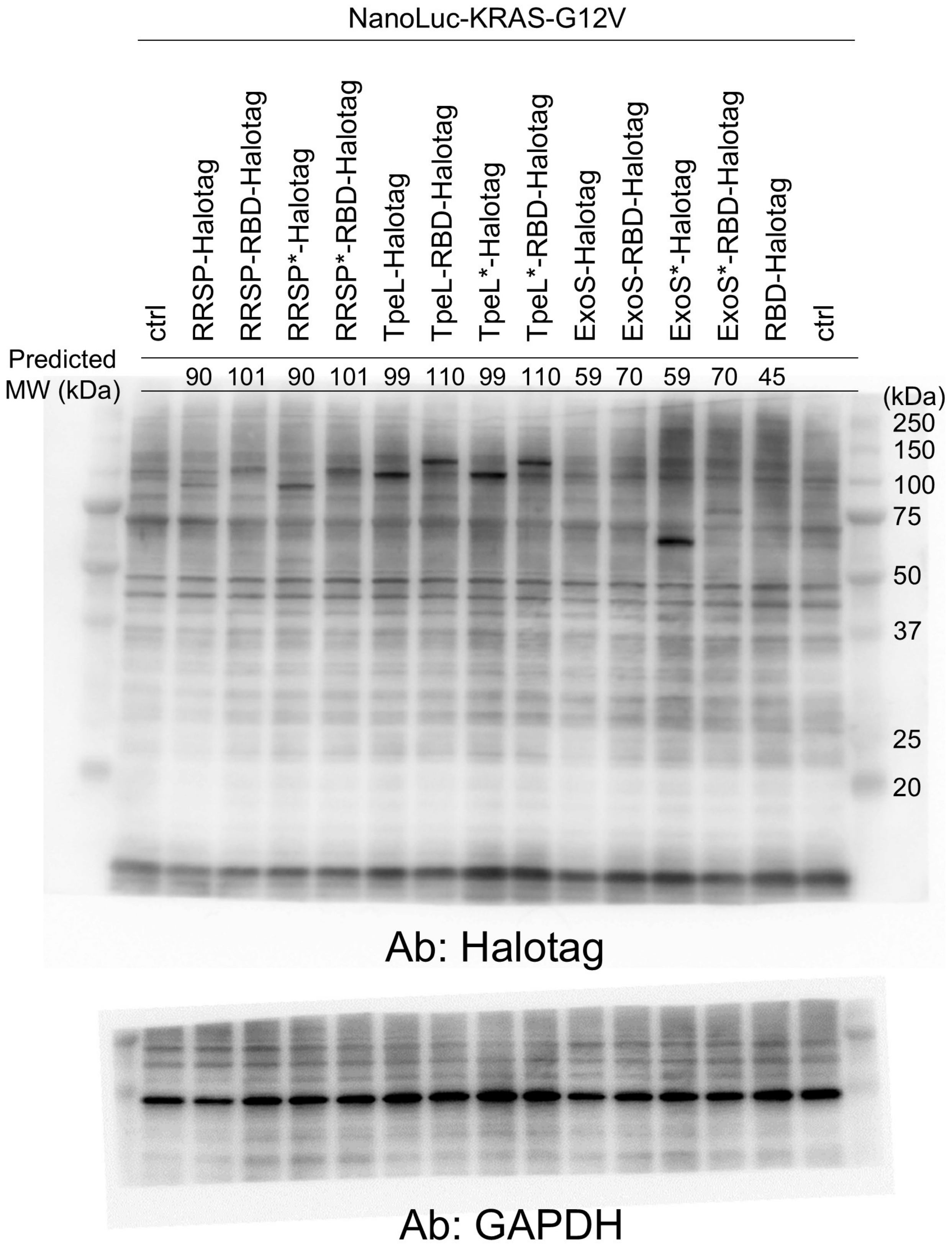

Figure S3. Western blot of HEK293T lysates following the NanoBRET assay in Figure 1F. Representative immunoblots from two independent experiments with similar results.

18

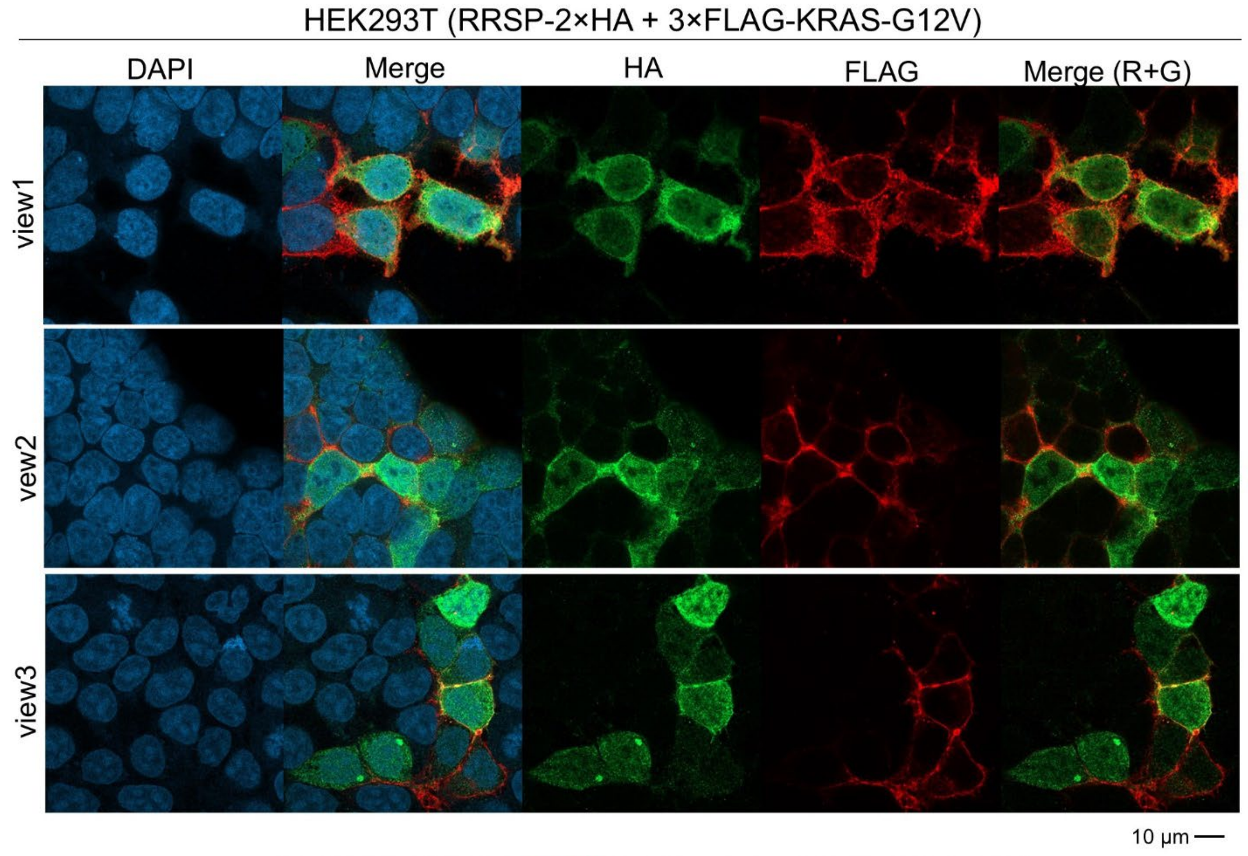
HEK293T (RRSP-2×HA + 3×FLAG-KRAS-G12V)
DAPI
Merge
HA
FLAG
Merge (R+G)
view1
vew2
view3
10 μm

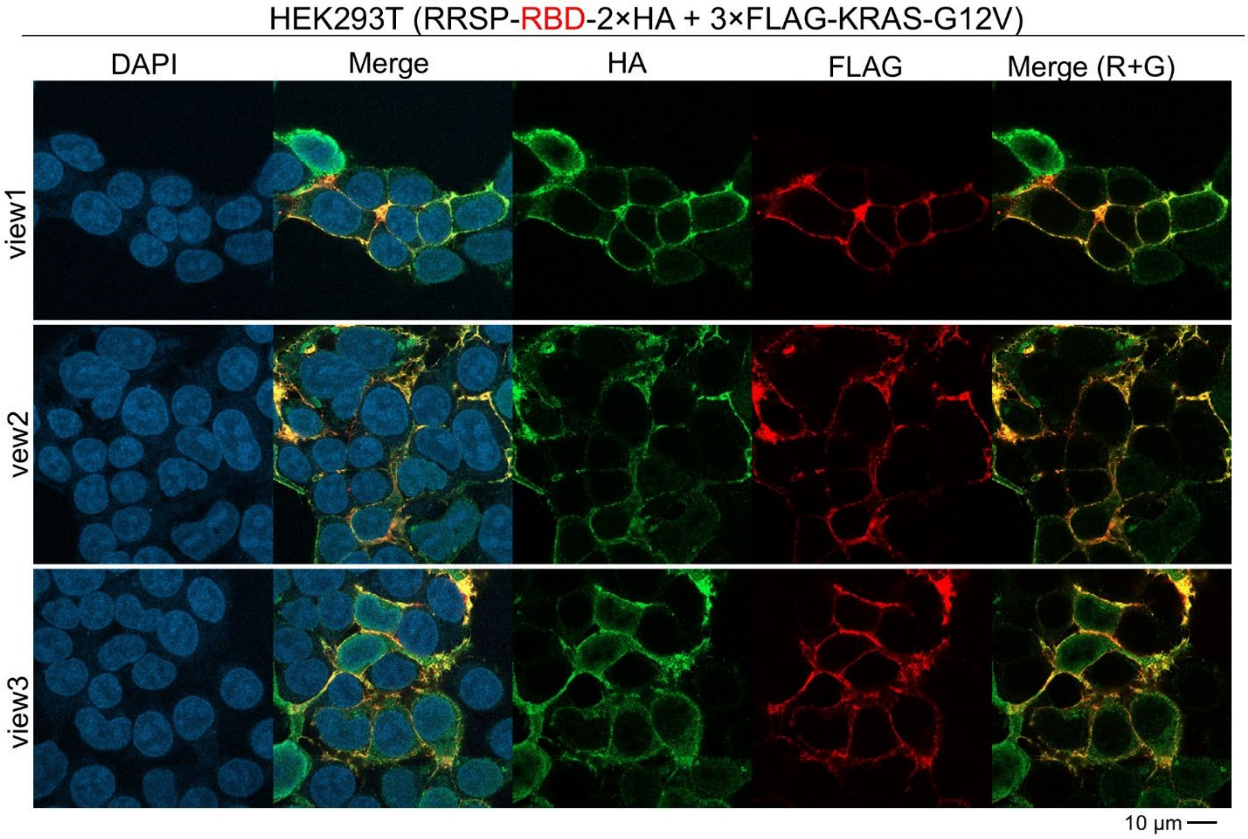
HEK293T (RRSP-RBD-2×HA + 3×FLAG-KRAS-G12V)
DAPI
Merge
HA
FLAG
Merge (R+G)
view1
vew2
view3
10 μm

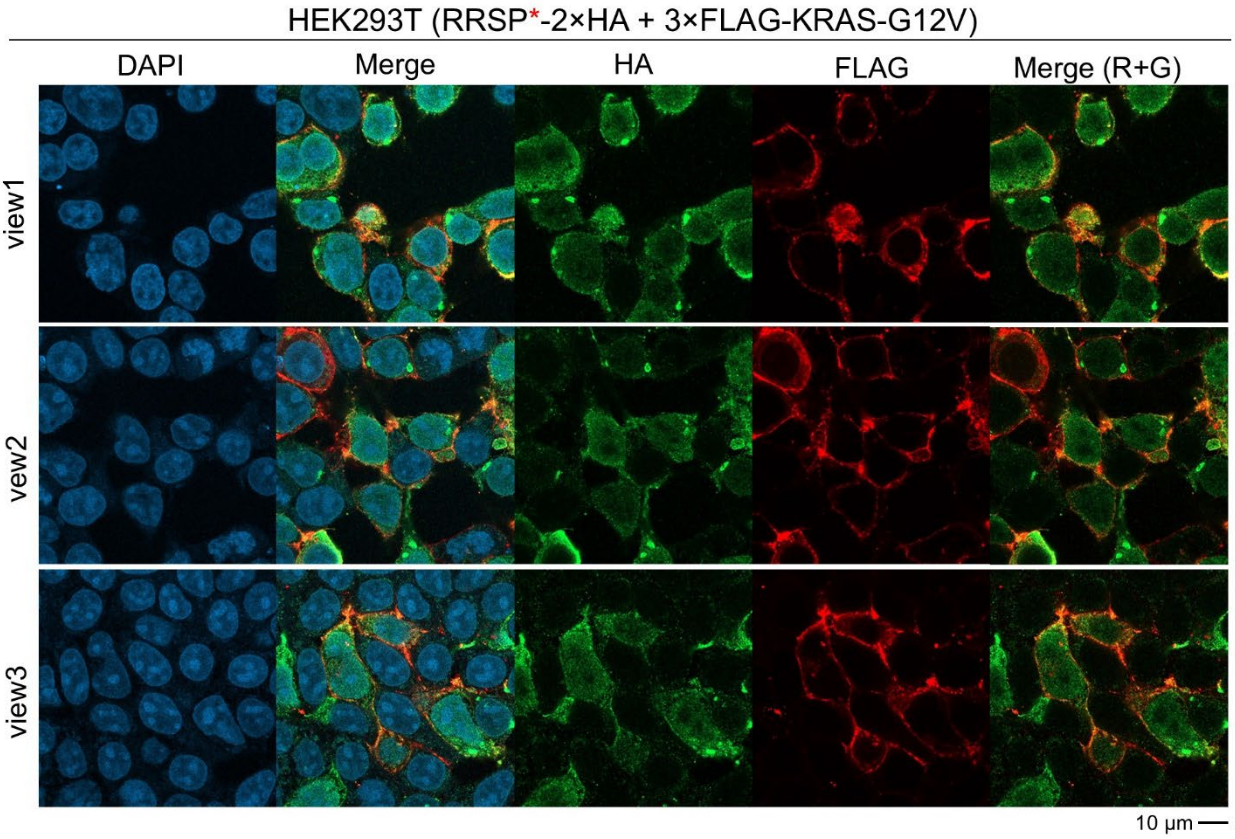
HEK293T (RRSP*-2×HA + 3×FLAG-KRAS-G12V)
DAPI
Merge
HA
FLAG
Merge (R+G)
view1
vew2
view3
10 μm

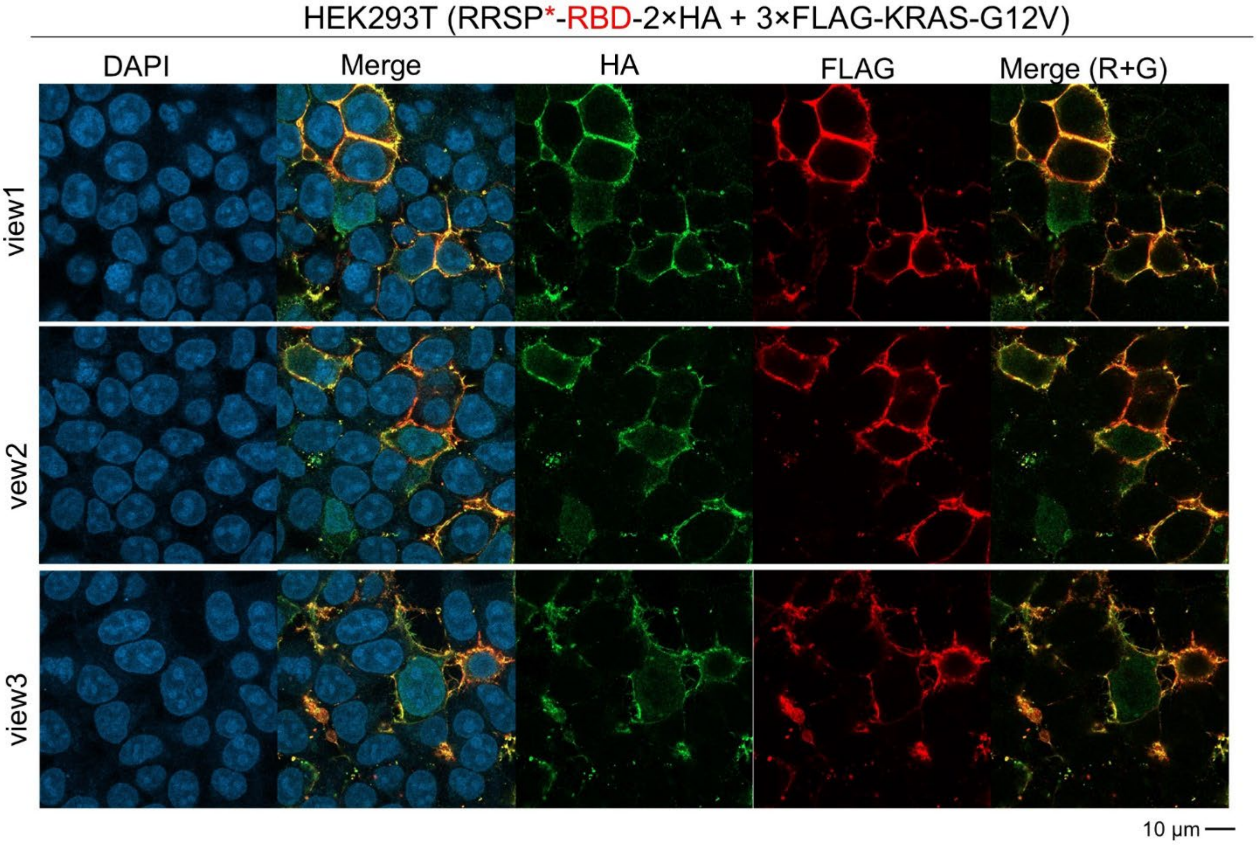
HEK293T (RRSP*-RBD-2×HA + 3×FLAG-KRAS-G12V)
DAPI
Merge
HA
FLAG
Merge (R+G)
view1
vew2
view3
10 μm

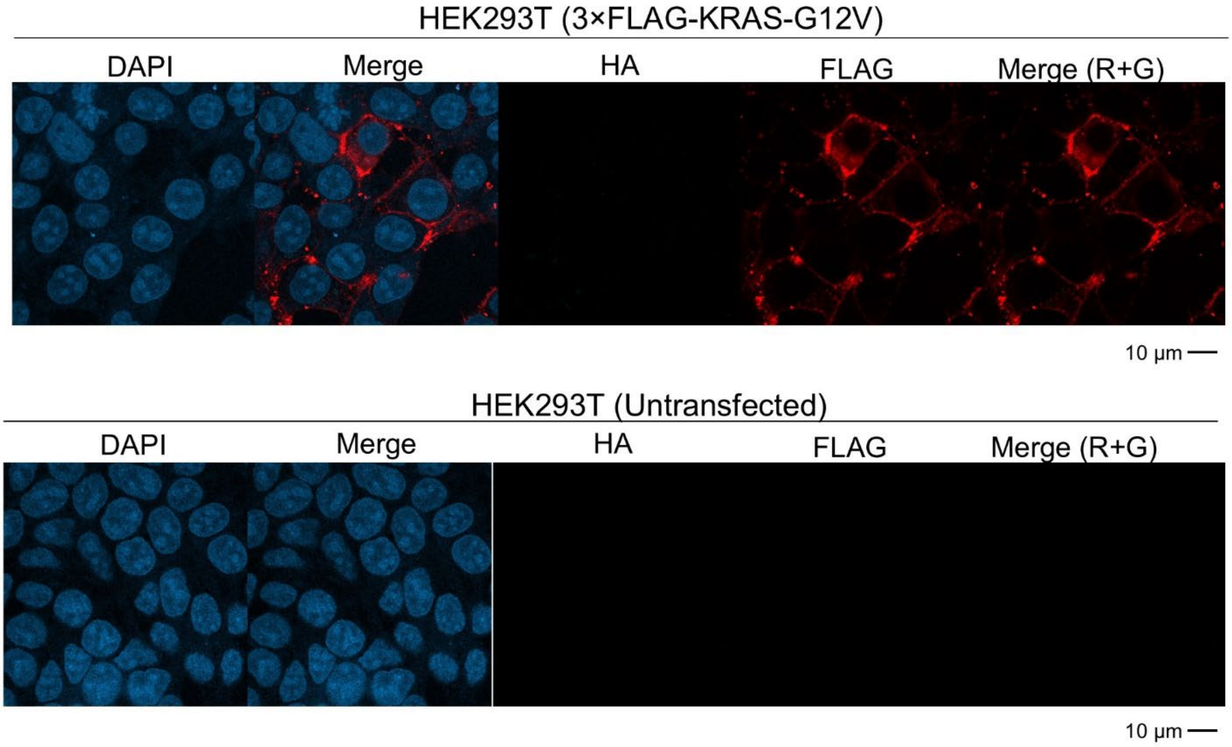

Figure S4. Confocal microscopy images from additional fields of view, showing the colocalization of RRSP-RBD-2×HA and 3×FLAG-KRAS-G12V in HEK293T cells. Representative images from two independent experiments with similar results.

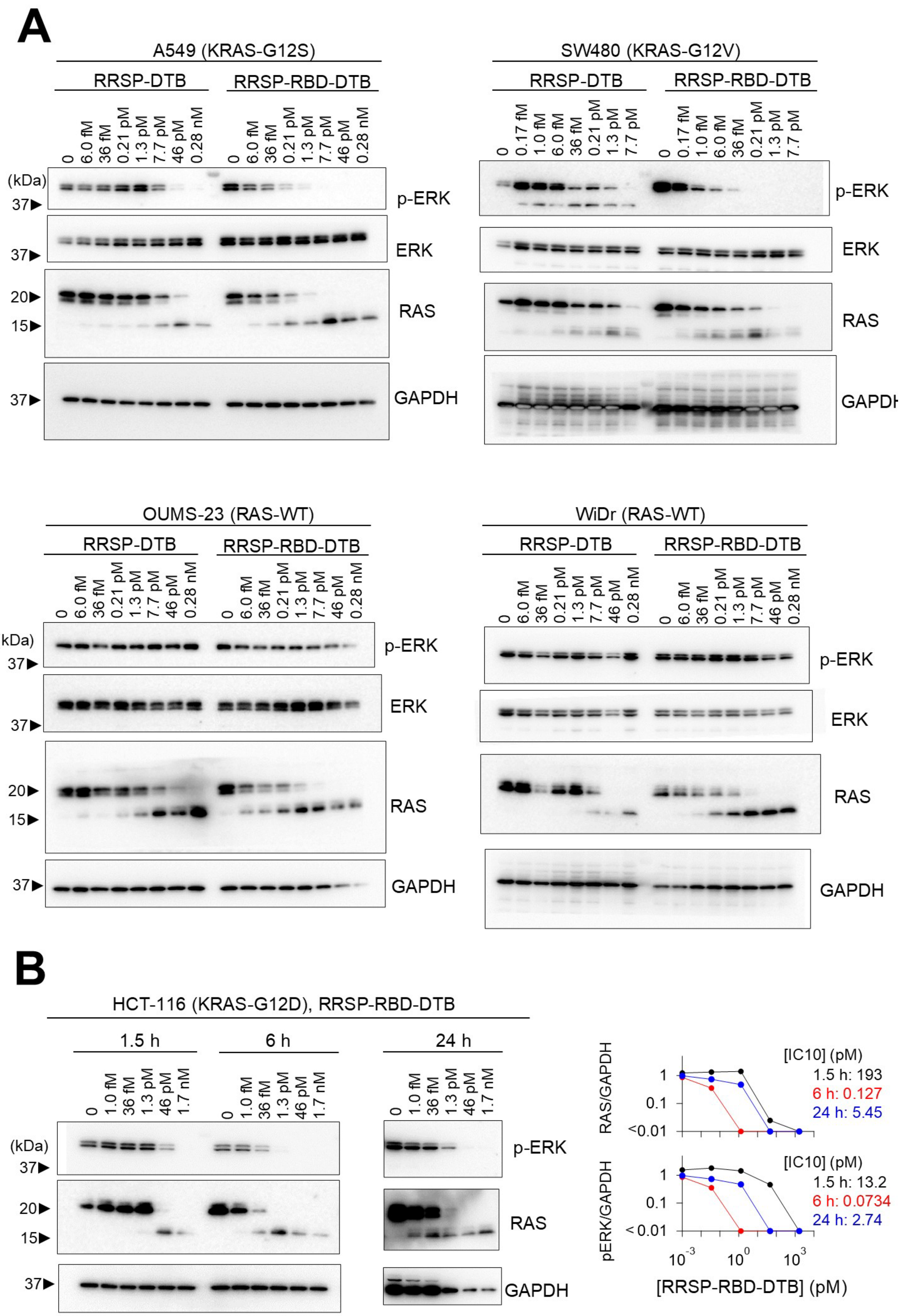
A
A549 (KRAS-G12S)
RRSP-DTB
RRSP-RBD-DTB
0
6.0 fM
36 fM
0.21 pM
1.3 pM
7.7 pM
46 pM
0.28 nM
(kDa)
37▶
20▶
15▶
p-ERK
ERK
RAS
GAPDH
SW480 (KRAS-G12V)
0.17 fM
1.0 fM
OUMS-23 (RAS-WT)
WiDr (RAS-WT)
B
HCT-116 (KRAS-G12D), RRSP-RBD-DTB
1.5 h
6 h
24 h
1.7 nM
RAS/GAPDH
pERK/GAPDH
[IC10] (pM)
1.5 h: 193
6 h: 0.127
24 h: 5.45
1.5 h: 13.2
6 h: 0.0734
24 h: 2.74
1
0.1
<0.01
[RRSP-RBD-DTB] (pM)

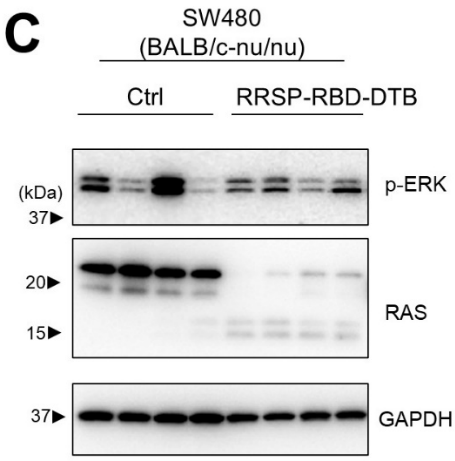

Figure S5. (A) Immunoblot analysis of lysates collected from cells treated with RRSP-RBD-DTB or RRSP-DTB at the indicated concentrations for 6 hours. (B) Immunoblot analysis of lysates from HCT-116 cells treated with RRSP-RBD-DTB at the indicated concentrations for 1.5, 6, or 24 hours. The right panels show band intensities vs. time, with the greatest effects at 6 hours. (C) Immunoblot analysis of tumor lysates collected 3 h after a single 10 mg/kg i.v. dose of RRSP-RBD-DTB in SW480 nu/nu mice. Representative immunoblots from two independent experiments with similar results. Full uncropped blots are provided in the Source Data file.

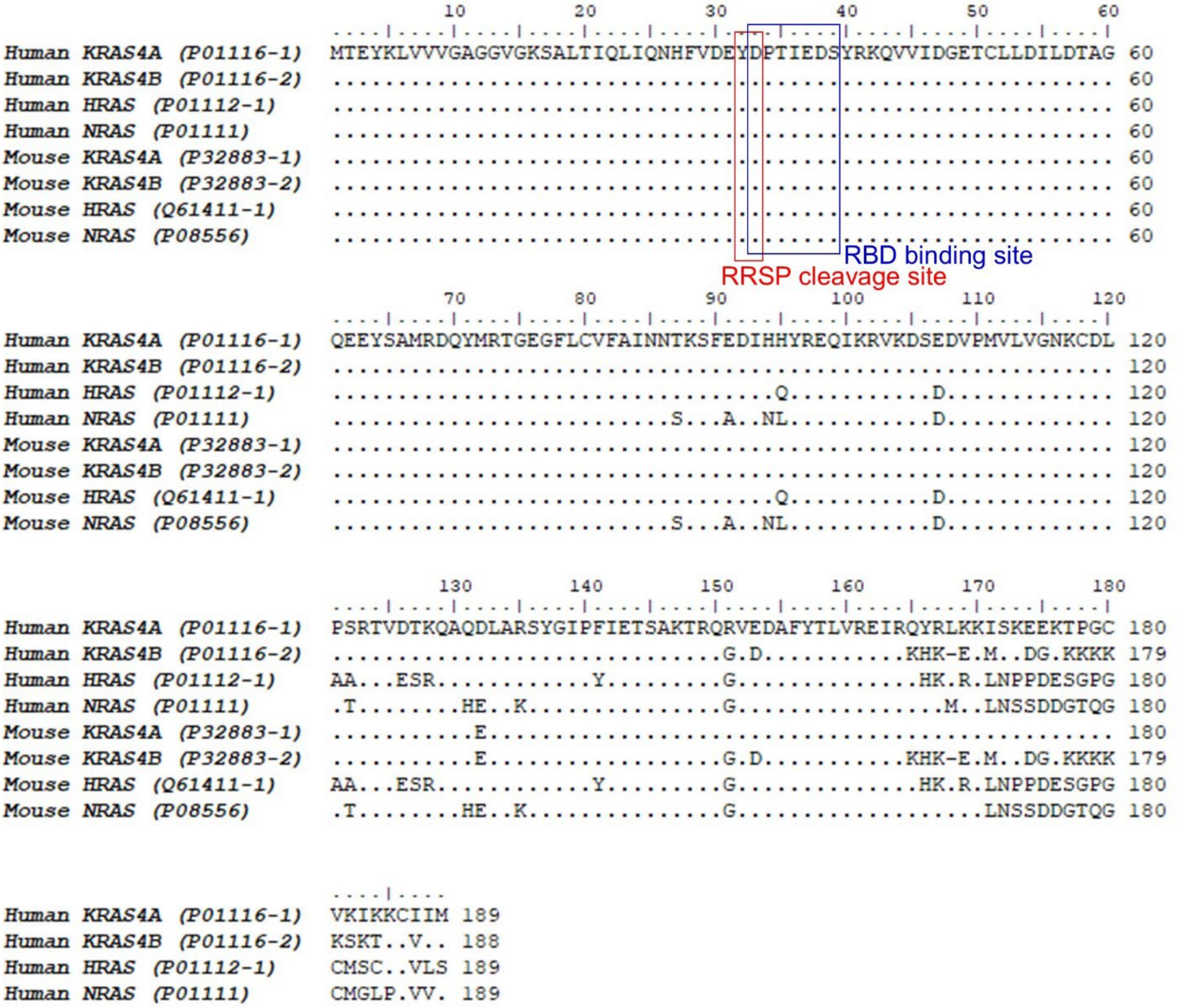

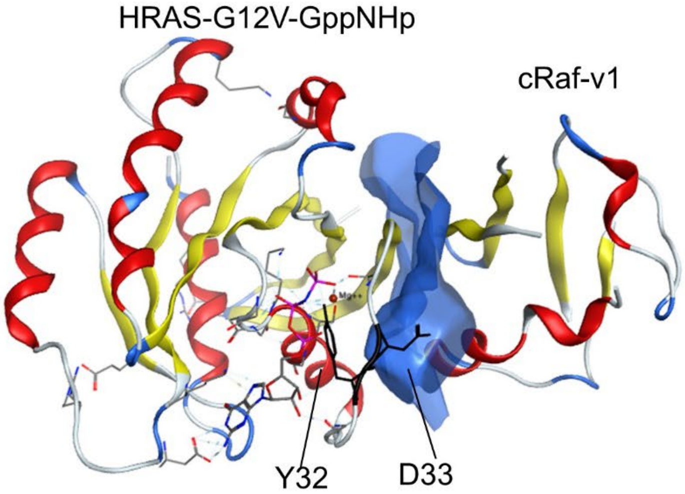

Critical Interactions (< −1 kcal/mol)

interaction	kcal/mol
KRAS(D38)-cRaf(R88)	−15.48
KRAS(S39)-cRaf(W67)	−5.53
KRAS(E37)-cRaf(V69)	−4.03
KRAS(D33)-cRaf(R88)	−1.88
KRAS(E37)-cRaf(T68)	−1.36
KRAS(S39)-cRaf(Q66)	−1.22
KRAS(D38)-cRaf(T68)	−1.03

Figure S6. Comparison of RAS amino acid sequences in humans vs. mice. Regions flanking the RRSP cleavage and RBD binding sites are fully conserved. The lower panel shows the 3D structure of HRAS-G12V-GppNHp in complex with cRaf-v1 RBD (PDB:6NTC), with key interacting side

chains analyzed via MOE's Contact Analysis. The RRSP cleavage site at D33 exhibits a minor interaction energy (−1.88 kcal/mol) with RBD.

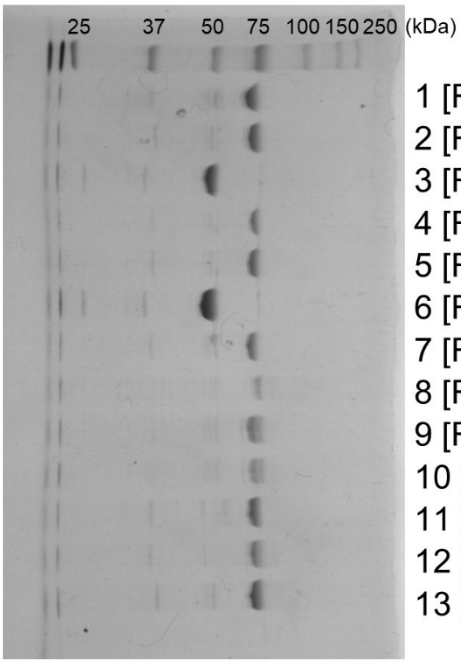

1 [RRSP]-[cRaf-v1]-GS12-[TAT]
2 [RRSP]-[cRaf-v1]-GS18-[TAT]
3 [RRSP]-GS15-[TAT]
4 [RRSP]-[cRaf-v1]-GS15-[TAT]
5 [RRSP]-[cRaf-v1]-GS15-[TAT] (Lot #2)
6 [RRSP]-GS15-[TAT] (Lot #2)
7 [RRSP]-[cRaf-v1]-[R8]
8 [RRSP]-[cRaf-v1]-[GET]
9 [RRSP]-[cRaf-v1]-[R10]
10 [RRSP]-[cRaf-v1]-[R12]
11 [Pen]-[RRSP]-[cRaf-v1]
12 [TAT]-[RRSP]-[cRaf-v1]
13 [RRSP]-[cRaf]-[TAT]

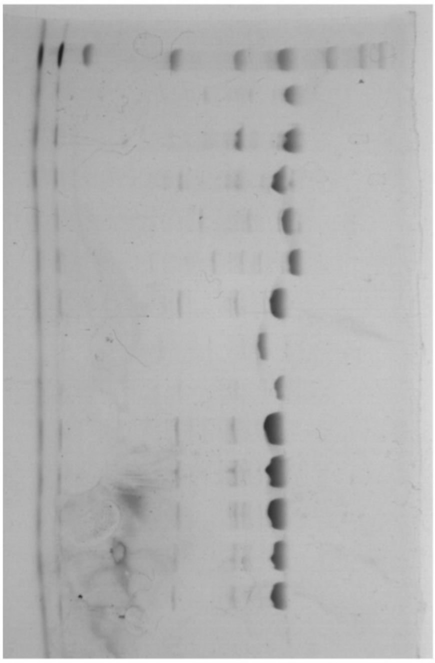

14 [RRSP]-[RASSF5]-[TAT]
15 [RRSP]-[K27]-[TAT]
16 [RRSP]-[RalGDS]-[TAT]
17 [RRSP]-[Afadin]-[TAT]
18 [RRSP]-[RIN1]-[TAT]
19 [RRSP]-[cRaf-v12]-[TAT]
20 [RRSP]-[225-3]-[TAT]
21 [RRSP]-[cRaf-v1]-[Pen]
22 [RRSP]-[cRaf-v1]-[TP10]
23 [RRSP]-[cRaf-v1]-[MAP]
24 [RRSP]-[cRaf-v1]-[CPP44]
25 [RRSP]-[cRaf-v1]-[CPP2]
26 [RRSP]-[cRaf-v1]-[PTD4]

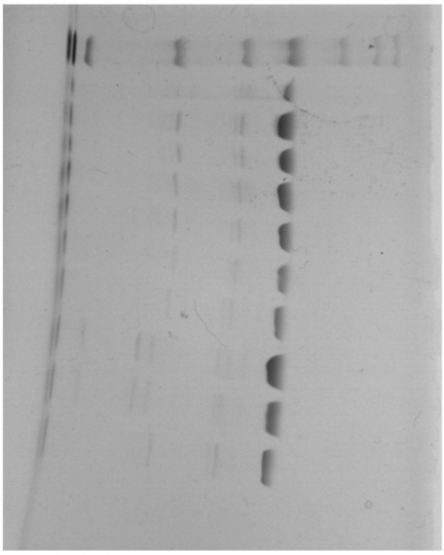

27 [RRSP]-[cRaf-v1]-[M918]
28 [RRSP]-[cRaf-v1(R88A/H89A)]-[TAT]
29 [RRSP]-[cRaf-v1]-GS15-[TAT] (Lot #3)
30 [RRSP]-[cRaf-v1]-[CPP44] (Lot #2)
31 [RRSP]-[cRaf-v1(R88A/H89A)]-[TAT] (Lot #2)
32 [RRSP]-[12VC1]-[TAT]
33 [RRSP]-[12VC1]-[TAT] (Lot #2)
34 [cRaf-v1]-[RRSP]-[TAT]
35 [cRaf-v1]-[RRSP]-[TAT] (Lot #2)
36 [cRaf-v1]-[JAM20]-[TAT]

Figure S7. CBB-stained SDS-PAGE images of 29 RRSP-RBD-CPPs (36 in total, including different production lots). Each lane was loaded with 1.6 μg of protein. In vitro EC50 values of each RSP-RBD-CPPs against CT-26 cancer cells (mouse KRAS-G12D mutant) are provided in Table S2.

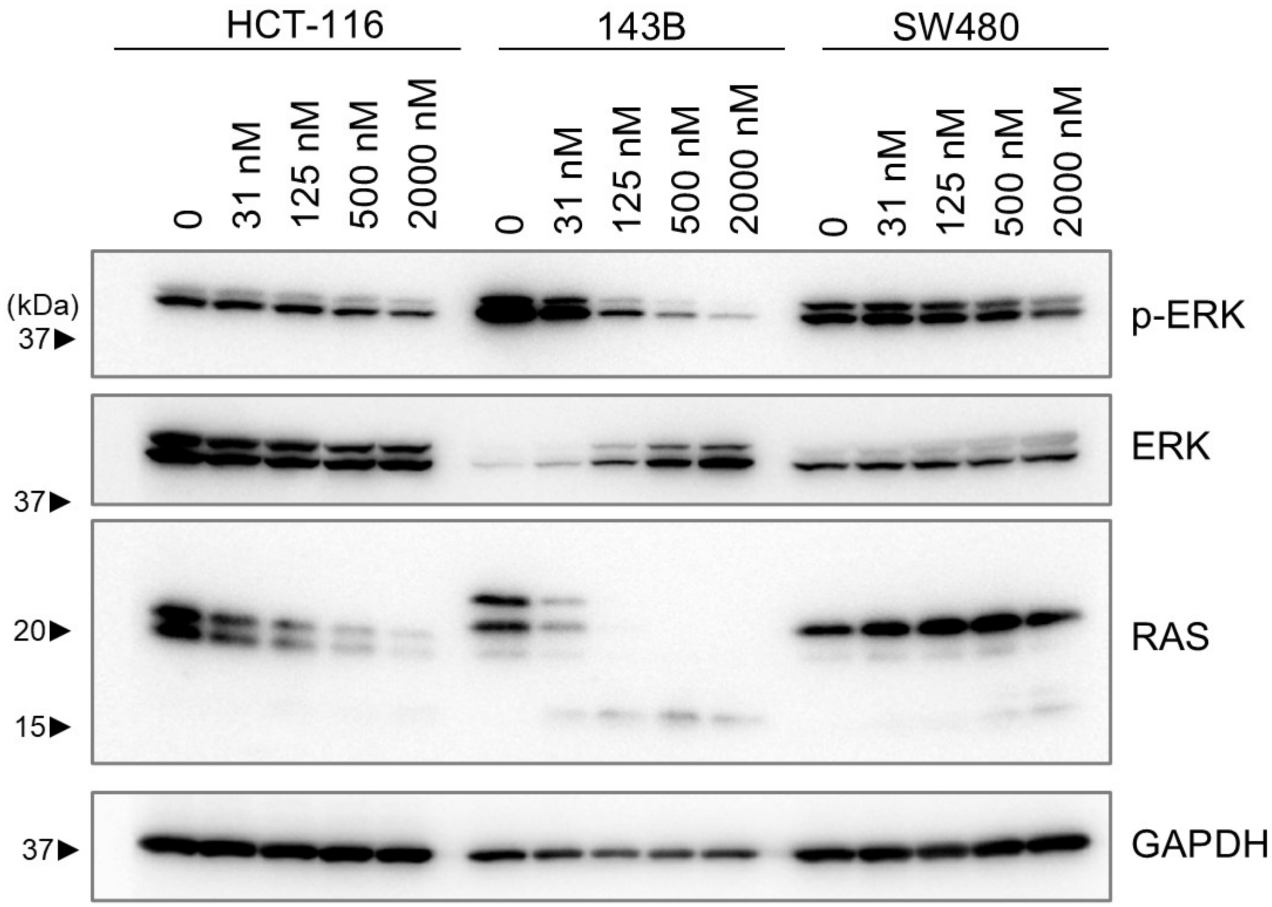

Figure S8. Immunoblot analysis of SW480, 143B, and HCT-116 cell lysates collected after 24 h treatment with RRSP-RBD-TAT at the indicated concentrations. For reference, the cell-viability EC_{50} values for HCT-116, 143B, and SW480 are > 2000 nM, 119 nM, and > 2000 nM, respectively (Supplementary Data 1). Representative immunoblots from two independent experiments with similar results. Full uncropped blots are provided in the Source Data file.

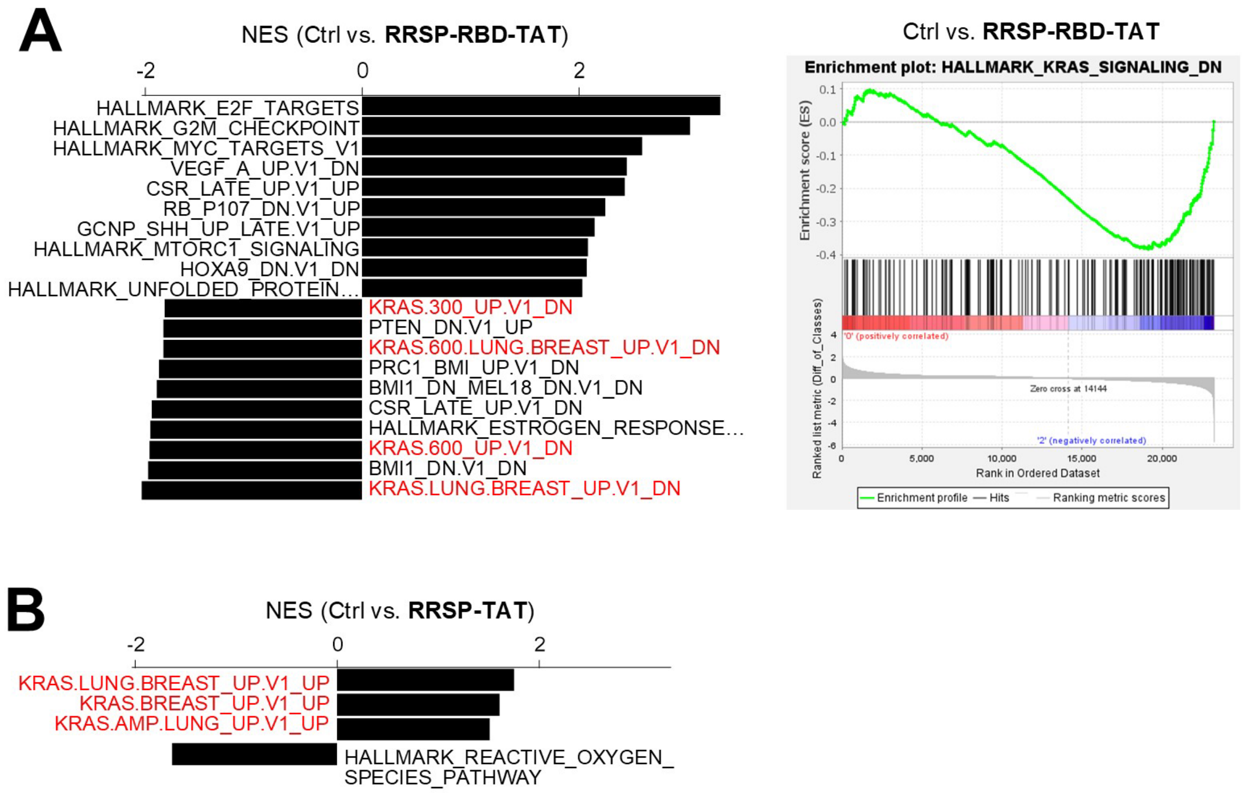

Figure S9. Microarray analysis of mRNA from PANC-1 cells treated with 250 nM RRSP-RBD-TAT or RRSP-TAT for 48 hours. (A) Gene set enrichment analysis (GSEA) reveals gene expression patterns in RRSP-RBD-TAT-treated cells similar to those observed under KRAS dominant-negative overexpression or KRAS pathway suppression. Additionally, alterations in cell cycle- and proliferation-related genes were observed. (B) RRSP-TAT treatment resulted in similar but weaker effects on gene expression.

Lot#	culture medium (L)	protein yield (mg)	mg/L	EC50 (nM)	Endotoxin (EU/mg)
1	20	116	5.8	163.5	13
2	20	134	6.7	126.8	3
3	20	65	3.2	117.4	4
4	20	207	10.3	133.2	0.9
5	20	139	7.0	117.2	0.4
6	20	72	3.6	102.6	0.2
7	20	203	10.2	121.5	1
8	10	110	11.0	106.8	5
av			7.2	123.6	3.4

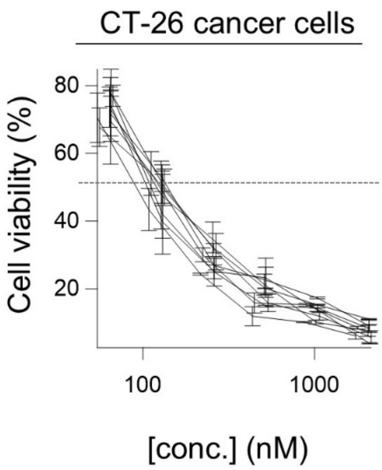

Figure S10. Batch-specific yield, EC50 values against CT-26 cells (mouse KRAS-G12D mutant), and endotoxin levels of RRSP-RBD-TAT used in mouse experiments. Right panel: Dose-response curves (cell viability vs. inhibitor concentration) for eight production lots, indicating consistent activity. Dose-response curves are shown as mean ± SD of n = 3 technical replicate wells.

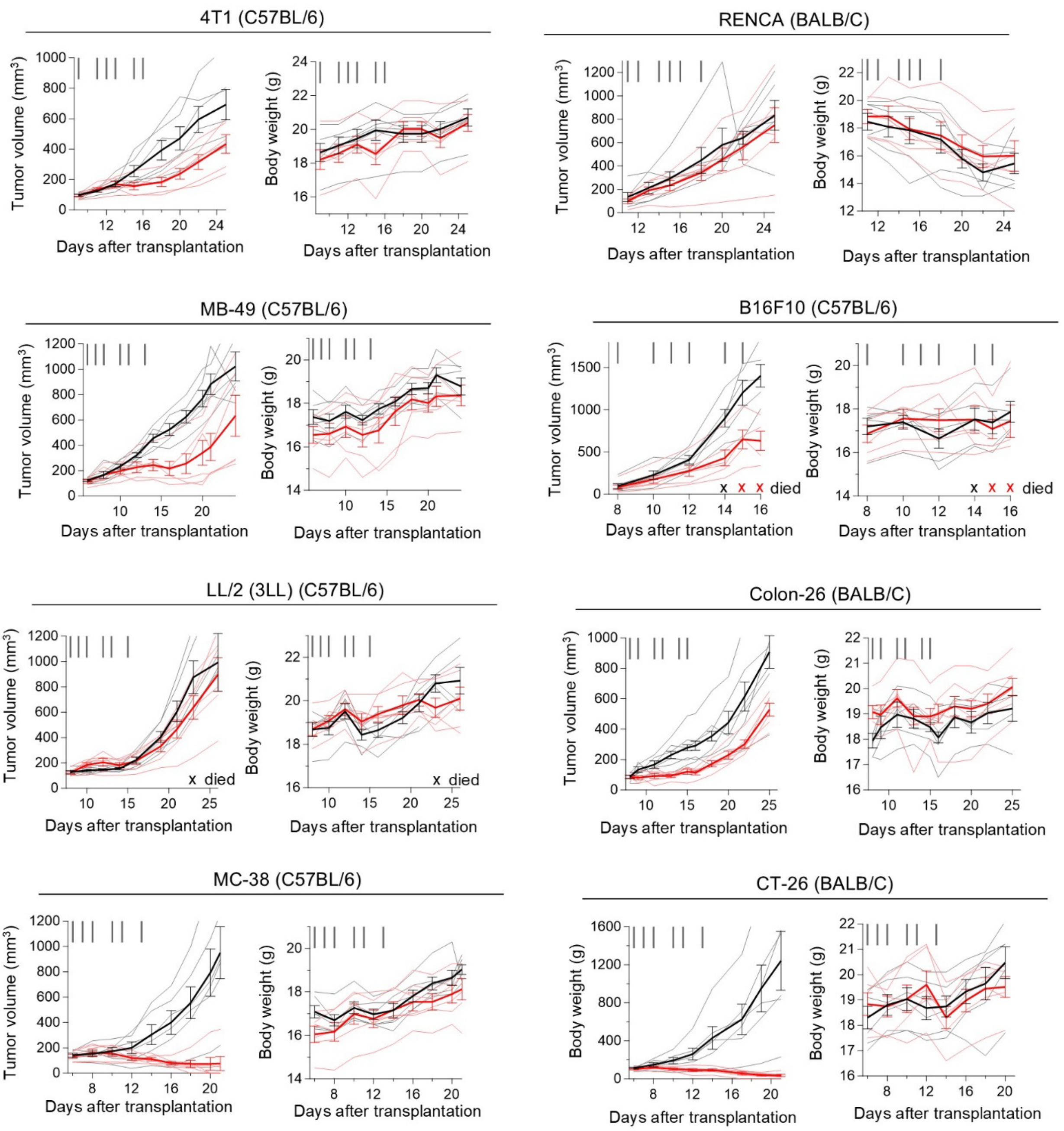
4T1 (C57BL/6)
RENCA (BALB/C)
MB-49 (C57BL/6)
B16F10 (C57BL/6)
LL/2 (3LL) (C57BL/6)
Colon-26 (BALB/C)
MC-38 (C57BL/6)
CT-26 (BALB/C)
Tumor volume (mm³)
Body weight (g)
Days after transplantation
x x x died
x died

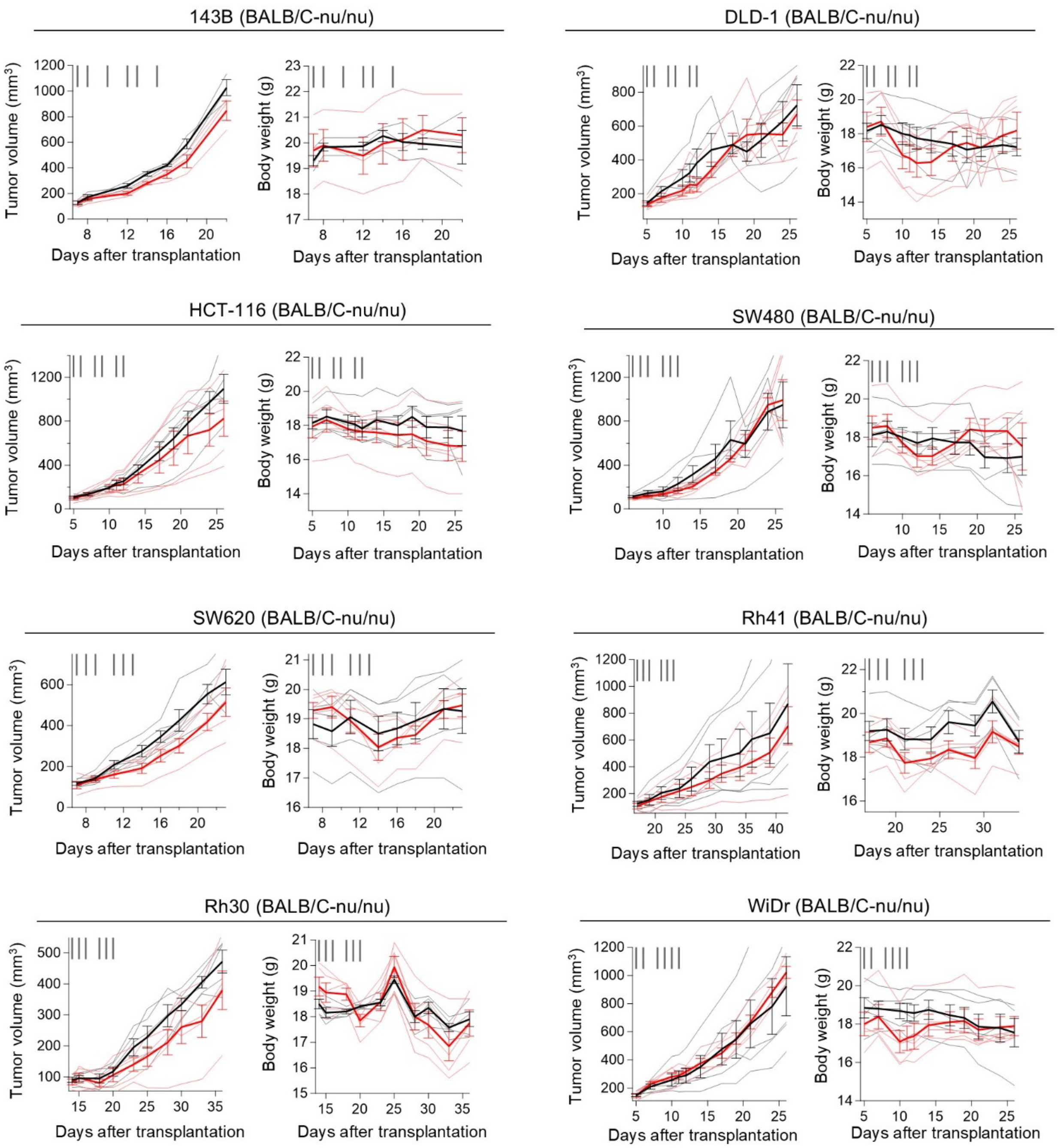

Figure S11. Tumor volume and body weight changes in 16 subcutaneous tumor-bearing mouse models treated with 50 mg/kg/day RRSP-RBD-TAT for six doses. Vertical lines indicate dosing days. Red lines represent the treatment group; the black lines represent the control group. Thin lines indicate individual mice; solid lines indicate mean ± SE. Each group included six mice for C57BL/6 and BALB/C models and five mice for BALB/C-nu/nu models. In the B16F10 and LL/2 models, three and one mice, respectively, died during the experiment. The solid lines represent mean ± SE.

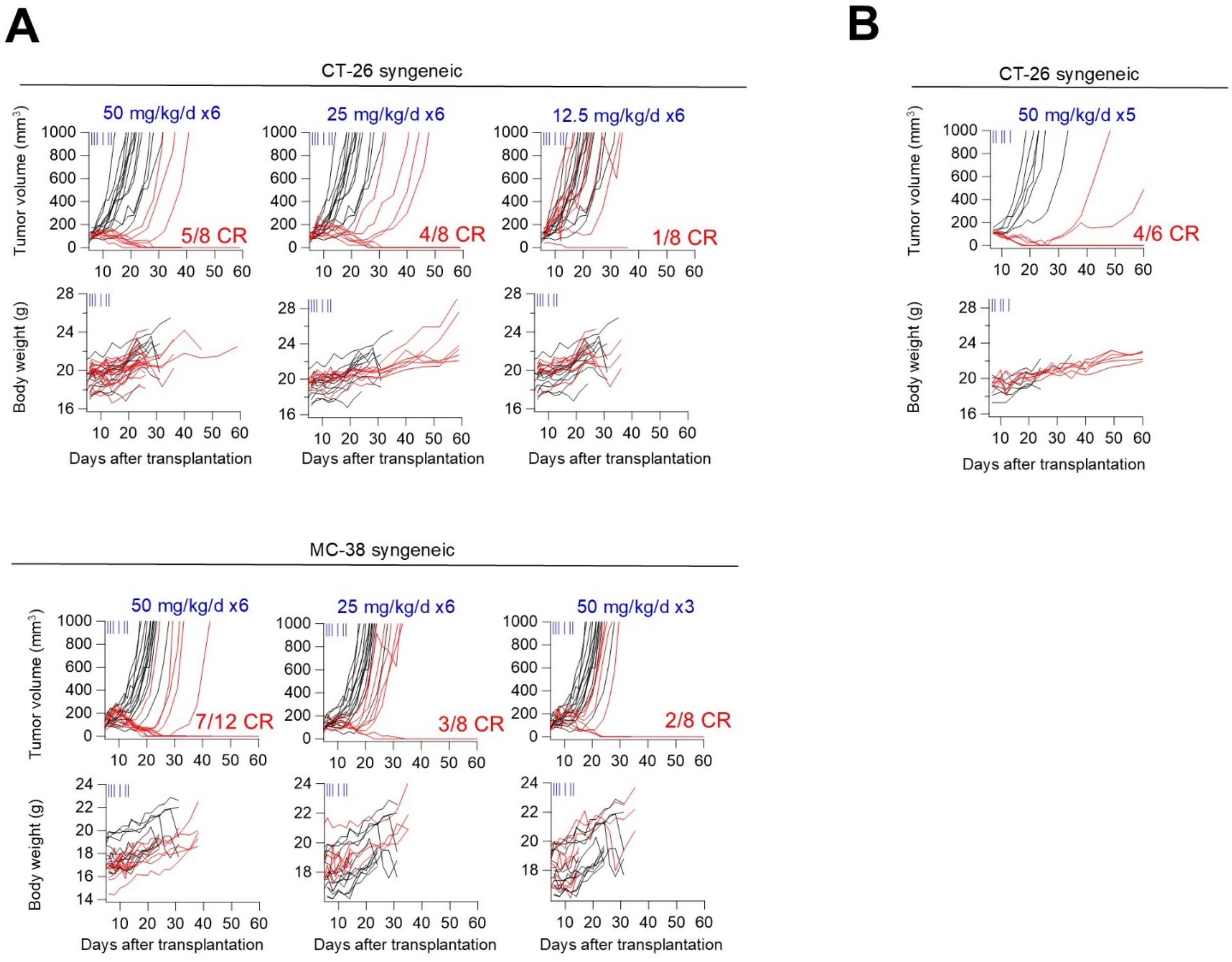

Figure S12. (A) Tumor volume and body-weight changes in CT-26 and MC-38 syngeneic subcutaneous models treated with RRSP-RBD-TAT under the indicated dosing regimens. Treatment began when the cohort mean tumor volume reached ~60 mm³. Vertical blue lines denote dosing days. CR indicates the number of mice achieving complete tumor regression. (B) Results from delayed-start cohorts in which treatment was initiated at a mean tumor volume of ~120 mm³.

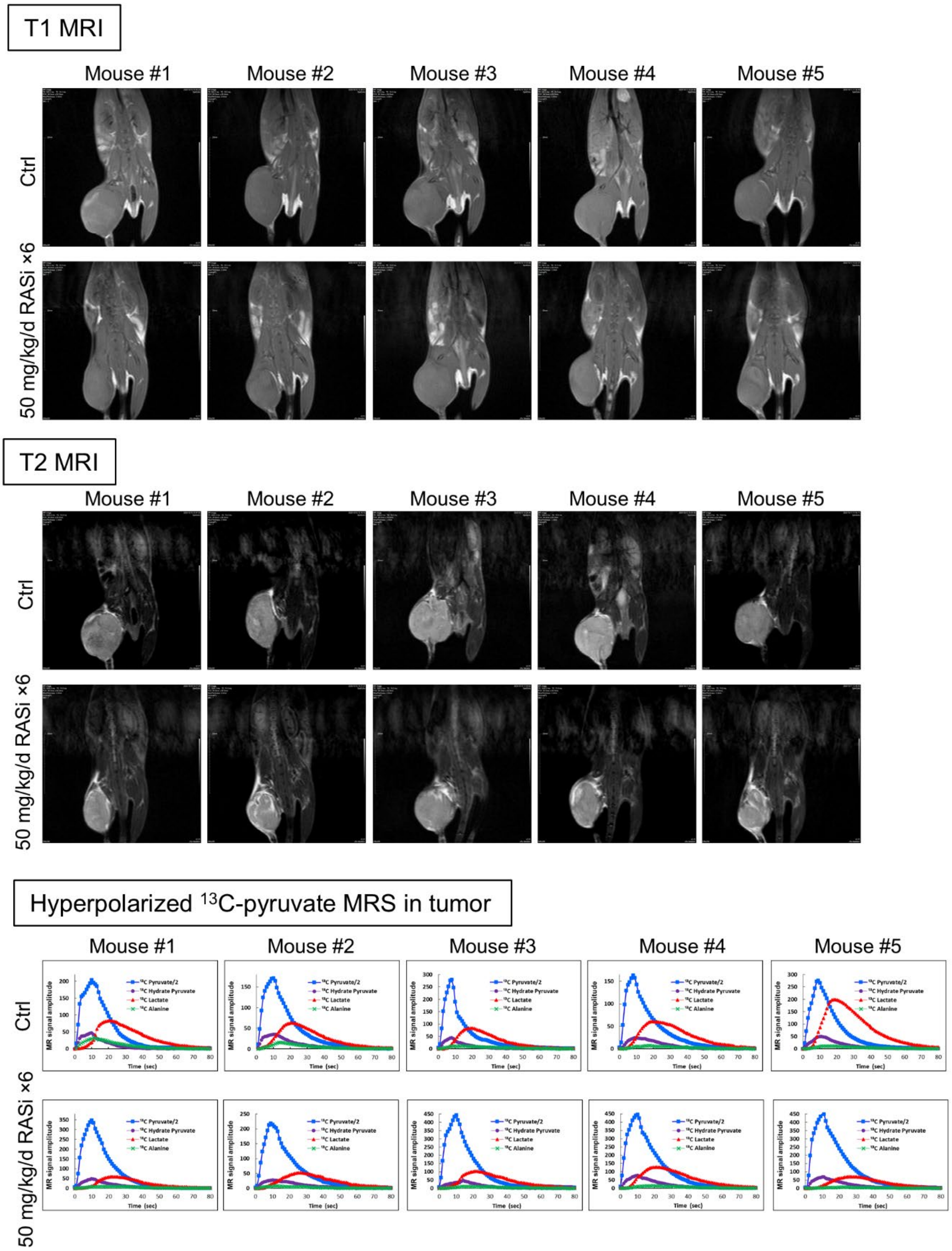

Figure S13. Representative T1-MRI, T2-MRI, and ^{13}C- hyperpolarization MRS measurements from n =5 mice per group in the CT-26 intramuscular model (BALB/C), 1–3 days after six doses of 50 mg/kg/day RRSP-RBD-TAT.

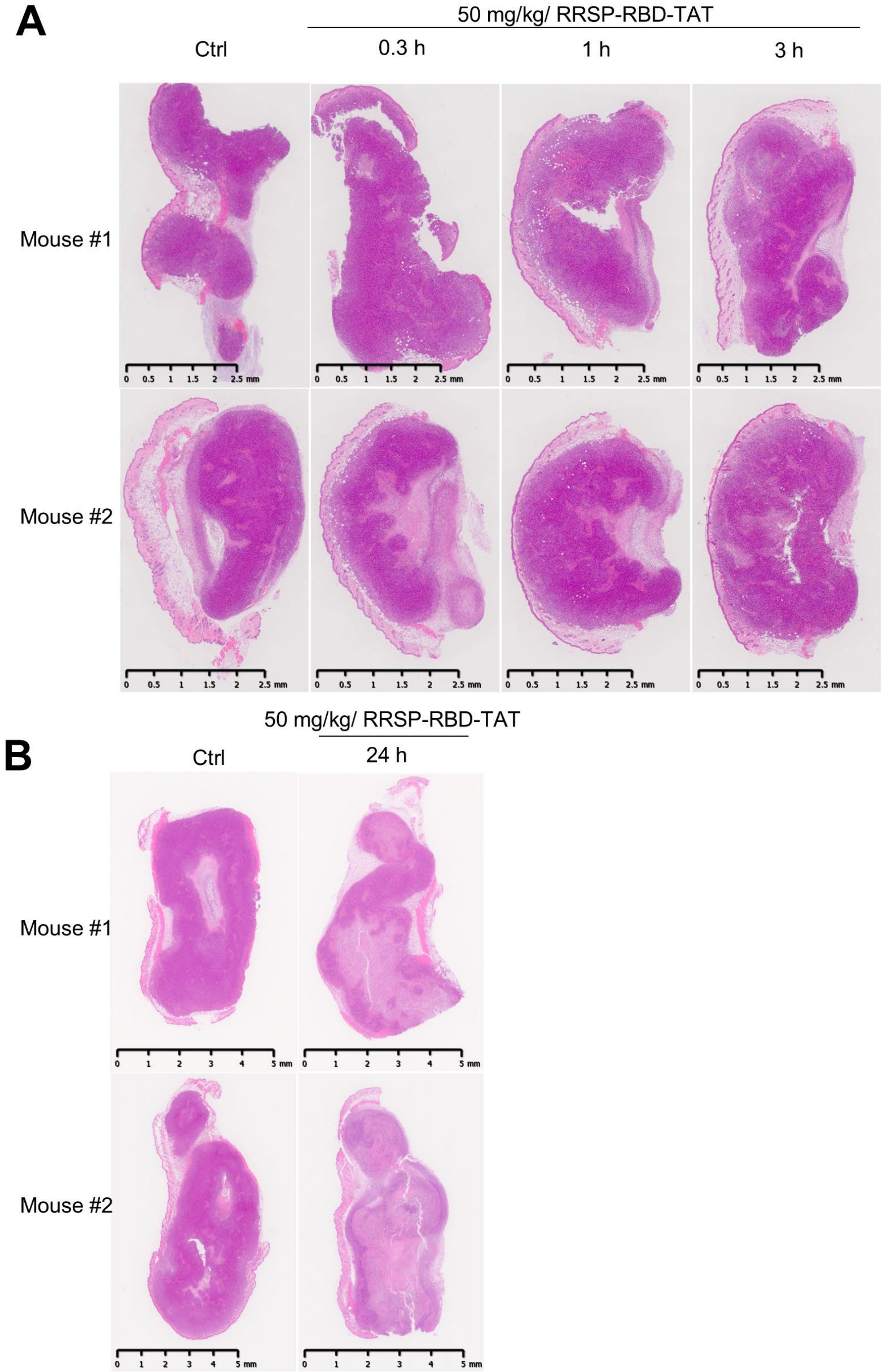
A
50 mg/kg/ RRSP-RBD-TAT
Ctrl
0.3 h
1 h
3 h
Mouse #1
Mouse #2
0 0.5 1 1.5 2 2.5 mm
50 mg/kg/ RRSP-RBD-TAT
B
Ctrl
24 h
Mouse #1
Mouse #2
0 1 2 3 4 5 mm

Figure S14. H&E-stained images of tumors from the Colon-26 subcutaneous model (BALB/C) excised 0.3–3 hours (A) and 24 hours (B) after a single 50 mg/kg dose of untagged or HA-tagged RRSP-RBD-TAT. Necrosis began at ~3 hours and covered 70–90% of the tumor area by 24 hours. Representative images from one experiment; similar findings were observed across the analyzed mice in the cohort.

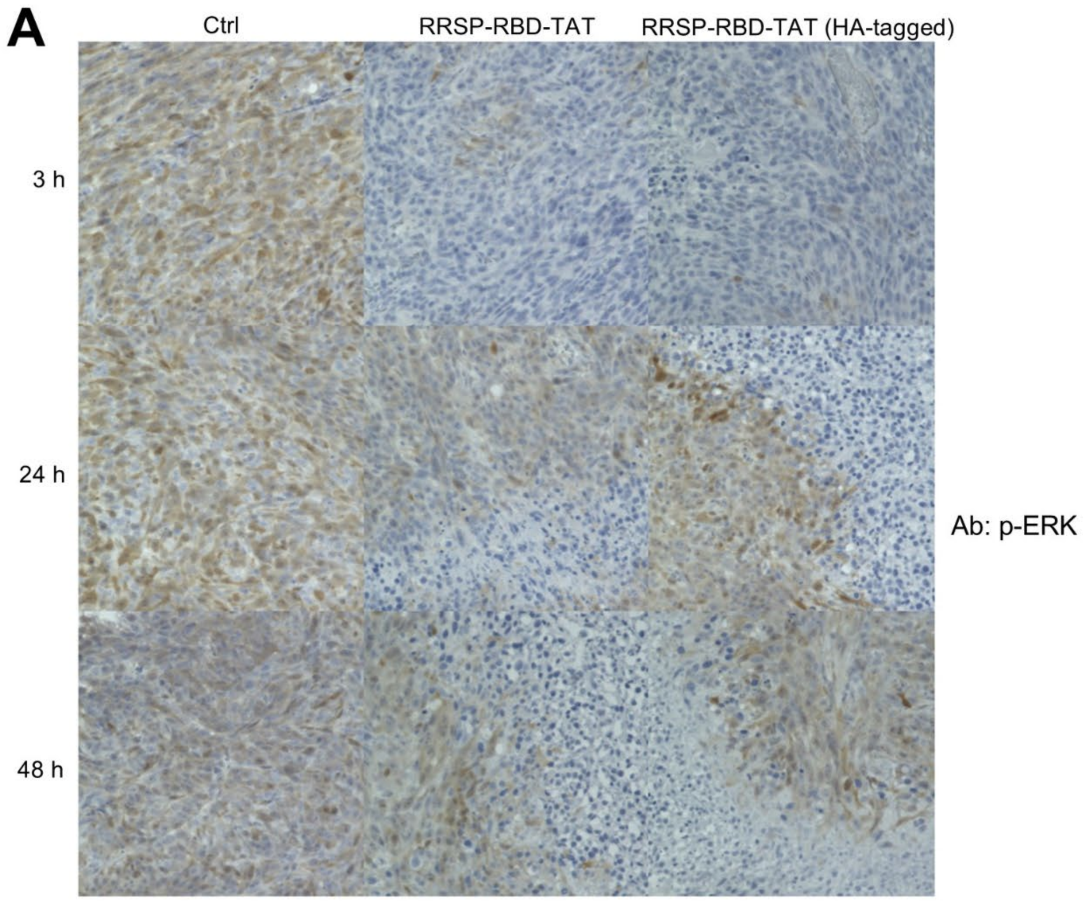
A
Ctrl
RRSP-RBD-TAT
RRSP-RBD-TAT (HA-tagged)
3 h
24 h
48 h
Ab: p-ERK

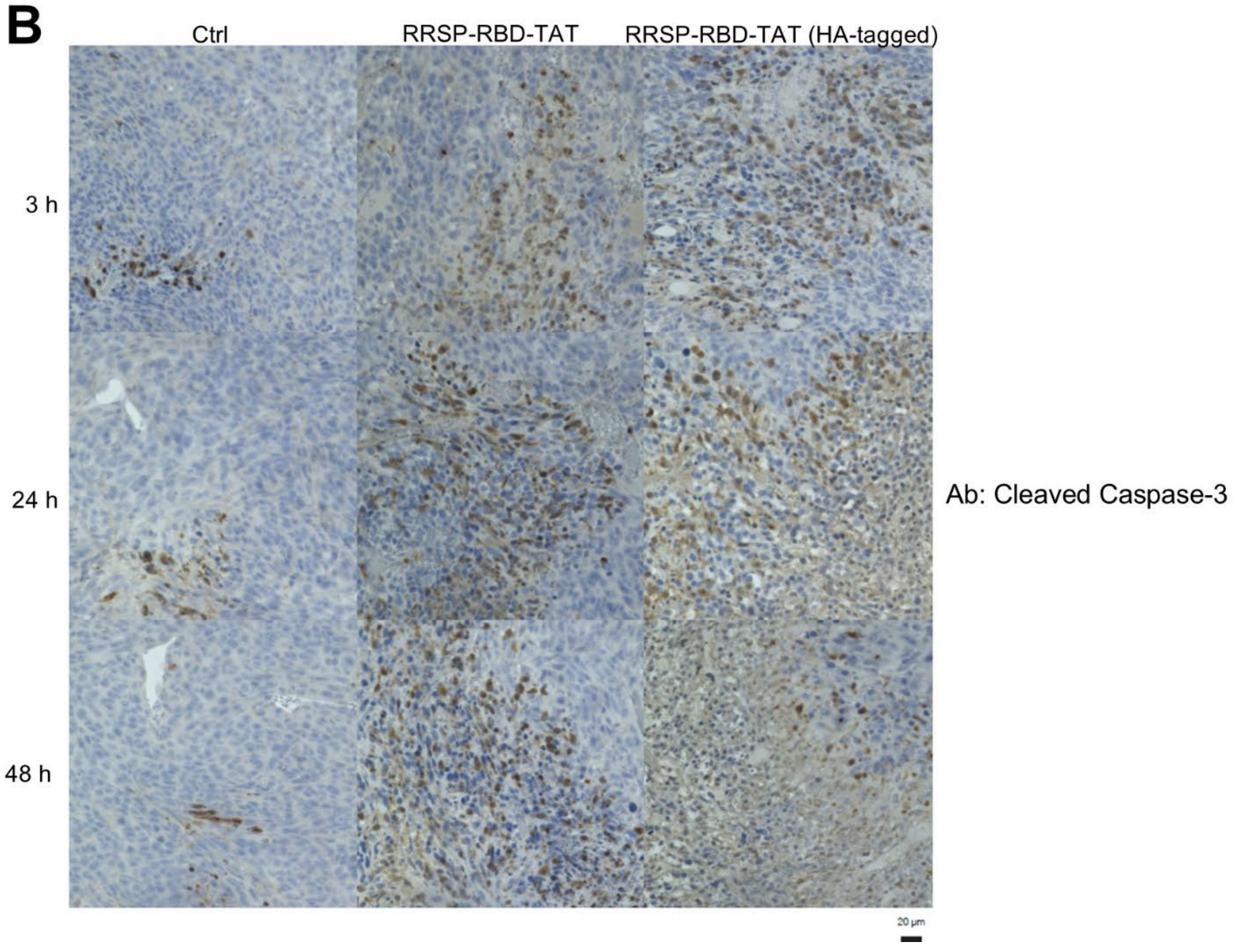
B
Ctrl
RRSP-RBD-TAT
RRSP-RBD-TAT (HA-tagged)
3 h
24 h
48 h
Ab: Cleaved Caspase-3
20 µm

Figure S15. Immunohistochemical images of tumors from the Colon-26 subcutaneous model (BALB/C) treated with a single 50 mg/kg dose of untagged or HA-tagged RRSP-RBD-TAT, excised 3–48 hours post-administration. Representative images from one experiment; similar findings were observed across the analyzed mice in the cohort.

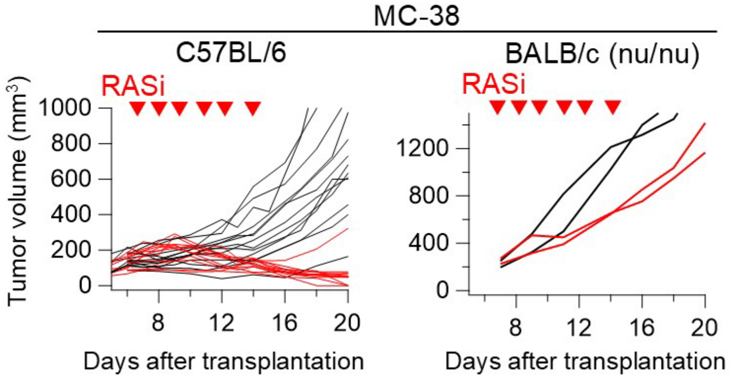

Figure S16. Tumor volume changes in MC-38 subcutaneous models transplanted into immunocompetent C57BL/6 vs. immunodeficient BALB/C-nu/nu mice, treated with 50 mg/kg/day RRSP-RBD-TAT for 6 doses. RRSP-RBD-TAT was less effective in the immunodeficient model, similar to Colon-26 models (Figure 4A).

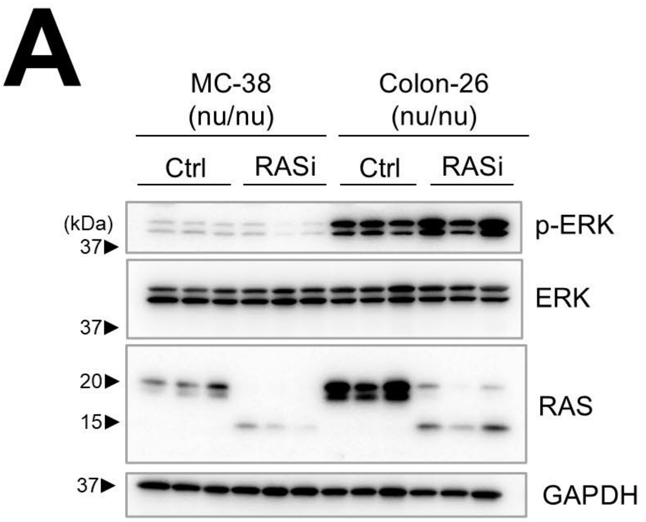

A
MC-38
(nu/nu)
Colon-26
(nu/nu)
Ctrl
RASi
Ctrl
RASi
(kDa)
37▶
p-ERK
ERK
37▶
20▶
15▶
RAS
37▶
GAPDH

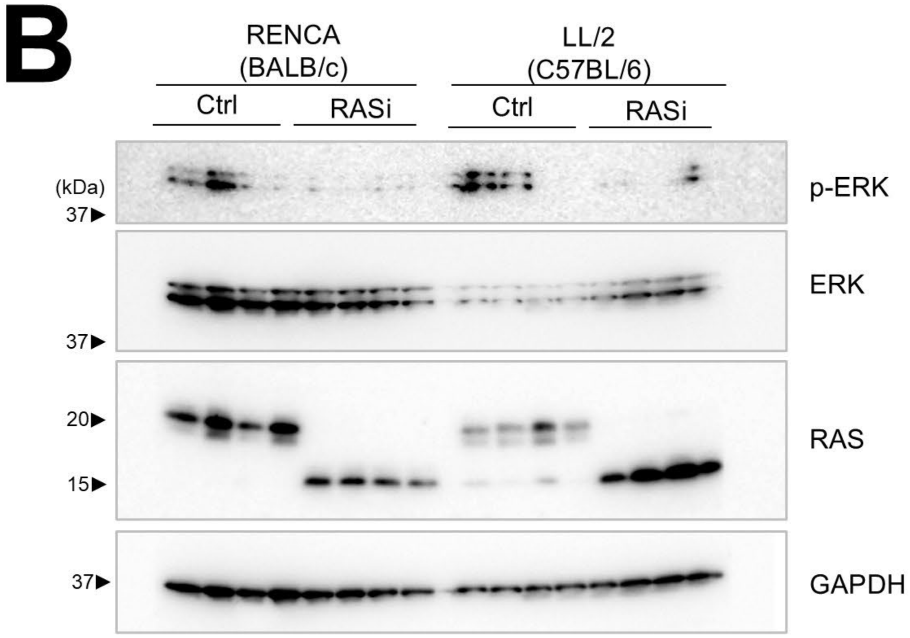

B
RENCA
(BALB/c)
LL/2
(C57BL/6)
Ctrl
RASi
Ctrl
RASi
(kDa)
37▶
p-ERK
ERK
37▶
20▶
15▶
RAS
37▶
GAPDH

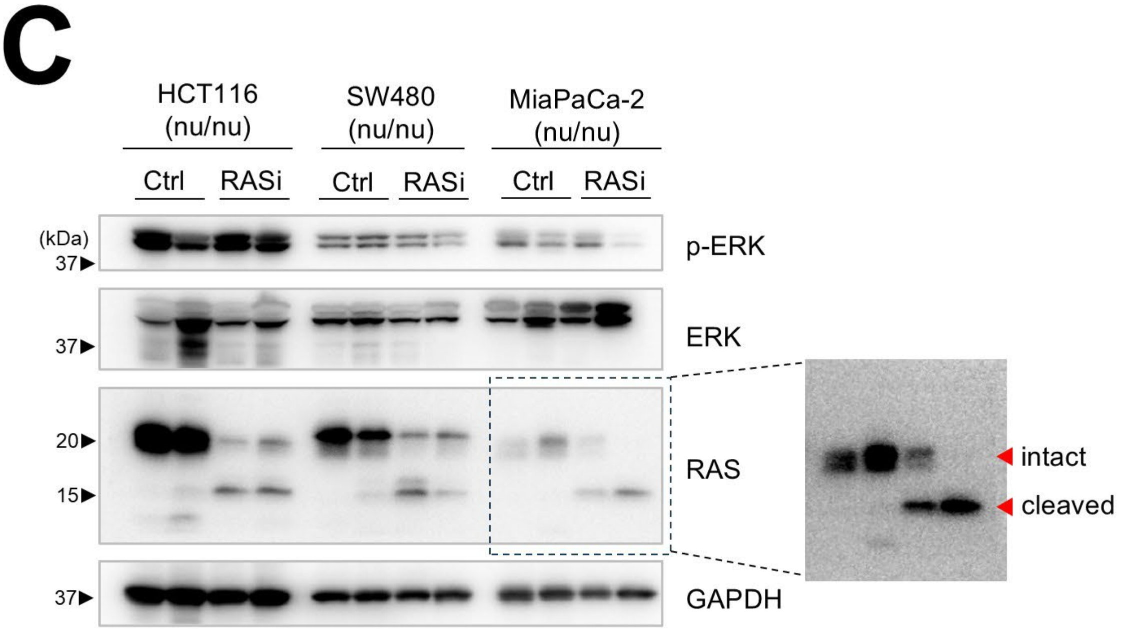

C
HCT116
(nu/nu)
SW480
(nu/nu)
MiaPaCa-2
(nu/nu)
Ctrl
RASi
Ctrl
RASi
Ctrl
RASi
(kDa)
37▶
p-ERK
37▶
ERK
20▶
15▶
RAS
intact
cleaved
37▶
GAPDH

Figure S17. Immunoblot analysis of tumor lysates collected 3 h after a single 50 mg/kg i.v. dose of RRSP-RBD-TAT (RASi) in: (A) MC-38 nu/nu and Colon-26 nu/nu mice; (B) RENCA and LL/2 syngeneic mice; and (C) SW480, 143B, and HCT-116 human xenografts in nu/nu mice. Panel A is an independent biological replicate of the experiment shown in Figure 4C. Representative immunoblots from two independent experiments with similar results. Full uncropped blots are provided in the Source Data file.

	Colon-26 syngeneic					MC-38 syngeneic	
		50 mg/kg/d RASi ×1	50 mg/kg/d RASi ×1	50 mg/kg/d RASi ×2	50 mg/kg/d RASi ×5		50 mg/kg/d RASi ×3
Times after the first administration (h)	0	3	24	72	168	0	108
CD3+T cell	1	0.5	0.2	0.0	0.1	1	1.9
CD4+T cell	1	0.4	0.2	0.0	0.1	1	2.4
Treg	1	0.5	0.1	0.0	0.1	1	1.6
CD8+T cell	1	0.5	0.1	0.0	0.0	1	1.6
B cell	1	0.5	0.2	0.1	0.3	1	0.5
monocytic MDSC	1	0.8	0.1	0.1	0.1	1	0.7
granulocytic MDSC	1	2.3	0.4	2.4	10	1	2.5
monocyte	1	2.9	0.3	0.5	0.8	1	1.6
macrophage	1	3.3	0.2	0.3	0.2	1	1.2
M1 macrophage	1	3.3	0.3	0.1	0.0	1	0.7
M2 macrophage	1	1.2	0.1	0.0	0.1	1	1.9
dendritic cell (DC)	1	1.0	0.0	0.0	0.0	1	0.1
natural killer (NK)	1	0.2	0.1	0.0	0.0	1	0.8
NK T cells	1	0.4	0.2	0.0	0.0	1	1.2

(fold change vs. 0 h)

Figure S18. Fold changes in tumor-infiltrating lymphocyte (TIL) counts in Colon-26 and MC-38 syngeneic tumors after RRSP-RBD-TAT treatment at the indicated times. Values indicate mean fold changes relative to 0 h, calculated from n = 4 tumors (one tumor per mouse) per time point. The color scale corresponds to the fold-change values shown in each cell. TIL counts decreased from 3–24 h in the Colon-26 model.

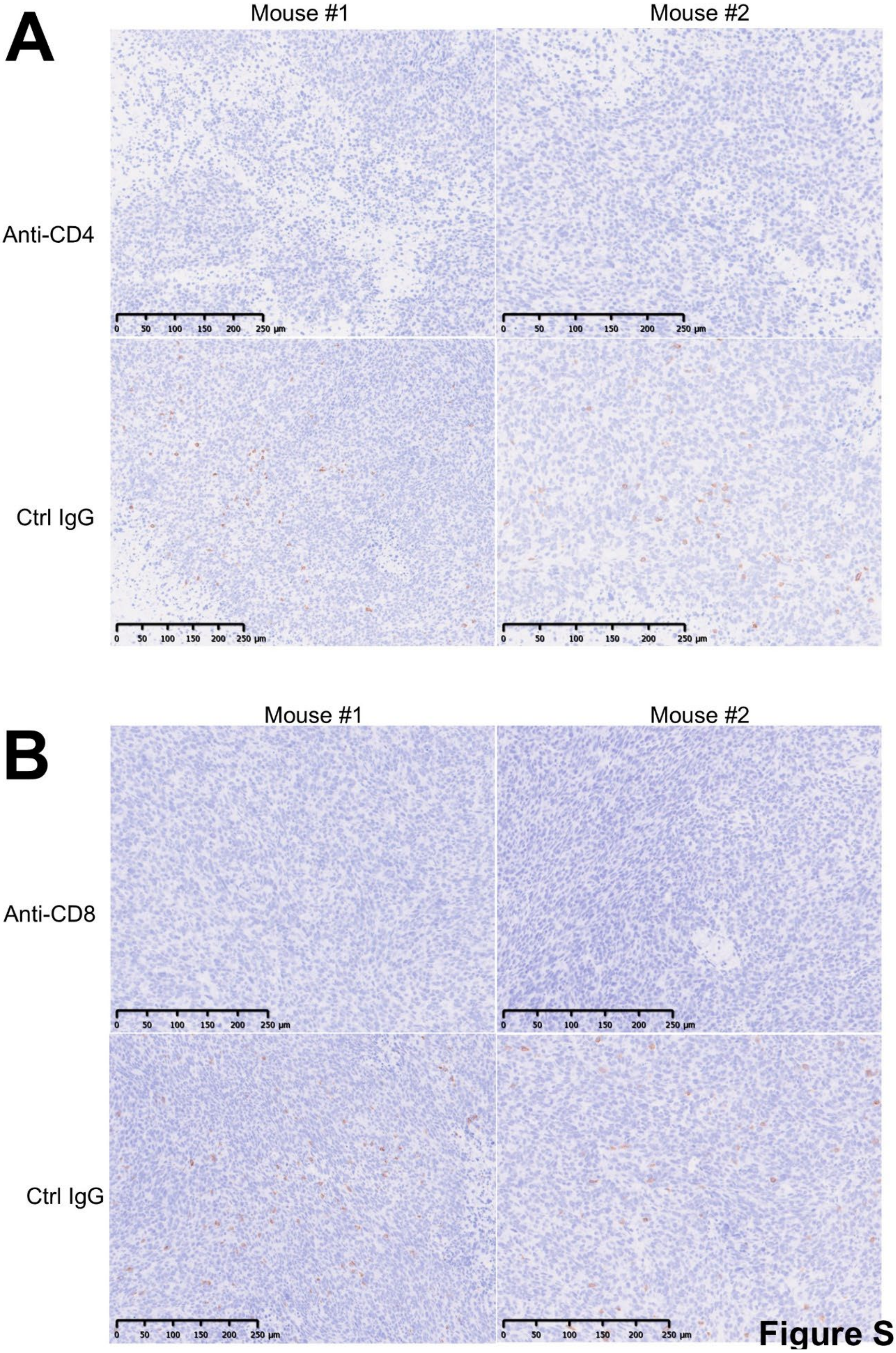
A
Mouse #1
Mouse #2
Anti-CD4
Ctrl IgG
0 50 100 150 200 250 µm
B
Mouse #1
Mouse #2
Anti-CD8
Ctrl IgG
0 50 100 150 200 250 µm

Figure S19-1

C

	Ctrl IgG	Anti-Asialo GM1	Anti-CD4	Anti-CD8	Ctrl IgG + 50 mg/kg/d RASi × 2
CD3+T cell	26	23	18	16	29
CD4+T cell	17	17	0	14	20
Treg	3	3	0	2	3
CD8+T cell	6	4	13	0	6
B cell	49	60	53	62	37
mMDSC	2	2	3	2	1
gMDSC	3	5	3	3	12
monocyte	6	1	8	5	5
macrophage	0	1	1	1	1
M1	0	0	0	0	0
M2	0	0	0	0	1
DC	0	0	0	0	0
NK	8	0	11	7	6
NKT	4	3	3	2	3

(% of CD45+ cells in spleen)

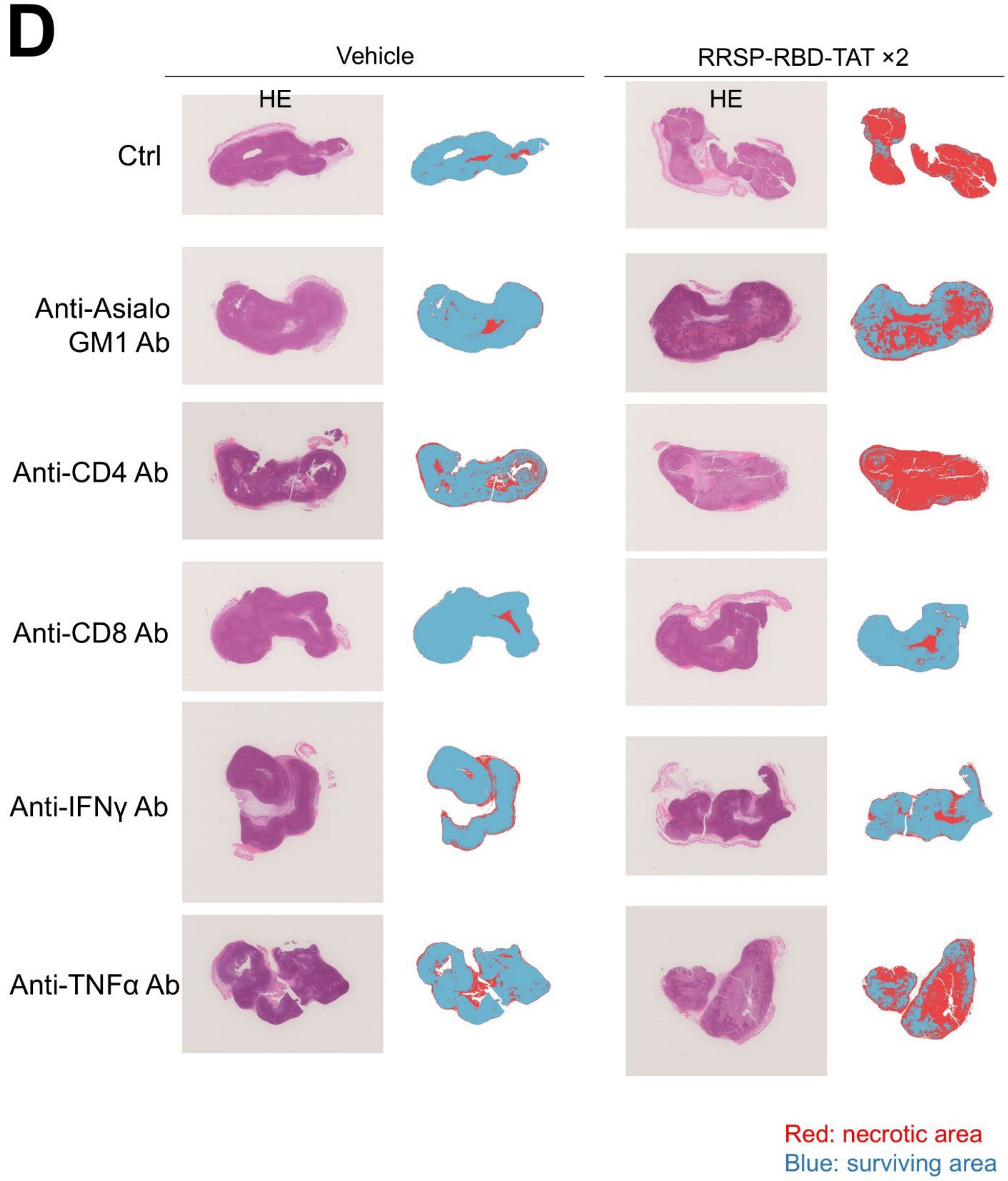

Figure S19. Validation of immune cell depletion in the experiment shown in Figure 4G. (A) CD4 IHC staining of tumors from the Colon-26 model (BALB/C) after two anti-CD4 antibody doses. (B) CD8 IHC staining of tumors after two anti-CD8 antibody doses. (C) Immune cell counts in the spleens of mice treated with anti-CD4, anti-CD8, or anti-Asialo-GM1 antibodies. Values indicate the percentage of each immune-cell population among CD45+ splenocytes, shown as mean

values from n = 3 mice per group. (D) Representative H&E-stained tumor sections, showing necrotic (red) and viable (blue) regions. Representative images from one experiment; similar findings were observed across the analyzed mice in the cohort.

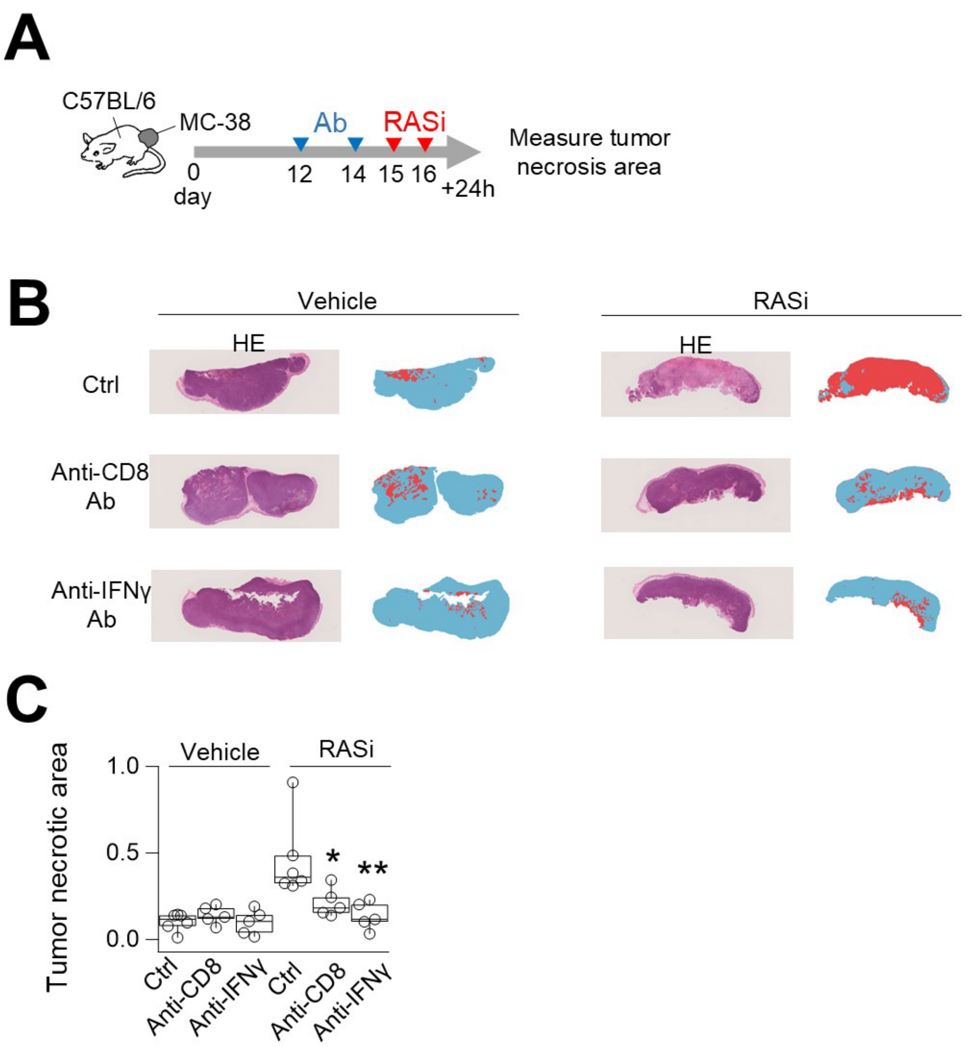

Figure S20. MC-38 CD8/IFNγ depletion assay. (A) Schematic of the dosing protocol. MC-38 cells were transplanted into immunocompetent C57BL/6 mice; anti-CD8 antibody (7.5 mg/kg, i.p.) and anti-IFNγ antibody (10 mg/kg, i.p.) were administered on days 12 and 14 post-inoculation, followed by RRSP-RBD-TAT (RASi; 125 mg/kg, i.v.) on days 16 and 17. Tumors were collected 24 h after the final RASi dose for histology. (B) Representative H&E-stained tumor sections, with necrotic regions (red) and viable regions (blue) indicated. (C) Quantification of tumor necrotic area. Data are shown as box plots with median, interquartile range, and whiskers showing min–max. P values were calculated by one-way ANOVA followed by Tukey's multiple-comparisons test. Exact P values are provided in Supplementary Data 4.

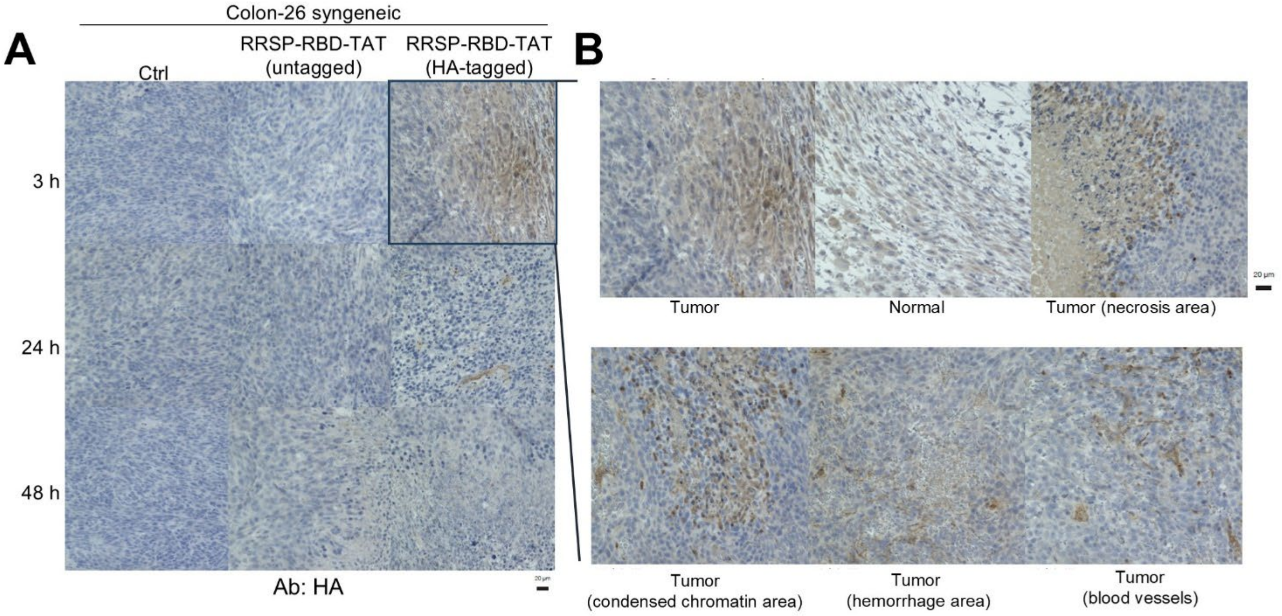

Figure S21. (A) Anti-HA IHC images of tumors from the Colon-26 subcutaneous model (BALB/C) treated with a single 50 mg/kg dose of HA-tagged or untagged RRSP-RBD-TAT, excised 3–48 hours after treatment. (B) Higher magnification images of figure S21A, showing drug delivery to both tumor and normal cells. Representative images from one experiment; similar findings were observed across the analyzed mice in the cohort.

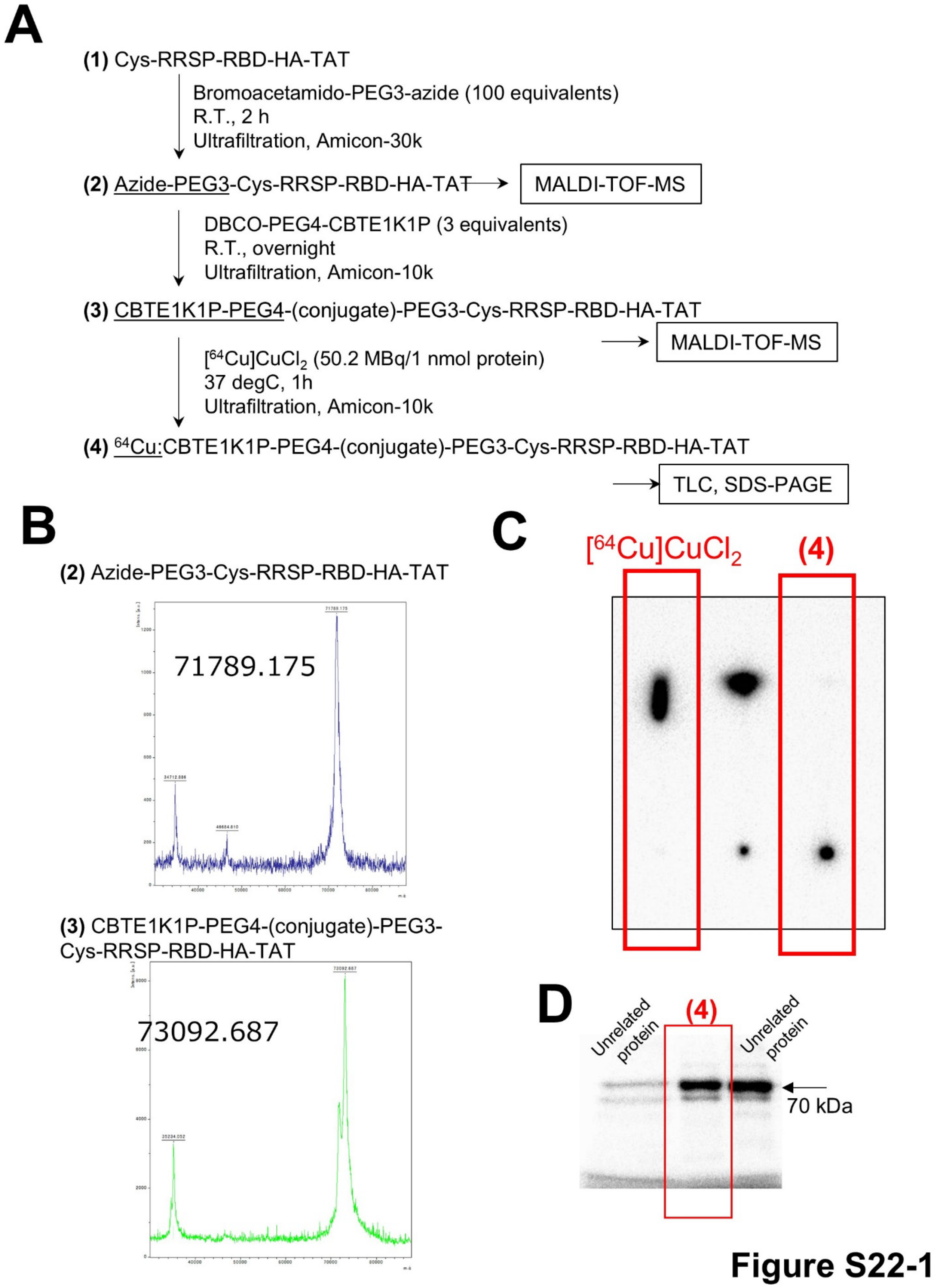
A
(1) Cys-RRSP-RBD-HA-TAT
Bromoacetamido-PEG3-azide (100 equivalents)
R.T., 2 h
Ultrafiltration, Amicon-30k
(2) Azide-PEG3-Cys-RRSP-RBD-HA-TAT
MALDI-TOF-MS
DBCO-PEG4-CBTE1K1P (3 equivalents)
R.T., overnight
Ultrafiltration, Amicon-10k
(3) CBTE1K1P-PEG4-(conjugate)-PEG3-Cys-RRSP-RBD-HA-TAT
MALDI-TOF-MS
[64Cu]CuCl2 (50.2 MBq/1 nmol protein)
37 degC, 1h
Ultrafiltration, Amicon-10k
(4) 64Cu:CBTE1K1P-PEG4-(conjugate)-PEG3-Cys-RRSP-RBD-HA-TAT
TLC, SDS-PAGE
B
(2) Azide-PEG3-Cys-RRSP-RBD-HA-TAT
71789.175
(3) CBTE1K1P-PEG4-(conjugate)-PEG3-Cys-RRSP-RBD-HA-TAT
73092.687
C
[64Cu]CuCl2
(4)
D
Unrelated protein
(4)
Unrelated protein
70 kDa

Figure S22-1

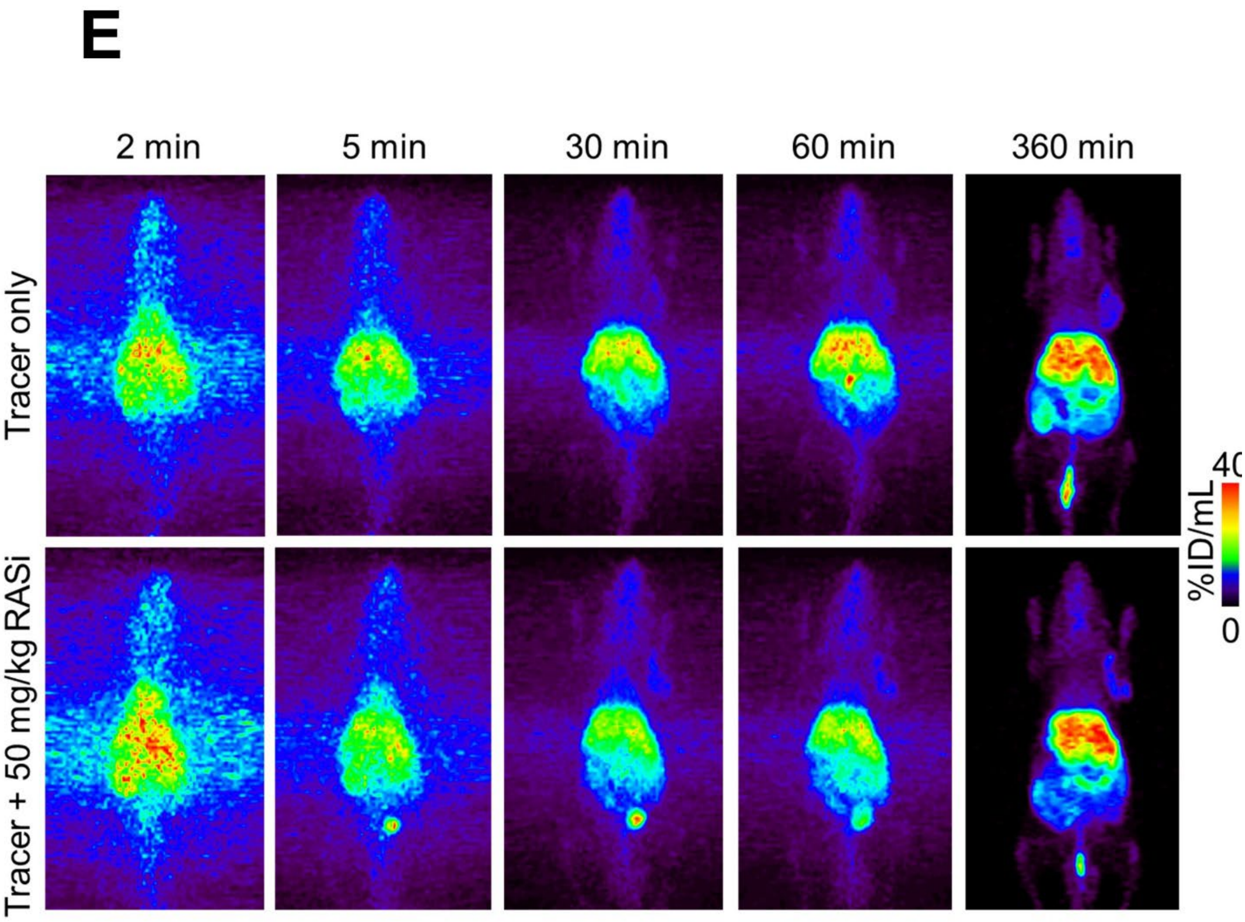

Figure S22. (A) Synthetic pathway for the preparation of the ^{64}Cu-RASi used in PET measurements. (B) MALDI-TOF-MS analysis of reaction intermediates. (C) Radio-TLC analysis of the final purified product, showing a radiochemical purity of approximately 91%. (D) Radio-SDS-PAGE image of the final purified product. (E) Additional PET images obtained from the experiments described in Figure 5C. Representative images from one experiment; similar findings were observed across the analyzed mice in the cohort.

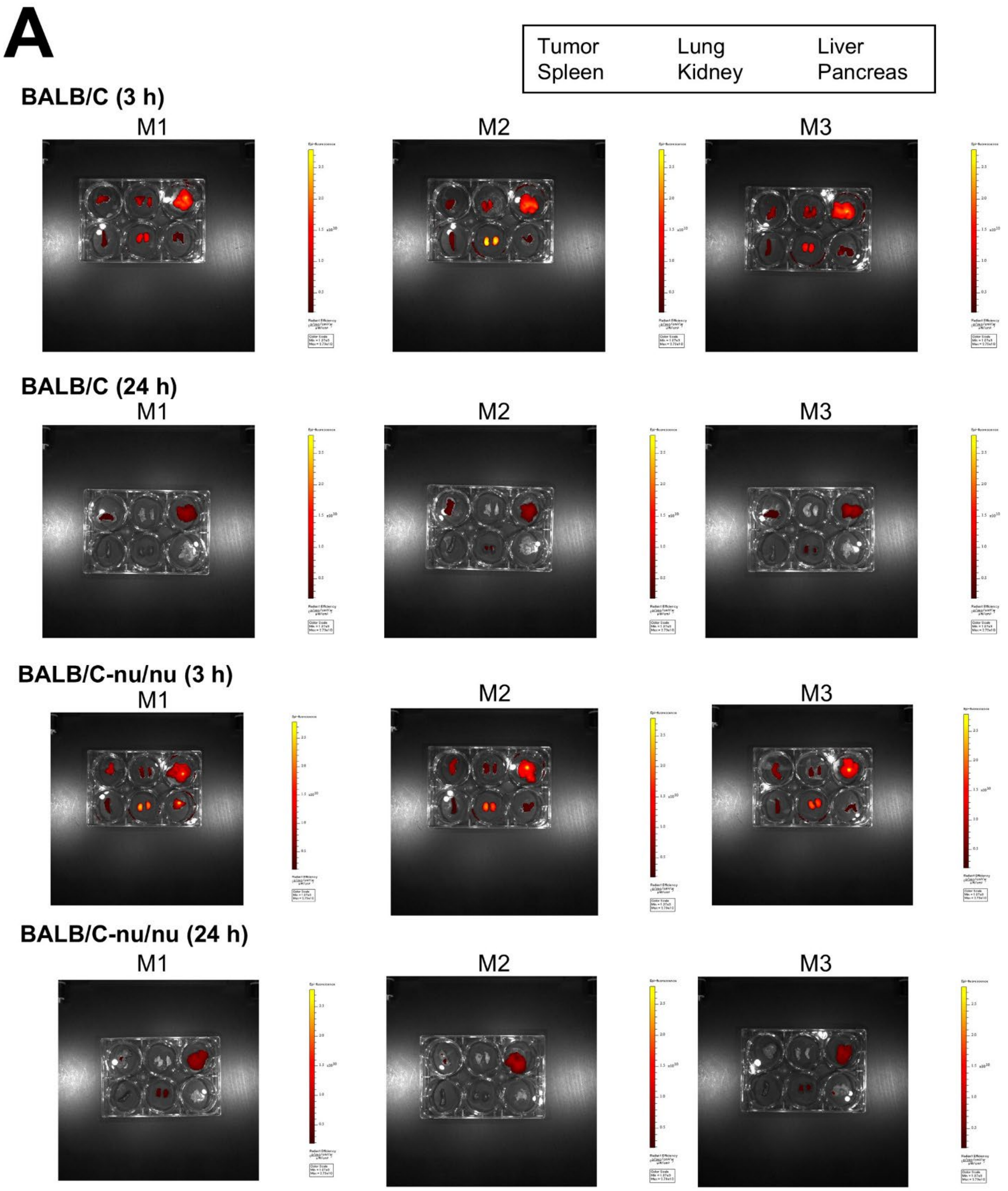
A
Tumor
Lung
Liver
Spleen
Kidney
Pancreas
BALB/C (3 h)
M1
M2
M3
BALB/C (24 h)
M1
M2
M3
BALB/C-nu/nu (3 h)
M1
M2
M3
BALB/C-nu/nu (24 h)
M1
M2
M3

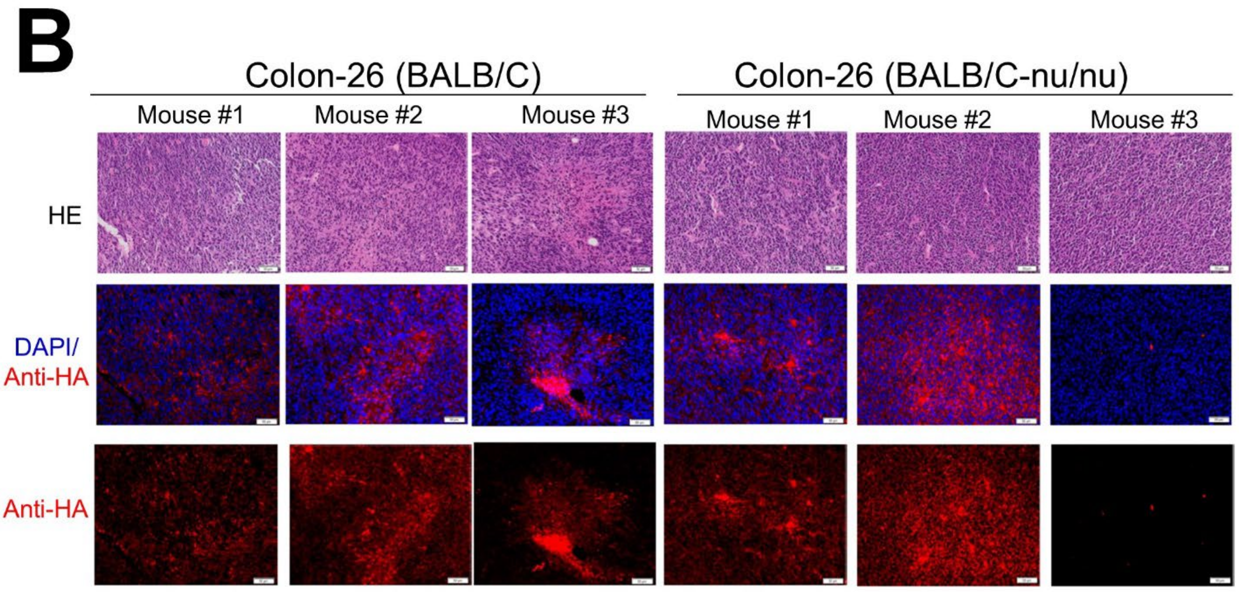

Figure S23. Fluorescence images from the experiments in Figure 5E and F. (A) Ex vivo fluorescence imaging of multiple tissues at 3 and 24 hours after administration. (B) H&E-, anti-HA- and DAPI-stained images of tumors 3 hours after treatment. No clear differences in drug distribution were observed between the BALB/C and nu/nu models.

Findings	Vehicle	50 mg/kg/d RASi x6 +1d	50 mg/kg/d RASi x6, +7d
**Liver**			
**(1)** Hepatocyte hypertrophy due to increased eosinophilic cytoplasm (lobular marginal)	0/4	0/4	4/4
**(2)** Decreased glycogen granules (lobular marginal)	0/4	4/4	4/4
**(3)** Clarification of the lobular structure, associated with the above changes	0/4	4/4	4/4
**Kidneys**			
**(4)** Basophilic changes in tubular epithelium	0/4	0/4	2/4
**(5)** Proximal tubular epithelial injury (nuclear condensation, tubular degeneration, tubular necrosis)	0/4	0/4	2/4
**Spleen**: no abnormality			

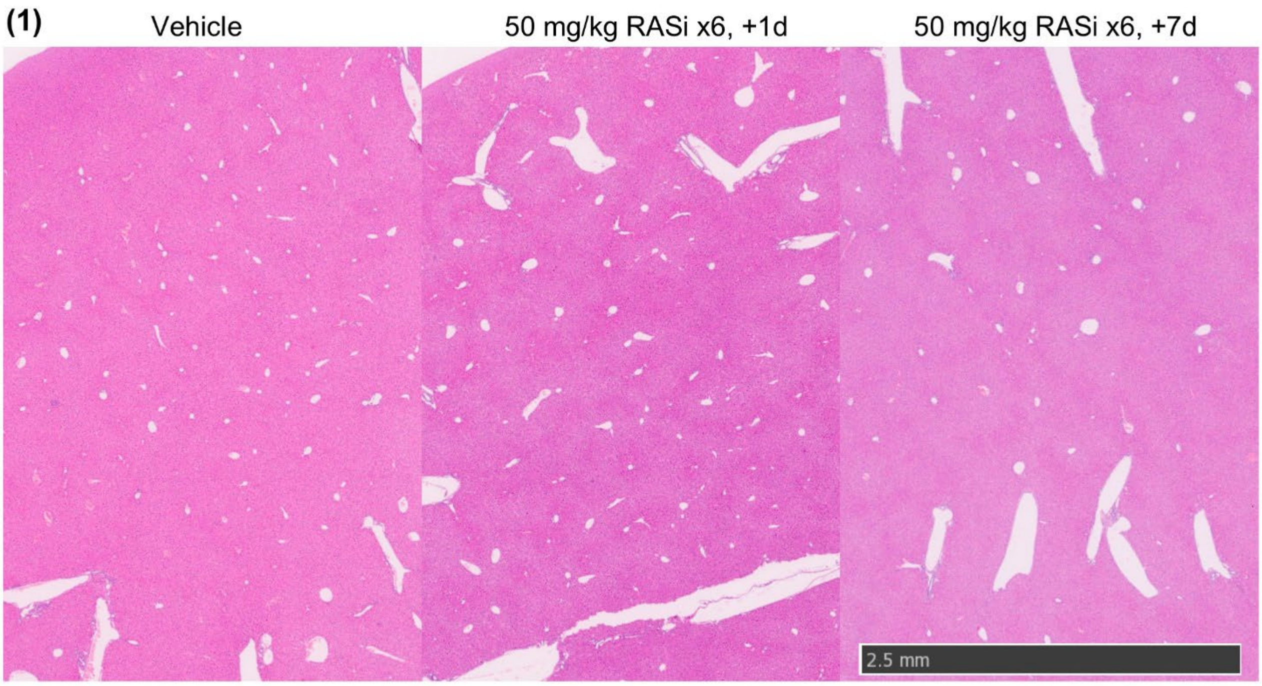

(2)

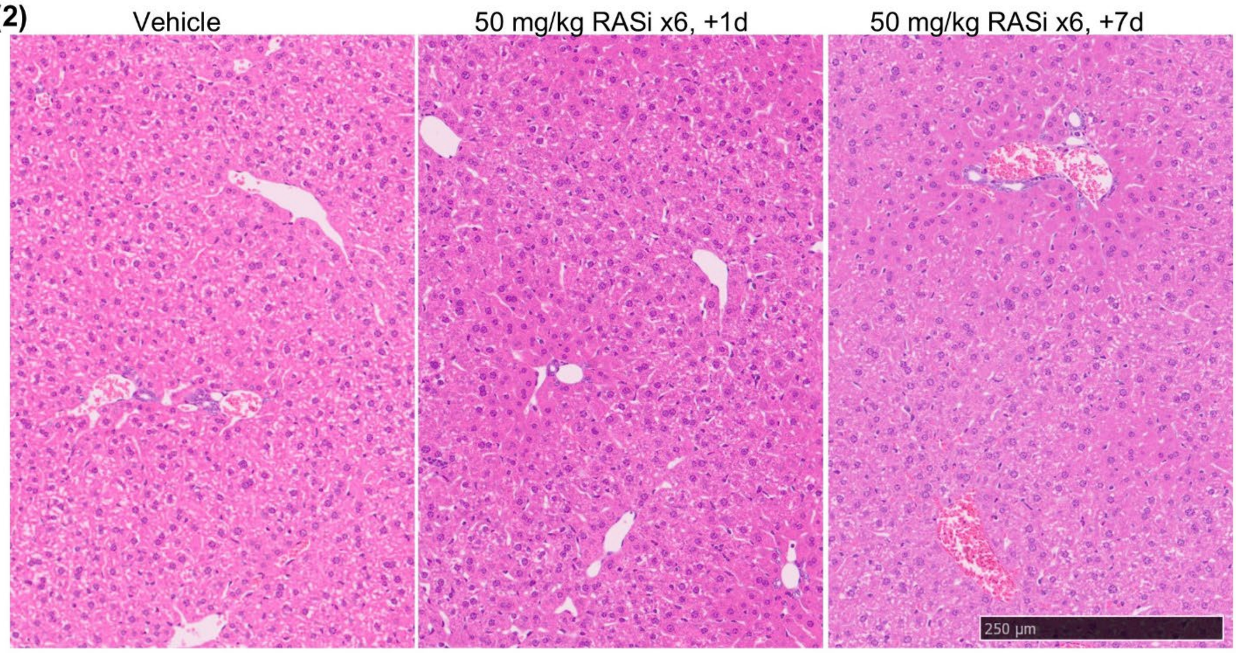
Vehicle
50 mg/kg RASi x6, +1d
50 mg/kg RASi x6, +7d
250 µm

(3)

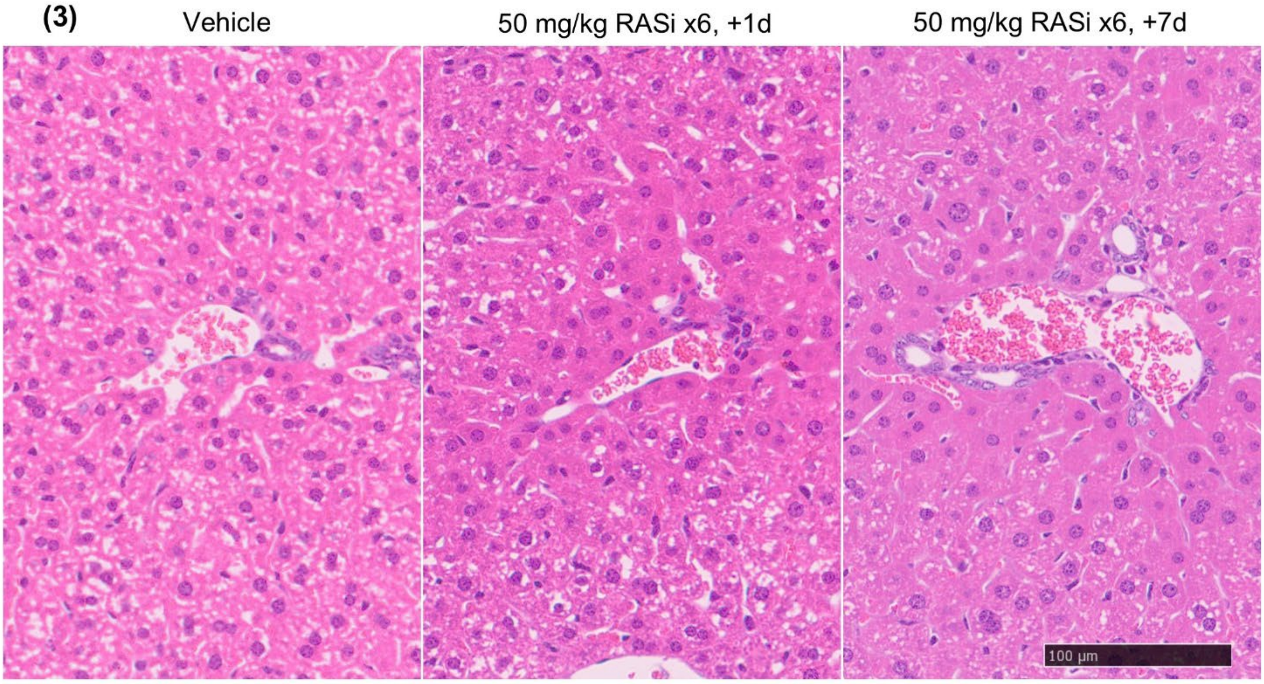
Vehicle
50 mg/kg RASi x6, +1d
50 mg/kg RASi x6, +7d
100 µm

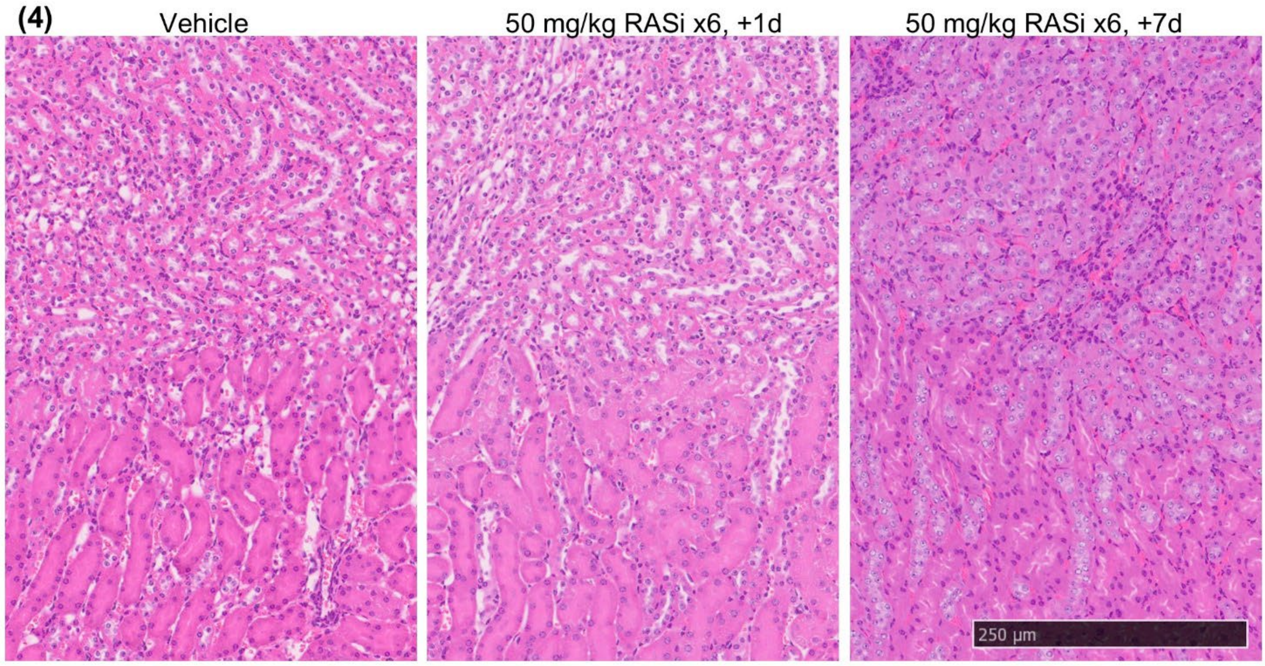

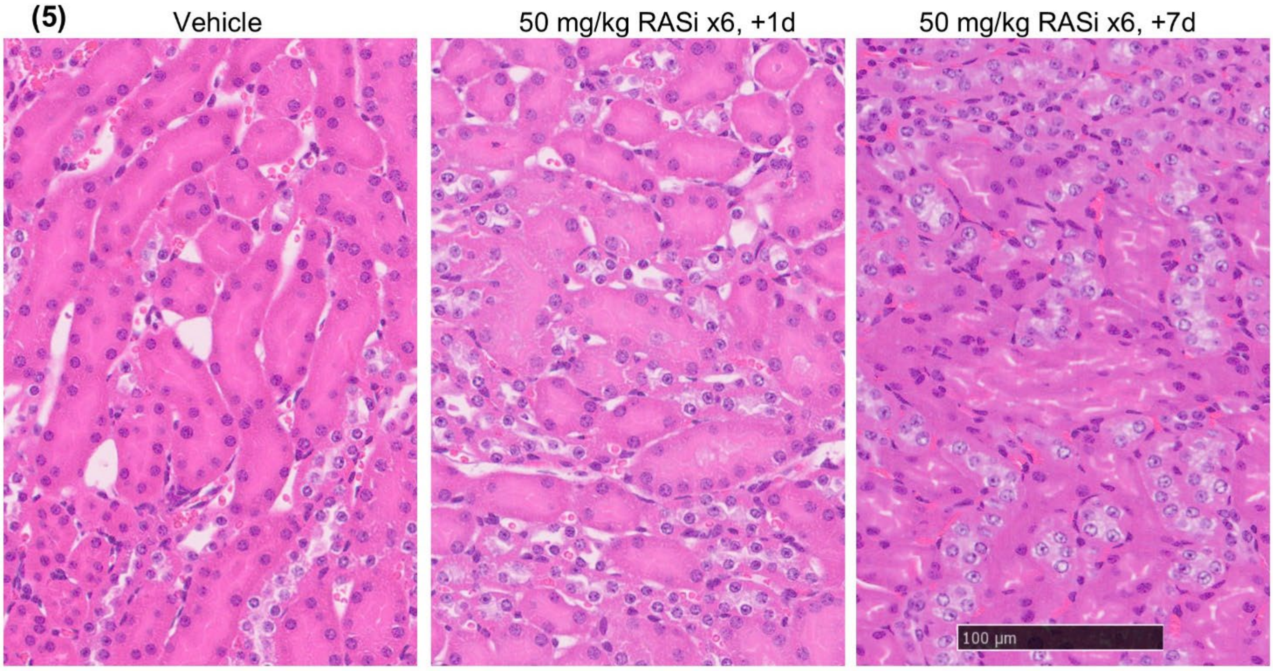

Figure S24. Histopathological assessment of liver, kidney, and spleen from non-tumor-bearing 8-week-old female C57BL/6 mice treated with six doses of 50 mg/kg/day RRSP-RBD-TAT and collected 1 or 7 days later. The summary table indicates the incidence of each histological finding (number of mice with the finding/4 mice examined). Representative H&E-stained sections are shown below.

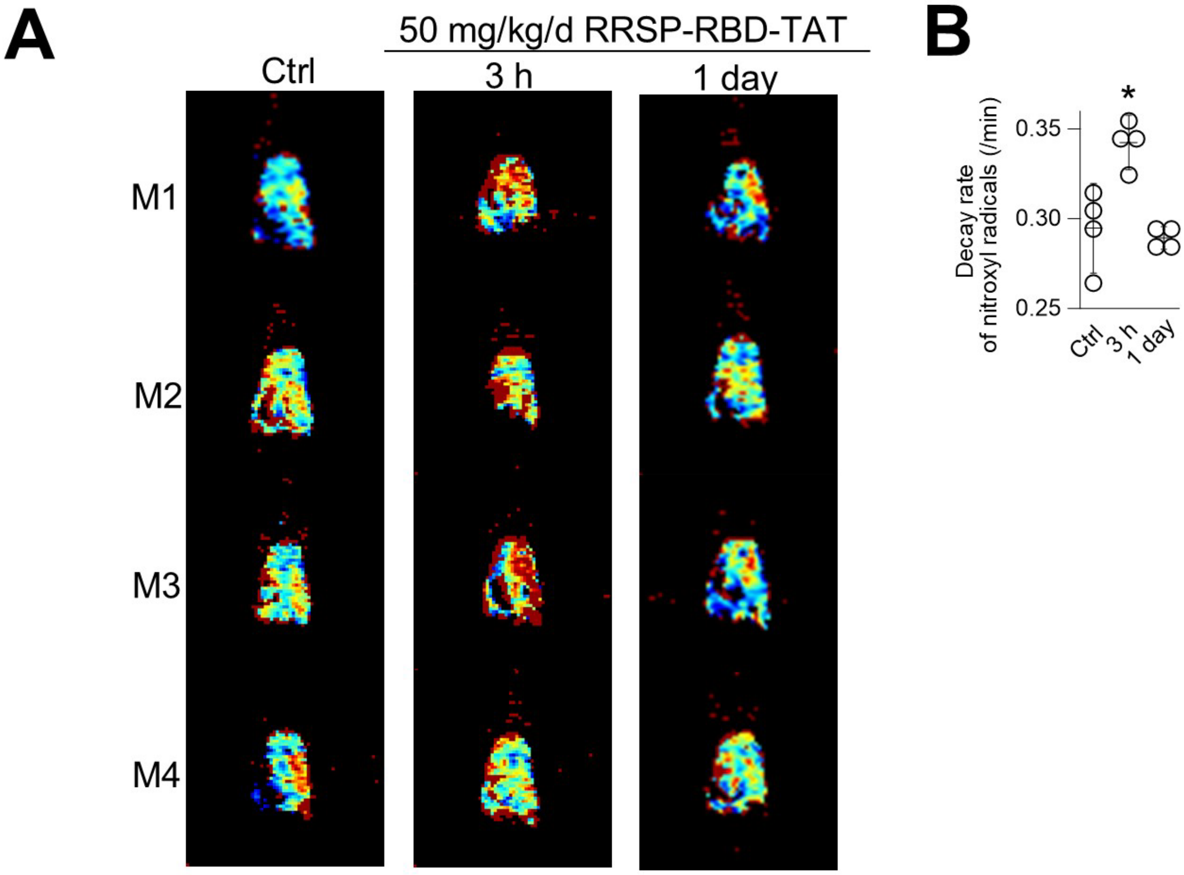

Figure S25. (A) Redox MRI images of liver region from the Colon-26 subcutaneous model (BALB/C) treated with 50 mg/kg RRSP-RBD-TAT. (B) Nitroxyl radical decay rates in the liver region. *P < 0.05, unpaired two-tailed t-test vs. control.

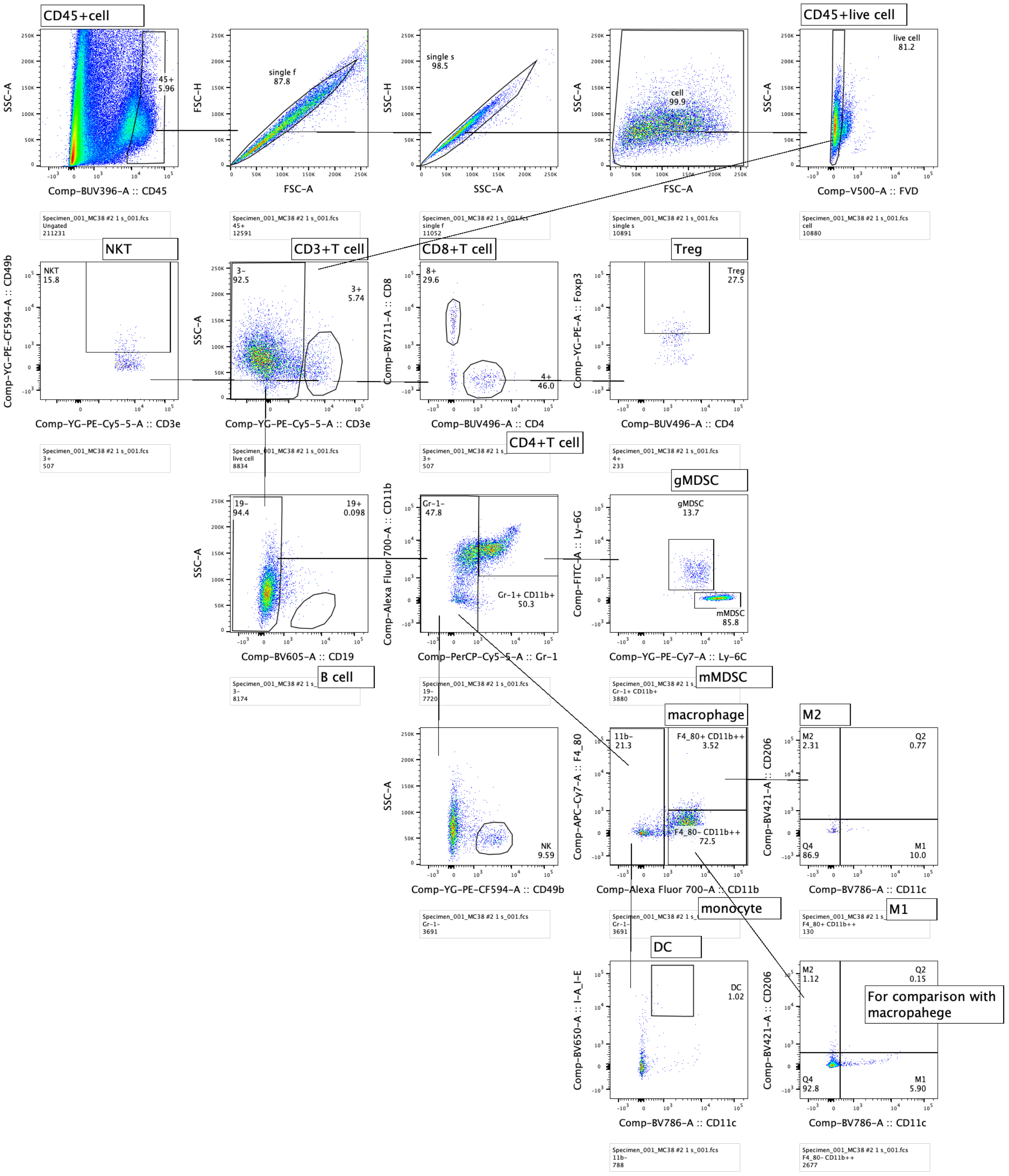

Figure S26. Flow-cytometry gating strategy for the lineage panel used for tumor-infiltrating lymphocyte analysis.

Sequential gating strategy used to identify tumor-infiltrating lymphocyte populations in dissociated tumor samples. After exclusion of debris, doublets, and dead cells, live leukocytes were gated and the indicated lineage populations were identified for quantification in Fig. 4D and Fig. S18.

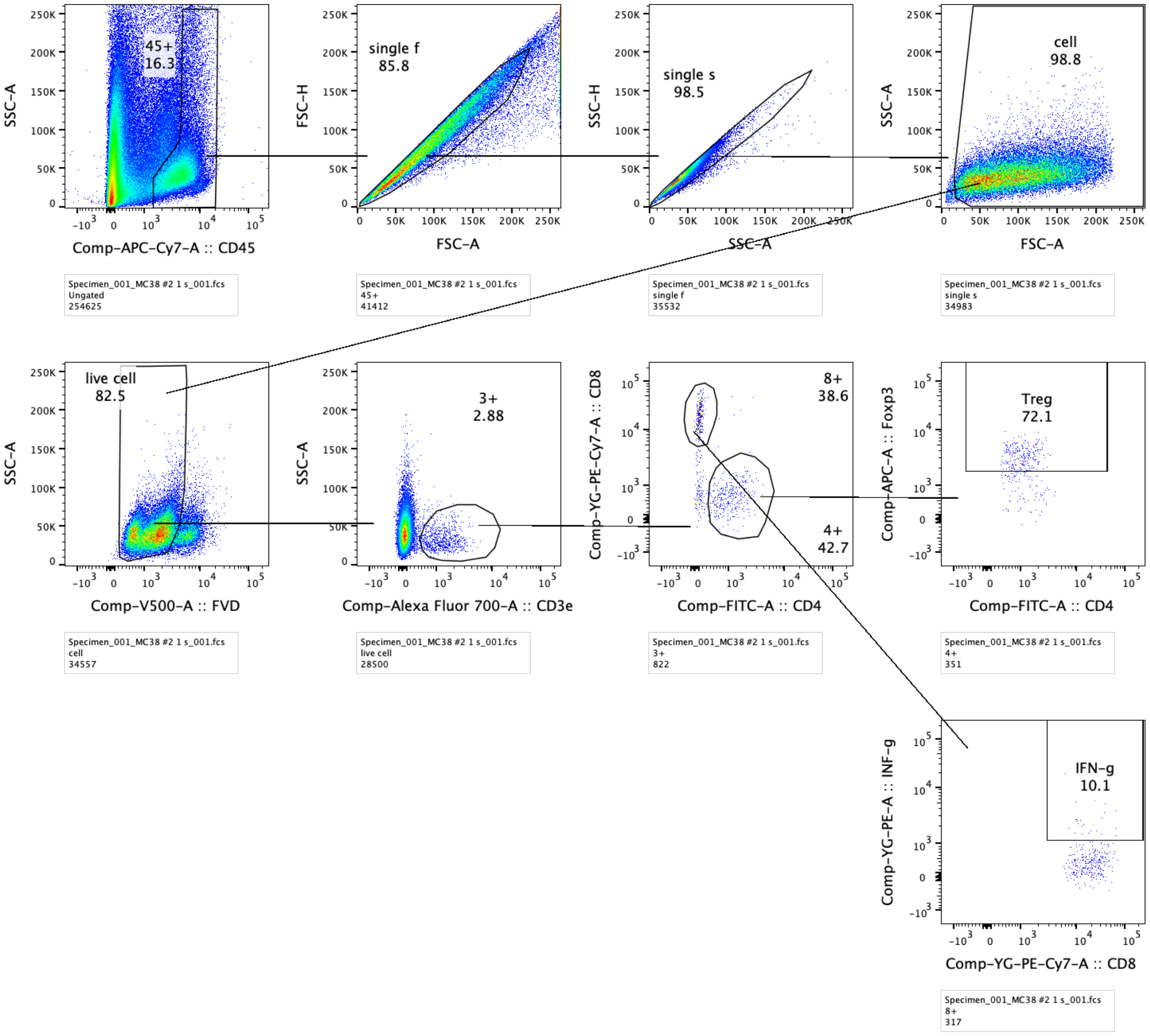

Figure S27. Flow-cytometry gating strategy for the T-cell IFNγ panel.

Sequential gating strategy used to quantify IFNγ-producing T-cell populations in dissociated tumor samples. After exclusion of debris, doublets, and dead cells, T cells were gated and IFNγ+ CD8+ T cells were identified by intracellular staining. This panel corresponds to the analysis shown in Fig. 4F.
