# Supplementary material for: Protein-based pan-RAS inhibitor induces tumor regression in female mice via IFNγ and CD8+ T cell-dependent tumor necrosis
Source: Nat Commun. 2026 May 16;17:6495. doi: 10.1038/s41467-026-73300-z (PMC13376784; doi:10.1038/s41467-026-73300-z)
Supplement: Supplementary file 2 — Description of Additional Supplementary Files [file 41467_2026_73300_MOESM2_ESM.pdf]

Description of Additional Supplementary Files

File Name: Supplementary Data 1

Description: Human and mouse cancer cell lines used in this study and their response parameters to RRSP-RBD-DTB and RRSP-RBD-TAT.

The table lists the tissue origin, species, RAS mutation status/genotype, and the corresponding EC50 and AUC values used in Figs. 2A, 3A, and 3D. This is a descriptive table; no inferential statistical comparisons are presented.

File Name: Supplementary Data 2

Description: RRSP-RBD-CPP constructs screened in this study and their in vitro activities in CT-26 cells.

The table lists the RBD/CPP combinations of the screened constructs together with the corresponding EC50 values measured in CT-26 cells. This is a descriptive screening table; no inferential statistical comparisons are presented.

File Name: Supplementary Data 3

Description: Serum biochemistry and hematology parameters in non-tumor-bearing female C57BL/6 mice treated with RRSP-RBD-TAT under the indicated dosing regimens.

Blood was collected 1 day or 1 week after the final dose following six daily doses of RRSP-RBD-TAT (25 or 50 mg/kg/day). Fold changes were calculated relative to the corresponding vehicle control group at the same time point. P values were calculated using two-sided unpaired t-tests versus the matched vehicle control group for each parameter, and exact P values are provided in the table (n = 4 mice per group).

File Name: Supplementary Data 4

Description: Exact P values for the statistical comparisons shown in Figs. 3c, 4e, and S20c.

For Fig. 3c, the table lists the exact P values for the comparison between vehicle- and RRSP-RBD-TAT-treated groups in each mouse model. For Fig. 4e and S20c, the table lists the exact P values for the indicated pairwise comparisons. P values were calculated using a two-sided unpaired t-test for Fig. 3c and one-way ANOVA followed by Tukey’s multiple-comparisons test for Fig. 4e and S20c.
