# Supplementary material for: Protein-based pan-RAS inhibitor induces tumor regression in female mice via IFNγ and CD8+ T cell-dependent tumor necrosis
Source: Nat Commun. 2026 May 16;17:6495. doi: 10.1038/s41467-026-73300-z (PMC13376784; doi:10.1038/s41467-026-73300-z)
Supplement: Supplementary file 7 — Reporting Summary [file 41467_2026_73300_MOESM7_ESM.pdf]

nature portfolio

Corresponding author(s): Ryo Honda

Last updated by author(s): Mar 29, 2026

Reporting Summary

Nature Portfolio wishes to improve the reproducibility of the work that we publish. This form provides structure for consistency and transparency in reporting. For further information on Nature Portfolio policies, see our Editorial Policies and the Editorial Policy Checklist.

Statistics

For all statistical analyses, confirm that the following items are present in the figure legend, table legend, main text, or Methods section.

n/a	Confirmed	
☐	☒	The exact sample size (*n*) for each experimental group/condition, given as a discrete number and unit of measurement
☐	☒	A statement on whether measurements were taken from distinct samples or whether the same sample was measured repeatedly
☐	☒	The statistical test(s) used AND whether they are one- or two-sided<br>*Only common tests should be described solely by name; describe more complex techniques in the Methods section.*
☒	☐	A description of all covariates tested
☐	☒	A description of any assumptions or corrections, such as tests of normality and adjustment for multiple comparisons
☐	☒	A full description of the statistical parameters including central tendency (e.g. means) or other basic estimates (e.g. regression coefficient) AND variation (e.g. standard deviation) or associated estimates of uncertainty (e.g. confidence intervals)
☐	☒	For null hypothesis testing, the test statistic (e.g. F, t, r) with confidence intervals, effect sizes, degrees of freedom and P value noted<br>*Give P values as exact values whenever suitable.*
☒	☐	For Bayesian analysis, information on the choice of priors and Markov chain Monte Carlo settings
☒	☐	For hierarchical and complex designs, identification of the appropriate level for tests and full reporting of outcomes
☒	☐	Estimates of effect sizes (e.g. Cohen's *d*, Pearson's *r*), indicating how they were calculated

Our web collection on statistics for biologists contains articles on many of the points above.

Software and code

Policy information about availability of computer code

Data collection	Data collection Confocal microscopy: images were acquired on a ZEISS LSM 910 in Airyscan 2 mode. Whole-slide imaging: tissue sections were scanned on an Olympus VS-120 virtual slide scanner. PET: emission data were collected on a Siemens Inveon PET/CT in 3-D list mode. In vivo/ ex vivo fluorescence: images were acquired on an IVIS Lumina S5 (Revvity). Flow cytometry: data were collected on a BD LSRFortessa flow cytometer. Luminex cytokine assays: measurements were obtained on a Luminex 200 system using the MILLIPLEX® panel. Hyperpolarized 13C MRS/proton MRI: hyperpolarization used a 6.7 T SpinAligner (Polarize), and proton MRI used a 1.5 T MR VioLVA small-animal system. In vivo DNP-MRI employed a low-field system (Keller; Japan Redox Ltd.). BRET/luminescence: NanoBRET signals were collected using a SpectraMax iD5 plate reader. Microarray expression profiling was performed on Agilent SurePrint G3 Human GE 8×60K v3.0 microarrays.
Data analysis	Western-blot densitometry and DNP-MRI image analysis: ImageJ (NIH) was used for band-intensity quantification and DNP-MRI image analysis. Flow cytometry: FlowJo v10.8.1 (BD/Tree Star) was used for gating and quantification. Gene set enrichment: GSEA v4.2.2 with MSigDB v7.5.1 was used for microarray-based enrichment analyses (default parameters; 1,000 permutations). PET reconstruction and TAC analysis: microPET Manager (Fourier rebinning + 2-D filtered back-projection) and ASIPro VM (Siemens) were used. IVIS quantification: Living Image software (Revvity) was used. Structural analysis: MOE (MOLSIS), Contact Analysis module, was used. No custom code was developed for this study.

For manuscripts utilizing custom algorithms or software that are central to the research but not yet described in published literature, software must be made available to editors and reviewers. We strongly encourage code deposition in a community repository (e.g. GitHub). See the Nature Portfolio guidelines for submitting code & software for further information.

Data

Policy information about availability of data

All manuscripts must include a data availability statement. This statement should provide the following information, where applicable:

- Accession codes, unique identifiers, or web links for publicly available datasets
- A description of any restrictions on data availability
- For clinical datasets or third party data, please ensure that the statement adheres to our policy

The microarray data generated in this study have been deposited in ArrayExpress under accession code E-MTAB-16847. Published structural data used in this study are available in the Protein Data Bank under accession code 6NTC. Source data underlying the main figures and relevant Supplementary Figures are provided as a Source Data file. All other supporting data are available within the Article, Supplementary Information, and Supplementary Tables.

Research involving human participants, their data, or biological material

Policy information about studies with human participants or human data. See also policy information about sex, gender (identity/presentation), and sexual orientation and race, ethnicity and racism.

Reporting on sex and gender	The study did not involve human participants or human data.
Reporting on race, ethnicity, or other socially relevant groupings	The study did not involve human participants or human data.
Population characteristics	No human participants were included.
Recruitment	No human participants were included.
Ethics oversight	No human participants, human data, or human biological materials were involved.

Note that full information on the approval of the study protocol must also be provided in the manuscript.

Field-specific reporting

Please select the one below that is the best fit for your research. If you are not sure, read the appropriate sections before making your selection.

- [x] Life sciences
- [ ] Behavioural & social sciences
- [ ] Ecological, evolutionary & environmental sciences

For a reference copy of the document with all sections, see nature.com/documents/nr-reporting-summary-flat.pdf

Life sciences study design

All studies must disclose on these points even when the disclosure is negative.

Sample size	In vivo efficacy cohorts generally used n = 5–6 mice per group; PD/PK/biodistribution cohorts used n = 2–5 mice per group; toxicology/histopathology used n = 4 mice per group. Cell-viability assays used n = 3 technical replicate wells unless otherwise noted. Sample sizes were chosen on the basis of prior experience with these models and the expected magnitude of treatment effects.
Data exclusions	No data were excluded a priori. Technical failures (for example, bacterial contamination or compromised tissue sections) were rerun when feasible.
Replication	Key findings were reproduced in independent experiments and across multiple models. RRSP-RBD-TAT efficacy was confirmed in several syngeneic tumors (CT-26, MC-38, and Colon-26), and pharmacodynamic target engagement was observed across models. The numbers of independent repeats for representative blots and micrographs are stated in the corresponding figure legends and Supplementary Information.
Randomization	Mice were randomized to treatment or vehicle when cohort mean tumor volume reached ~60 mm^3 (or ~120 mm^3 for delayed-start cohorts, where specified), typically 4–16 days post-inoculation. Randomization occurred at the animal level with groups balanced by baseline tumor volume.
Blinding	Investigators were not blinded to group allocation during dosing or caliper measurements due to operational constraints.

Reporting for specific materials, systems and methods

We require information from authors about some types of materials, experimental systems and methods used in many studies. Here, indicate whether each material, system or method listed is relevant to your study. If you are not sure if a list item applies to your research, read the appropriate section before selecting a response.

Materials & experimental systems

n/a	Involved in the study	
☐	☒	Antibodies
☐	☒	Eukaryotic cell lines
☒	☐	Palaeontology and archaeology
☐	☒	Animals and other organisms
☒	☐	Clinical data
☒	☐	Dual use research of concern
☒	☐	Plants

Methods

n/a	Involved in the study	
☒	☐	ChIP-seq
☒	☐	Flow cytometry
☒	☐	MRI-based neuroimaging

Antibodies

Antibodies used	All primary and secondary antibodies, including target, clone/host, vendor, catalog number, lot number where available, and working dilution, are listed in “Key resource (Antibodies).xlsx”.
Validation	Antibodies were selected on the basis of manufacturer validation and prior literature. Validation information for the primary antibodies is provided in “Key resource (Antibodies).xlsx”.

Eukaryotic cell lines

Policy information about cell lines and Sex and Gender in Research

Cell line source(s)	All human and mouse cell lines used in this study, together with their repository/supplier and catalog IDs, are listed in “Key resource (Cell lines).xlsx”.
Authentication	For cell lines obtained from certified repositories/suppliers, we relied on the provider’s authentication. For lines received from collaborators or whenever cross-contamination was suspected, we performed STR profiling by a commercial provider (Biologica Co., Ltd., Japan) prior to experimental use.
Mycoplasma contamination	All cell lines were routinely screened using the MycoAlert™ Mycoplasma Detection Kit (Lonza) and were negative at the time points relevant to the experiments.
Commonly misidentified lines (See ICLAC register)	Because HT-29 and WiDr have been reported as commonly misidentified/duplicate, we treated WiDr as an HT-29-related line. No other lines in our panel are listed by ICLAC as commonly misidentified.

Animals and other research organisms

Policy information about studies involving animals; ARRIVE guidelines recommended for reporting animal research, and Sex and Gender in Research

Laboratory animals	Female BALB/c, C57BL/6, and BALB/c-nu/nu mice (6–8 weeks old; Japan SLC) were used. Subcutaneous models were established by injecting 3–15 × 10^6 cells in 100 µL DPBS(-) into the right flank; an intramuscular CT-26 model was established by injection into the right thigh. Animals were randomized when cohort mean tumor volume reached ~60 mm^3 (~120 mm^3 for delayed-start cohorts where specified), typically 4–16 days post-inoculation. Exact n values for each experiment are given in the figure legends and source data. Mice were housed under SPF conditions (controlled temperature and humidity, 12-h light/dark cycle, food and water ad libitum).
Wild animals	The study did not involve wild animals.
Reporting on sex	All in vivo studies used female mice. Sex was not evaluated as an experimental variable; a single sex was used consistently across models to reduce inter-experimental variability.
Field-collected samples	No field-collected samples were used.
Ethics oversight	All animal procedures were approved by the Committees for Animal Research and Welfare of Gifu University and Tokushima University and conducted in accordance with institutional guidelines. Protocol numbers should be inserted in the final version where applicable.

Note that full information on the approval of the study protocol must also be provided in the manuscript.

Plants

Seed stocks	*Report on the source of all seed stocks or other plant material used. If applicable, state the seed stock centre and catalogue number. If plant specimens were collected from the field, describe the collection location, date and sampling procedures.*
Novel plant genotypes	*Describe the methods by which all novel plant genotypes were produced. This includes those generated by transgenic approaches, gene editing, chemical/radiation-based mutagenesis and hybridization. For transgenic lines, describe the transformation method, the number of independent lines analyzed and the generation upon which experiments were performed. For gene-edited lines, describe the editor used, the endogenous sequence targeted for editing, the targeting guide RNA sequence (if applicable) and how the editor was applied.*
Authentication	*Describe any authentication procedures for each seed stock used or novel genotype generated. Describe any experiments used to assess the effect of a mutation and, where applicable, how potential secondary effects (e.g. second site T-DNA insertions, mosiacism, off-target gene editing) were examined.*
