# Supplementary material for: Protein-based pan-RAS inhibitor induces tumor regression in female mice via IFNγ and CD8+ T cell-dependent tumor necrosis
Source: Nat Commun. 2026 May 16;17:6495. doi: 10.1038/s41467-026-73300-z (PMC13376784; doi:10.1038/s41467-026-73300-z)
Supplement: Supplementary file 8 — Transparent Peer Review file [file 41467_2026_73300_MOESM8_ESM.pdf]

nature portfolio

Peer Review File

A Protein-based Pan-RAS Inhibitor Induces Tumor Regression via IFNγ and CD8+ T Cell-Dependent Tumor Necrosis

Corresponding Author: Dr Ryo Honda

This file contains all reviewer reports in order by version, followed by all author rebuttals in order by version.

Version 0:

Reviewer comments:

Reviewer #1

(Remarks to the Author)
The study by Nomura et al describe the generation of a protein-based RAS inhibitors based on the fusion of RAS modifying enzymes to the Ras Binding Domain (RBD) that targets these enzymes to activated RAS. The authors utilize the Ras/Ral specific endopeptidase (RRSP) that specifically cleaves RAS between Y32-E33 to inactivate RAS. Fusion of the enzymatic portion of this protein to the RBD created a protein based inhibitor that blocks RAS on two levels: the RBD inhibits effector binding and the RRSP proteolytically cleaves RAS. They target this novel inhibitor to cells using different peptide sequences including a fragment of Diphtheria toxin which binds HBEGF as well as cell penetrating peptide such as TAT. Overall the data are robust and convincing in the demonstration of RAS inhibition, particularly with the RRSP-RBD-DTB protein. However, this inhibitor is limited in its use to human cells due to the inability of mouse cells to bind DTB due to a mutation in HBEGF. Thus the author turned to using a CPP to induce entry into mouse cells to better evaluate the pharmacology and toxicity in whole animals. The RRSP-RBD-TAT results in tumor inhibition but only in syngeneic models. Given the explosion in development of RAS pharmacologics, this paper is of great interest to the field to better understand how targeting RAS through different approaches can lead to effective tumor control or regression. However, the authors should provide additional data on the characterization of the RRSP-RBD-TAT protein. While there is significant in vivo data on demonstrating the antitumor activity in syngeneic models, it would be useful to provide additional data on the activity of this inhibitor in cells. Specifically,

The authors should examine the effects of RRSP-RBD-TAT on RAS cleavage, signaling pathway activation eg ERK and AKT, cell survival and anchorage independent growth in various human tumor cell lines in vitro. These data were provided for the RRSP-RBD-DTB but were conspicuously absent for the RRSP-RBD-TAT variant.

Also it would be useful if the authors could provide additional experimental insight as to why the RRSP-RBD-TAT protein only works in syngeneic models. Given that targeted RAS inhibition in RAS mutant tumor cell lines leads to inhibition of tumor formation, it is unclear why this reagent is not working in xenograft models. Perhaps use of more RAS-addicated lines such as PDAC lines would be more appropriate.

Overall however, I believe this to be an important study worth publication pending these modifications.

Reviewer #2

(Remarks to the Author)
The manuscript from the Honda group describes the application of a Ras/Rap1A-specific endopeptidase (RRSP) derived from bacteria that can cleave the RAS oncoprotein against tumor systems. This approach has been previously reported by more than one group but there are several novel aspects to the work described by the authors. Here the agent is enhanced by fusion to a RAS binding domain and further fused to a Cell penetrating peptide (CPP) to allow intracellular penetration. A Diphtheria toxin B subunit conjugate is also tested and appears much more effective, but as this does not penetrate mouse cells, the work focuses primarily on the CPP variant.

In vitro, the CPP agent was shown to be effective at degrading RAS at low nanomolar concentrations in a human cell line.

However, in vivo testing showed that a maximal dose of the agent had little or no effect on tumor growth when human tumor cell lines were injected into nu/nu mice.
In contrast, several murine cell lines showed what appear to be significant tumor growth inhibition (p values do not appear to be provided) with two of them, one mutant one wild type RAS, showing apparent curative events in over half the animals. Curiously, when the cell lines were tested in immune-deficient nu/nu mice, they were not sensitive to inhibition.

Examination of one cell line showed that when CD8+ T-cells and IFNg were depleted, the necrosis previously observed in the tumors was reduced. Moreover, examination of several other murine cell lines in the tumor model showed that levels of CD8+ T-cells and IFNg correlated with the degree of tumor inhibition.

The overall conclusion being that this modified RRSP approach has clinical potential and that the mechanism of action may differ somewhat from the new small molecule RAS inhibitors in or entering the clinic, as the immune response provoked is different and critical.

Previous attempts to use large bio-molecules such as this in a therapeutic setting have suffered from the induction of host immune responses that negate their long-term practical utility. In this case, only six infusions over two weeks were sufficient to induce multiple permanent regressions in the sensitive cells. In addition, the authors show no apparent serious toxicity despite wide distribution of the agent in vivo.

Whereas the report has many interesting features, there are some serious concerns, with interpretation and presentation of the data.

1. Although the RPPS was described as “pan-RAS”, its actual activity in its conjugated form is not shown. Exactly which RAS proteins does it cleave? Does it also act on M and R RAS members, Rit or Rheb? The antibody used to assay RAS is not fully described.
2. These proteases usually also cleave Rap. This can lead to toxicity due to disruption of endothelial cell function. Rap can also act as an important modulator of T-cell responses. What is the Rap status of the cells after treatment and what is the role of Rap inactivation in the immune effects observed?
3. Was RAS cleavage and P.ERK inhibition achieved in the human tumors that were not inhibited?
4. What is the status of RAS and P.ERK in the murine cell line tumors that were not inhibited?
5. Why does the cleavage of RAS in the wild type cell lines appear to have no effect on P.ERK. It would be reasonable to assume at least some signaling was coming through the wild type RAS?
6. What does a pan-RAS inhibitor such as RMC-7977 do to CT-26 and MC-38 cells in contrast to the RRSP?
7. Why does P.ERK increase in vivo in the treated nu/nu Colon-26 tumors but not in the BALBC tumors? Figure 4C. The RAS pathway is complex with many feedback loops – is this activation pertinent to the inhibitory effects? Variations between the two samples make the MC-38 data here impossible to interpret.
8. I worry that most of the in vivo data may be flawed because of the way in which the experiments were performed. Typically, in this type of study, the tumors are allowed to establish and reach 100-200 mm3 before entering into a drug study. It is fairly well demonstrated that the smaller the tumor is when the experiment starts the easier it is to inhibit. Here the first treatment was typically started between 4-16 days after cell injection, when the average tumor size was only 60mm3. It is quite possible that differences in tumor size when entering the study could have impacted the results, making the observation “prevention” of tumor development rather than regression.
9. The “fold” variations described in the text for figure 4D do not appear to correlate with the numbers in the Figure. Is this really log10 ?
10. Figure 4F x-axis may have been mislabeled ?
11. The T-cell depletion assays were only performed in one cell line.

Version 1:

Reviewer comments:

Reviewer #1

(Remarks to the Author)
This is a resubmission of a study examining the protein-based RAS inhibitor consisting of the RRSP fused to the RBD and then attached to various cell penetrating sequence. While the authors demonstrate that this reagent induces RAS cleavage in model systems and in some cased tumors, there remain questions regarding the interpretation of results using the RRSP-RBD-TAT reagent. The authors state that this agent enters cells and promotes RAS cleavage yet is unable to induce tumor inhibition in athymic mouse systems. This result is perplexing given that many human RAS mutant cell lines have been shown to be dependent on RAS for their tumorigenic activity in athymic nude mice. For example, MiaPaCa-2 cells are inhibited in their tumorigenic capacity using AMG510 (see Canon et al Nature 2019). Also, it is difficult to interpret Fig S17C as the RAS bands in MiaPaCa2 samples are quite faint and it is difficult to discern whether there was significant RAS cleavage.
SW480 cells are inhibited in tumor formation by RRSP-RBD-DTB but not with RRSP-RBD-TAT. Authors should

demonstrate engagement of RAS in both tumor models. It appears that the DTP version does indeed cleave RAS in vitro in SW480 but no data shown for tumor even though there is significant tumor inhibition. In addition, the TAT version does not apparently result in KRAS cleavage in SW480 in vitro as shown in Fig S8 but does apparently cleave RAS in nu/nu tumors as shown in Fig. S17C. These results are inconsistent. If Fig. 17C is correct, then why is there not tumor suppression? This discrepancy begs the question of whether their RRSP-RBD-TAT reagent is working as they propose.
The authors have not convincingly shown the specificity for just H, K, and NRAS as raised in the prior review. The RBD targets other RAS proteins including RAP and RRAS proteins suggesting that the effects both in tumor cells and the TME including immune cells could be due in part to effects outside of the canonical H, K, and NRAS.

Minor point
Fig 3. I and J legend does not explain what the two different lanes in each sample represent. If from two tumors (assume >5 were analyzed), how many total were available and why are the others not included?
Fig. 5C. Please indicate the meaning of arrow in legend.

Reviewer #2

(Remarks to the Author)
I would like to congratulate the authors on their comprehensive experimental response to my concerns.

They may wish to consider mentioning the results obtained on Rap1a as "little to no obvious effect" and including the figure in the supplementary data.

Version 2:

Reviewer comments:

Reviewer #1

(Remarks to the Author)
The authors responses and changes have addressed my concerns.

Open Access This Peer Review File is licensed under a Creative Commons Attribution 4.0 International License, which permits use, sharing, adaptation, distribution and reproduction in any medium or format, as long as you give appropriate credit to the original author(s) and the source, provide a link to the Creative Commons license, and indicate if changes were made.
In cases where reviewers are anonymous, credit should be given to 'Anonymous Referee' and the source.
The images or other third party material in this Peer Review File are included in the article’s Creative Commons license, unless indicated otherwise in a credit line to the material. If material is not included in the article’s Creative Commons license and your intended use is not permitted by statutory regulation or exceeds the permitted use, you will need to obtain permission directly from the copyright holder.
To view a copy of this license, visit https://creativecommons.org/licenses/by/4.0/

Reviewer #1 (Remarks to the Author):

The study by Nomura et al describe the generation of a protein-based RAS inhibitors based on the fusion of RAS modifying enzymes to the Ras Binding Domain (RBD) that targets these enzymes to activated RAS. The authors utilize the Ras/Ral specific endopeptidase (RRSP) that specifically cleaves RAS between Y32-E33 to inactivate RAS. Fusion of the enzymatic portion of this protein to the RBD created a protein based inhibitor that blocks RAS on two levels: the RBD inhibits effector binding and the RRSP proteolytically cleaves RAS. They target this novel inhibitor to cells using different peptide sequences including a fragment of Diphtheria toxin which binds HBEGF as well as cell penetrating peptide such as TAT. Overall the data are robust and convincing in the demonstration of RAS inhibition, particularly with the RRSP-RBD-DTB protein. However, this inhibitor is limited in its use to human cells due to the inability of mouse cells to bind DTB due to a mutation in HBEGF. Thus the author turned to using a CPP to induce entry into mouse cells to better evaluate the pharmacology and toxicity in whole animals. The RRSP-RBD-TAT results in tumor inhibition but only in syngeneic models. Given the explosion in development of RAS pharmacologics, this paper is of great interest to the field to better understand how targeting RAS through different approaches can lead to effective tumor control or regression. However, the authors should provide additional data on the characterization of the RRSP-RBD-TAT protein. While there is significant in vivo data on demonstrating the antitumor activity in syngeneic models, it would be useful to provide additional data on the activity of this inhibitor in cells. Specifically,

The authors should examine the effects of RRSP-RBD-TAT on RAS cleavage, signaling pathway activation eg ERK and AKT, cell survival and anchorage independent growth in various human tumor cell lines in vitro. These data were provided for the RRSP-RBD-DTB but were conspicuously absent for the RRSP-RBD-TAT variant.

We thank the reviewer for this constructive suggestion. We agree that demonstrating on-target activity of the RRSP-RBD-TAT variant in human tumor cells strengthens the translational relevance of our study. While our cell-viability assays already established activity in human cell lines (Fig. 3A), as the reviewer notes, it is important to demonstrate RAS inhibition by immunoblot.

Accordingly, in the revised manuscript we performed new immunoblot analyses in three human cancer cell lines (SW480, 143B, and HCT116) (new Fig. S8). RRSP-RBD-TAT induced RAS cleavage and suppressed p-ERK in the nanomolar range; notably, in the most sensitive line (143B), robust RAS cleavage and ERK inhibition were evident at 31 nM. These biochemical effects occur at concentrations concordant with the cell-viability EC_{50} profiles reported in our original submission (Fig. 3A), supporting that RRSP-RBD-TAT functions as a RAS-cleaving/-inhibiting modality in human tumor cells as well.

We have added these data as Fig. S8 and revised the manuscript accordingly (lines 184–186).

Also it would be useful if the authors could provide additional experimental insight as to why the RRSP-RBD-TAT protein only works in syngeneic models. Given that targeted RAS inhibition in RAS mutant tumor cell lines leads to inhibition of tumor formation, it is unclear why this reagent is not working in xenograft models. Perhaps use of more RAS-addicated lines such as PDAC lines would be more appropriate.

We appreciate this important question. The differential activity of RRSP-RBD-TAT between syngeneic and xenograft settings is driven by host immune competence—specifically, the presence of IFNγ-producing $CD8^+$ T cells, as demonstrated by three independent lines of evidence. First, in a side-by-side comparison using the same mouse cancer cells (Colon-26 and MC-38), RRSP-RBD-TAT produced tumor necrosis and growth inhibition in immunocompetent BALB/c mice but had no effect in athymic nude mice (Fig. 4A and S16), despite robust intratumoral target engagement—RAS cleavage—in both contexts (Fig. 4B–C). Second, antibody-mediated depletion established necessity: depleting $CD8^+$ T cells or neutralizing IFNγ in the syngeneic Colon-26 and MC-38 models completely abrogated RRSP-RBD-TAT–induced tumor necrosis (Fig. 4E and S20). Third, baseline immune profiling showed that immunodeficient models harbor markedly fewer intratumoral T cells and lower IFNγ than immunocompetent counterparts (Fig. 4D), and across six models the proportion of $IFN\gamma^+$ $CD8^+$ T cells strongly correlated with drug efficacy (Fig. 4F). Collectively, these data support the conclusion that an $IFN\gamma^+$ $CD8^+$ T cell–competent microenvironment is required to translate on-target RAS cleavage into tumor necrosis/regression, explaining the lack of efficacy in immunodeficient xenografts.

Regarding the suggestion to test highly RAS-addicted PDAC lines: while we cannot exclude that certain PDAC models might retain activity, our current evidence argues that tumor-intrinsic RAS engagement alone is insufficient for regression in the absence of an intact adaptive immune response. For example, 143B cells are among the most sensitive to RRSP-RBD-TAT in vitro at tens-of-nanomolar concentrations (Fig. S8), yet show no response as xenografts in nude mice (Fig. 3C).

Overall however, I believe this to be an important study worth publication pending these modifications.

Reviewer #2 (Remarks to the Author):

The manuscript from the Honda group describes the application of a Ras/Rap1A-specific endopeptidase (RRSP) derived from bacteria that can cleave the RAS oncoprotein against tumor systems. This approach has been previously reported by more than one group but there are several novel aspects to the work described by the authors. Here the agent is enhanced by fusion to a RAS binding domain and further fused to a Cell penetrating peptide (CPP) to allow intracellular penetration. A Diphtheria toxin B subunit conjugate is also tested and appears much more effective, but as this does not penetrate mouse cells, the work focuses primarily on the CPP variant.

In vitro, the CPP agent was shown to be effective at degrading RAS at low nanomolar concentrations in a human cell line. However, in vivo testing showed that a maximal dose of the agent had little or no effect on tumor growth when human tumor cell lines were injected into nu/nu mice.

In contrast, several murine cell lines showed what appear to be significant tumor growth inhibition (p values do not appear to be provided) with two of them, one mutant one wild type RAS, showing apparent curative events in over half the animals. Curiously, when the cell lines were tested in immune-deficient nu/nu mice, they were not sensitive to inhibition.

Examination of one cell line showed that when CD8+ T-cells and IFNg were depleted, the necrosis previously observed in the tumors was reduced. Moreover, examination of several other murine cell lines in the tumor model showed that levels of CD8+ T-cells and IFNg correlated with the degree of tumor inhibition.

The overall conclusion being that this modified RRSP approach has clinical potential and that the mechanism of action may differ somewhat from the new small molecule RAS inhibitors in or entering the clinic, as the immune response provoked is different and critical.

Previous attempts to use large bio-molecules such as this in a therapeutic setting have suffered from the induction of host immune responses that negate their long-term practical utility. In this case, only six infusions over two weeks were sufficient to induce multiple permanent regressions in the sensitive cells. In addition, the authors show no apparent serious toxicity despite wide distribution of the agent in vivo.

Thank you for the thoughtful review. Guided by your comments, we added new in vitro and in vivo experiments to strengthen the central claims and integrated these data throughout the revision; details are provided in the point-by-point responses.

We also now report statistical significance by adding p-values to the syngeneic tumor-growth-inhibition figure (Fig. 3C).

Whereas the report has many interesting features, there are some serious concerns, with interpretation and presentation of the data.

1. Although the RPPS was described as “pan-RAS”, its actual activity in its conjugated form is not shown. Exactly which RAS proteins does it cleave? Does it also act on M and R RAS members, Rit or Rheb? The antibody used to assay RAS is not fully described.

We thank the reviewer for raising this point. In this manuscript, we use "pan-RAS" to denote activity against the three canonical RAS isoforms—KRAS, HRAS, and NRAS—and, in this sense, RRSP-RBD functions as a pan-RAS inhibitor. Three lines of evidence support this designation. First, the targeting module (cRaf-v1 RBD) binds all three isoforms and multiple mutants as reported previously (Cell Chem Biol. 2021; PMID 33964212), and our NanoBRET assays confirm engagement of KRAS-WT, KRAS-G12V, and KRAS-Q61R by the RRSP-RBD fusion (Fig. 1F). Second, prior work shows that RRSP cleaves RAS within Switch I (between Tyr32 and Glu33) based on sequence features conserved across K/H/N-RAS (and Rap1A) (Nat Commun. 2015; PMID 26051945), indicating that once recruited by the RBD, the enzyme retains access to the canonical cleavage site (Fig. S6 provides the amino-acid sequences of K/H/N-RAS, highlighting the RRSP cleavage site and the RBD-binding region). Third, in cell-viability assays (Figs. 2A, 3A and Table S1), both RRSP-RBD-DTB and RRSP-RBD-TAT showed activity across diverse K/H/N-RAS-mutant human cell lines (e.g., T24, RD), consistent with pan-isoform targeting.

Furthermore, to provide direct isoform-level evidence in the conjugated context, we performed new co-expression experiments in HEK293T cells: RRSP-RBD-2×HA was co-expressed with HRAS-WT, HRAS-G12V, KRAS-G12V, or NRAS-G12V. In all four settings, the fusion induced RAS cleavage and p-ERK suppression (new Fig. S3). Together, these data and the mechanistic rationale support RRSP-RBD as a pan-RAS (K/H/N) inhibitor.

We added the mechanistic rationale for RRSP-RBD as a pan-RAS inhibitor to the Discussion (lines 297–300), incorporated the co-expression results into Figure S3 (lines 123–127), and updated the Methods accordingly.

Regarding other RAS-family GTPases (MRAS, RRAS, RIT1/Rit, RHEB), these are not part of the canonical RAS isoforms and were not evaluated experimentally here. While susceptibility could, in principle, be inferred from RBD-binding profiles and Switch I conservation, a definitive assessment is beyond the scope of this work and would require dedicated assays. Finally, antibody details are specified in the Key Resources Table: the RAS antibody used for immunoblotting (CST Cat. No. 3965) recognizes K-, H-, and N-RAS.

2. These proteases usually also cleave Rap. This can lead to toxicity due to disruption of endothelial cell function. Rap can also act as an important modulator of T-cell responses. What is the Rap status of the cells after treatment and what is the role of Rap inactivation in the immune effects observed?

We appreciate this thoughtful point. To directly assess Rap status in immune cells, we purified $CD3^+$ T cells from mouse spleen, treated them in vitro with RRSP-RBD-TAT (0–2000 nM, 24 h), and probed Rap1A cleavage by immunoblot using two antibodies. In these exploratory assays, endogenous Rap1A signals were not reproducibly detectable with either reagent, precluding a definitive conclusion about Rap cleavage under our conditions. We infer that more sensitive detection approaches—for example, antibodies validated for endogenous murine Rap1A in primary T cells or targeted proteomics—will be required to quantify Rap processing rigorously. As the present study focuses on engineering a protein-based pan-RAS inhibitor and establishing its $IFN\gamma^+$ $CD8^+$ T-cell–dependent mechanism of tumor control, delineating the contribution of potential Rap inactivation in immune effects is outside our current scope; we flag this as an important direction for future work.

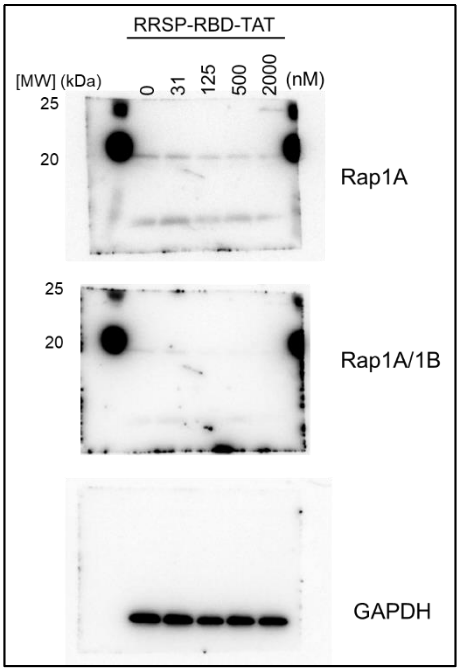

Figure 1 (numbering for point-by-point response). **Immunoblot analysis of primary mouse CD3⁺ T cells treated with RRSP-RBD-TAT for 24 h.**

Method. Spleens were harvested from untreated BALB/c mice, dissociated to single-cell suspensions, and cryopreserved in CELLBANKER® 1, following the workflow described in the Methods section ("Measurement of Tumor-Infiltrating Lymphocytes"). After thawing, $CD3^+$ T cells were isolated using MojoSort™ Mouse CD3 T Cell Isolation Kit (BioLegend, #480023) per the manufacturer's protocol. Cells were seeded at 5×10^5 viable cells/well in 24-well plates (RPMI-1640 + 10% FBS) and incubated at 37 °C, 5% CO_2 for 24 h. RRSP-RBD-TAT (0–2000 nM) was then added for an additional 24 h. Viable cell counts at harvest were ~$2–5 \times 10^5$/well with no consistent differences between vehicle and treated groups. Cells were pelleted, washed once with DPBS, and lysed in SDS sample buffer (2% SDS, 62.5 mM Tris-HCl [pH 6.8], 10% glycerol). 3.7 µg total protein per lane were resolved by SDS-PAGE and immunoblotted as described in the Methods. Primary antibodies were anti-Rap1A (Proteintech #16336-1-AP) and anti-Rap1A/1B (CST #2399); secondary antibodies are listed in the Key Resources. Predicted band size of Rap1A is 20 kDa.

3. Was RAS cleavage and P.ERK inhibition achieved in the human tumors that were not inhibited?

Thank you for this important question. We conducted pharmacodynamic analyses in three non-responding human xenograft models (HCT116, SW480, and MiaPaCa-2) to assess intratumoral target engagement by RRSP-RBD-TAT (new Fig. S17C). In all three models, immunoblotting of tumor lysates confirmed robust RAS cleavage, indicating effective target engagement in vivo; however, no consistent suppression of p-ERK was observed.

These data suggest that while target engagement (RAS cleavage) is model-independent, progression to sustained ERK suppression and tumor necrosis is model-dependent, likely influenced by tumor-intrinsic RAS dependence and the immune microenvironment—particularly $IFN\gamma^+$ $CD8^+$ T cells. This pattern is consistent with other comparative data (see our response to comments 4 and 7).

We have added the HCT116, SW480, and MiaPaCa-2 immunoblots to Fig S17C (lines 227-234).

4. What is the status of RAS and P.ERK in the murine cell line tumors that were not inhibited?

We appreciate this question. In two insensitive murine models (RENCA and LL/2), pharmacodynamic analysis of tumor lysates following RRSP-RBD-TAT administration showed clear RAS cleavage, indicating intratumoral target engagement (new Figure S17B). By contrast, p-ERK levels could not be reliably interpreted because basal p-ERK signals were low/variable in these tumors, precluding a reproducible decrement relative to vehicle.

These data suggest that while target engagement (RAS cleavage) is model-independent, progression to sustained ERK suppression and tumor necrosis is model-dependent, likely

influenced by tumor-intrinsic RAS dependence and the immune microenvironment—particularly IFNγ⁺ CD8⁺ T cells. This pattern is consistent with other comparative data (see our response to comments 3 and 7).

We have added the RENCA and LL/2 immunoblots to Figure S17B (lines 227-234).

5. Why does the cleavage of RAS in the wild type cell lines appear to have no effect on P.ERK. It would be reasonable to assume at least some signaling was coming through the wild type RAS?

We agree. Because ERK integrates diverse inputs upstream of RAF/MEK (RTKs/GPCRs, PKC/Src, RAC1/PAK) and is buffered by feedback, on-target RAS cleavage alone may be insufficient to lower p-ERK in RAS-wild-type settings where alternative drivers predominate.

6. What does a pan-RAS inhibitor such as RMC-7977 do to CT-26 and MC-38 cells in contrast to the RRSP?

Thank you for this constructive question. We profiled RMC-6236 (a close congener of RMC-7977) in vitro by WST-8 assays. The activity pattern paralleled that of RRSP-RBD-TAT: we observed robust growth inhibition in CT-26 (KRAS-G12D), whereas MC-38 and RENCA (both RAS-WT) showed little to no inhibition over the tested range. These preliminary data suggest that a pan-RAS small molecule and our protein-based modality may share a similar susceptibility ranking across these murine models. That said, a firm conclusion will require a broader cell-line panel.

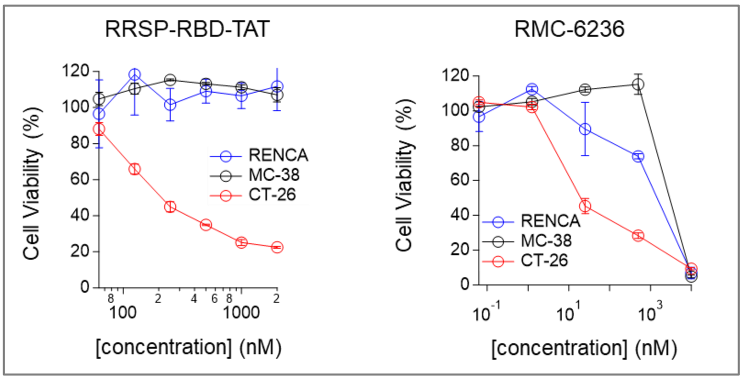

Figure 2 (numbering for point-by-point response)**. Head-to-head comparison of RMC-6236 and RRSP-RBD-TAT on murine cancer cell viability.**

Methods. RENCA, MC-38, and CT-26 cells were suspended in RPMI-1640 + 10% FBS and seeded into 96-well plates at a density expected to reach near-confluence in ~3 days. On the following day, inhibitors were added and cells were incubated for 48 h. Viability was measured using a WST-8 assay per the manufacturer's instructions. RMC-6236 was purchased from Cayman Chemical (#41982) and used from a 6.15 mM DMSO stock.

7. Why does P.ERK increase in vivo in the treated nu/nu Colon-26 tumors but not in the BALBC tumors? Figure 4C. The RAS pathway is complex with many feedback loops – is this activation pertinent to the inhibitory effects? Variations between the two samples make the MC-38 data here impossible to interpret.

We appreciate this helpful observation. To improve interpretability of the pharmacodynamic readouts, we added three biological replicates per group to our original two (new Fig. S17A). Consistent with our initial data, in the CT-26 (KRAS-G12D) nu/nu model, p-ERK showed a tendency to increase after RRSP-RBD-TAT (80%, 4/5 mice). By contrast, in the MC-38 (KRAS-WT) nu/nu model, p-ERK rebound was not observed; however, interpretation is limited because basal p-ERK signals were low and variable in MC-38 tumors.

These data indicate that, in an immunodeficient setting, RAS inhibition by RRSP-RBD-TAT in KRAS-G12D tumors can induce p-ERK rebound. In the presence of host immunity—especially IFNγ$^+$ CD8$^+$ T cells—RRSP-RBD-TAT and the immune microenvironment synergize to drive p-ERK inhibition and tumor necrosis/regression. That said, the observed rebound was modest and warrants confirmation in additional RAS-mutant syngeneic models. Thus, firm conclusions (and the precise feedback nodes that drive ERK rebound) are beyond the scope of this study.

We have added the corresponding immunoblots to Figure S17A (lines 227–234).

8. I worry that most of the in vivo data may be flawed because of the way in which the experiments were performed. Typically, in this type of study, the tumors are allowed to establish and reach 100-200 mm3 before entering into a drug study. It is fairly well demonstrated that the smaller the tumor is when the experiment starts the easier it is to inhibit. Here the first treatment was typically started between 4-16 days after cell injection, when the average tumor size was only 60mm3. It is quite possible that differences in tumor size when entering the study could have impacted the results, making the observation "prevention" of tumor development rather than regression.

We appreciate this methodological concern and agree that starting tumor size can influence the apparent effect size. However, our pan-RAS inhibitor can be used as a therapeutic that induces tumor regression rather than a preventive agent, for two reasons. First, multiple studies initiate treatment at ~60 mm^3 or 50–100 mm^3 (e.g., Communications Biology 2024, PMID 38755254; Cancer Immunology Research 2017, PMID 29097420), underscoring that our design is within field standards. Second, to address the possibility that our findings reflect prevention rather than regression, we performed delayed-start experiments in the CT-26 syngeneic model, initiating therapy at ~120 mm^3 (new Figure S12B). RRSP-RBD-TAT produced responses comparable to those seen with ~60 mm^3 starts—including 4/6 complete regressions—indicating bona fide therapeutic activity rather than merely delaying the outgrowth of small tumors.

We have added these delayed-start data to Figure S12B (lines 203–205). and updated the Methods accordingly.

9. The "fold" variations described in the text for figure 4D do not appear to correlate with the numbers in the Figure. Is this really log10 ?

Figure 4D reports absolute counts of tumor-infiltrating cells normalized per gram of tumor (cells/g) on a log10 scale; it is not a fold change.

10. Figure 4F x-axis may have been mislabeled ?

Thank you for catching this. The x-axis label in Figure 4F was indeed incorrect; it now reads "% tumor growth"

11. The T-cell depletion assays were only performed in one cell line.

We agree that replication across models is important, given our focus on the IFNγ$^+$ CD8$^+$ T-cell dependence of RRSP-RBD-TAT. We therefore performed additional depletion experiments in the MC-38 syngeneic model (which exhibits high baseline CD8$^+$/IFNγ levels; Fig. 4F). As in Colon-26, CD8$^+$ T-cell depletion or IFNγ neutralization markedly reduced RRSP-RBD-TAT–induced tumor necrosis (new Fig. S20). Furthermore, in a previous dataset we established MC-38 nu/nu mice and found that RRSP-RBD-TAT did not suppress tumor growth in this immunodeficient setting

(Fig. S16). Taken together, these two datasets indicate that dependence on IFNγ^{+} CD8^{+} T cells generalizes beyond a single model.

We added the MC-38 depletion data to Figure S20 (lines 250–251) and updated the Methods accordingly.

Reviewer #1 (Remarks to the Author):

This is a resubmission of a study examining the protein-based RAS inhibitor consisting of the RRSP fused to the RBD and then attached to various cell penetrating sequence. While the authors demonstrate that this reagent induces RAS cleavage in model systems and in some cased tumors, there remain questions regarding the interpretation of results using the RRSP-RBD-TAT reagent. The authors state that this agent enters cells and promotes RAS cleavage yet is unable to induce tumor inhibition in athymic mouse systems. This result is perplexing given that many human RAS mutant cell lines have been shown to be dependent on RAS for their tumorigenic activity in athymic nude mice. For example, MiaPaCa-2 cells are inhibited in their tumorigenic capacity using AMG510 (see Canon et al Nature 2019).

Response 1:

We thank the reviewer very much for the careful reading of our manuscript and for raising this important point. We agree that, at first glance, the observation that RRSP-RBD-TAT induces RAS cleavage but does not produce a statistically significant antitumor effect in athymic nude mice may appear paradoxical, especially in light of the robust activity of the clinical KRAS-G12C inhibitor AMG 510 in similar models (e.g., Canon et al., Nature 2019). However, for the reasons outlined below, we do not believe that our data are inconsistent with the AMG 510 literature.

First, the cellular RAS-inhibitory activity of RRSP-RBD-TAT is substantially weaker than that of AMG 510. RRSP-RBD-TAT inhibits the proliferation of cancer cells with an IC_{50} in the several-hundred-nanomolar range (Fig. 3A), whereas AMG 510 inhibits KRAS-G12C–mutant cancer cells at low-nanomolar concentrations (Canon et al., Nature 2019). Thus, although RRSP-RBD-TAT clearly induces RAS cleavage, the depth and duration of pathway inhibition achieved in vivo are likely much lower than those obtained with AMG 510. We therefore consider it plausible that the degree of RAS inhibition produced by RRSP-RBD-TAT in the xenograft setting is simply insufficient to induce a robust inhibition of tumor growth.

Second, even in the athymic xenograft model, RRSP-RBD-TAT does not appear completely inactive. As shown in our xenograft data (Fig. 3C), there is a reproducible trend toward tumor growth inhibition in RRSP-RBD-TAT–treated mice (e.g., DLD-1, SW480, and SW620), although this effect does not reach statistical significance with the current group size. We therefore think a more precise description of our findings is not that "RAS is inhibited but there is no antitumor effect," but rather that "RAS is inhibited, yet the magnitude and/or duration of inhibition in immunodeficient mice is insufficient to yield a statistically significant antitumor effect."

On this basis, the main conclusion of our work is that RRSP-RBD-TAT, although a relatively modest RAS inhibitor by itself, can elicit strong antitumor effects when it cooperates with an intact antitumor immune system. In contrast, AMG 510 is a highly potent small-molecule inhibitor and therefore shows clear efficacy even in immunodeficient xenograft models, while exhibiting further enhanced activity in immunocompetent settings. Canon et al. also demonstrated that, in immunocompetent CT-26 KRAS-G12C models, AMG 510 generates durable cures in the majority of treated mice (6/8 in Fig. 2h, Nature 2019), whereas no complete regressions are observed in an immunodeficient model (0/10 in Extended Data Fig. 4e, Nature 2019), highlighting a critical contribution of adaptive immunity to maximal efficacy.

Furthermore, examples in which RAS is almost completely degraded and inhibited yet no statistically significant antitumor effect is observed can also be seen in several immunocompetent syngeneic models. As shown in Fig. S17B, RAS is almost completely degraded in RENCA and LL/2 tumors, but, as in Fig. 3C, these models show little antitumor effect. In these models, we confirmed that intratumoral infiltration of $IFN\gamma^{+}$ $CD8^{+}$ T cells is weak (Fig. 4F). Thus, even when RAS is inhibited, RRSP-RBD-TAT does not produce a statistically significant antitumor effect if antitumor immunity is insufficient.

Taken together, we therefore consider that our findings do not contradict prior work with AMG 510 or other RAS pathway inhibitors. Rather, they support the emerging view that (i) the degree and duration of RAS inhibition and (ii) the immune context together determine the magnitude of in vivo responses to RAS-targeted agents.

Also, it is difficult to interpret Fig S17C as the RAS bands in MiaPaCa2 samples are quite faint and it is difficult to discern whether there was significant RAS cleavage.

Response 2:

We thank the reviewer for this helpful comment and apologize that Fig. S17C was not sufficiently clear. In the original figure, the contrast of the blot was adjusted to visualize the relatively strong RAS signals in HCT116 and SW480 cells, which express higher levels of RAS. As a result, the RAS bands in the MiaPaCa-2 samples appeared comparatively faint, making it difficult to assess the extent of RAS cleavage.

To address this issue, we have reprocessed the same blot and generated an additional panel in which the contrast is optimized specifically for the MiaPaCa-2 lanes. This revised image has now been incorporated into Fig. S17C. As shown in the updated panel, a lower-molecular-weight band corresponding to cleaved RAS is detectable in RRSP-RBD-TAT–treated MiaPaCa-2 samples, supporting our conclusion that RAS is indeed cleaved in this model.

SW480 cells are inhibited in tumor formation by RRSP-RBD-DTB but not with RRSP-RBD-TAT. Authors should demonstrate engagement of RAS in both tumor models. It appears that the DTP version does indeed cleave RAS in vitro in SW480 but no data shown for tumor even though there is significant tumor inhibition.

Response 3:

We thank the reviewer for this helpful suggestion. We agree that it is important to demonstrate on-target RAS engagement in the SW480 tumor model, particularly because RRSP-RBD-DTB, but not RRSP-RBD-TAT, produces clear inhibition of tumor growth in the immunodeficient xenograft setting.

We had previously examined RAS status in SW480 xenograft tumors treated with RRSP-RBD-DTB, and, in response to this comment, we now include these data in the revised manuscript. Tumors were harvested after treatment and analyzed by immunoblotting. As shown in the newly added in vivo data (Supplementary Fig. S5C), RRSP-RBD-DTB treatment leads to a clear reduction of full-length RAS and the appearance of the characteristic lower-molecular-weight RAS cleavage product, demonstrating that RRSP-RBD-DTB indeed cleaves RAS in SW480 tumors in vivo. We have also added a description of these data to the main text (line 161 in the revised manuscript).

In addition, the TAT version does not apparently result in KRAS cleavage in SW480 in vitro as shown in Fig S8 but does apparently cleave RAS in nu/nu tumors as shown in Fig. S17C. These results are inconsistent. If Fig. 17C is correct, then why is there not tumor suppression?

Response 4:

We thank the reviewer for this very insightful comment. As pointed out, RRSP-RBD-TAT shows only limited RAS cleavage in SW480 cells in vitro (Fig. S8), whereas in the nu/nu tumor setting we observe clear evidence of RAS cleavage (Fig. S17C). At first glance, these results may appear inconsistent; however, we do not believe they are contradictory for the following reasons.

First, it is not uncommon for drugs to show quantitative discrepancies between their in vitro and in vivo activities. In the case of RRSP-RBD-TAT, our data suggest that its intracellular delivery efficiency and functional activity are higher in vivo than under standard in vitro culture conditions. RRSP-RBD-TAT is internalized via a cationic cell-penetrating peptide (CPP), mainly through endocytic pathways (Fig. 1A), and factors such as cell-surface charge, composition of the extracellular milieu, and the overall level of endocytic activity are likely to differ substantially between cultured cells and tumor tissue in vivo. Although a detailed mechanistic analysis of these differences is beyond the scope of the present study, we consider these

context-dependent factors a plausible explanation for the more pronounced RAS cleavage observed in vivo, and we are therefore confident that the in vivo data shown in Fig. S17C are reliable.

The remaining question, as the reviewer rightly notes, is why detectable RAS inhibition in vivo does not translate into clear tumor suppression. As elaborated in our Response 1, even though RRSP-RBD-TAT appears to be more active in vivo than in vitro with respect to RAS cleavage, this activity is still not sufficient, on its own, to induce measurable tumor regression in the SW480 xenograft model. RRSP-RBD-TAT is a relatively weak RAS inhibitor compared with clinical small-molecule inhibitors (e.g., AMG 510) and requires cooperation with antitumor immunity to achieve robust antitumor effects. In the nu/nu model, where adaptive immunity is compromised, the level and duration of RAS inhibition achieved by RRSP-RBD-TAT are therefore not enough to translate into significant tumor growth suppression, despite detectable RAS cleavage in tumor tissues.

This discrepancy begs the question of whether their RRSP-RBD-TAT reagent is working as they propose.

Response 5:

We appreciate the reviewer's concern, and we understand that the apparent discrepancies in the SW480 and MiaPaCa-2 data may raise the question of whether RRSP-RBD-TAT is truly acting as we propose. However, we would respectfully ask the reviewer to consider the totality of the data presented in the manuscript.

First, the SW480 and MiaPaCa-2 experiments highlighted by the reviewer are provided as supplementary data. Their purpose is to broaden the characterization of RRSP-based constructs across additional models, rather than to serve as the primary basis for our mechanistic conclusions. The central claims of the manuscript—that RRSP-RBD-TAT inhibits RAS and cooperates with antitumor immunity to induce tumor regression—are supported mainly by the data in the main figures. In particular, the series of experiments summarized in Fig. 4 (analysis of the intratumoral immune microenvironment and neutralizing-antibody–based blocking experiments) provide direct and internally consistent evidence that the antitumor activity of RRSP-RBD-TAT depends on an intact antitumor immune response.

Second, as detailed in our Responses 1–4, the supplementary SW480 and MiaPaCa-2 data are, in our view, fully compatible with this proposed mechanism. RRSP-RBD-TAT has relatively weak intrinsic RAS-inhibitory activity when compared with previous RAS inhibitors that have entered clinical trials (e.g., AMG 510), and by itself does not induce tumor regression in immunodeficient models. In this context, the SW480 and MiaPaCa-2 results delineate the quantitative limits of RRSP-RBD-TAT monotherapy under conditions of limited or absent adaptive immunity, rather than indicating a failure of the reagent to engage its intended target.

Taken together, we believe that the overall dataset consistently supports our central model in which RRSP-RBD-TAT exerts modest direct RAS inhibition that, when combined with antitumor immunity, leads to robust tumor regression.

The authors have not convincingly shown the specificity for just H, K, and NRAS as raised in the prior review. The RBD targets other RAS proteins including RAP and RRAS proteins suggesting that the effects both in tumor cells and the TME including immune cells could be due in part to effects outside of the canonical H, K, and NRAS.

Response 6:

We thank the reviewer for raising this important point regarding target specificity. The main aim of our study is to develop a novel pan-RAS inhibitor and to demonstrate that its antitumor effect arises from the cooperation between RAS inhibition and IFNγ-dependent $CD8^+$ antitumor immunity. In this context, our central conclusion does not depend on whether pan-RAS inhibition is strictly confined to the canonical H-, N-, and K-RAS isoforms, or whether minor RAS-family homologs such as Rap1 or MRAS are also affected.

A comprehensive, proteome-wide characterization of all potential targets, including noncanonical RAS-related GTPases in both tumor and immune cells, would require additional technologies (e.g., unbiased proteomics or chemoproteomic profiling) that are beyond the scope of the present work.

That said, we agree that the binding profile of the RBD is relevant. The RBD used in our constructs is derived from C-Raf, and previous biochemical studies have reported that its affinity for RRAS is approximately 100-fold lower than for HRAS, KRAS, and NRAS (Nakhaeizadeh et al., PLOS ONE 2016), and that its affinity for Rap1 is also weaker, by roughly one order of magnitude (Wohlgemuth et al., J. Mol. Biol. 2005). On this basis, we expect the primary molecular targets of our RRSP-RBD fusion proteins to be the canonical RAS isoforms, which are also the major drivers of oncogenic signaling in the models we study.

However, we cannot fully exclude the possibility that cleavage of noncanonical RAS-family members such as MRAS or Rap1 may contribute to specific aspects of the immune response or to modulation of the tumor microenvironment. In response to the reviewer's comment, we have now explicitly acknowledged this possibility in the Discussion and noted that future work using more comprehensive target-profiling approaches will be required to delineate in detail the contribution of these minor RAS-family homologs (line 327 in the revised manuscript).

Minor point

Fig 3. I and J legend does not explain what the two different lanes in each sample represent. If from two tumors (assume >5 were analyzed), how many total were available and why are the others not included?

Fig. 5C. Please indicate the meaning of arrow in legend.

Response 7:

For Fig. 3I and 3J, we apologize for the lack of clarity in the legend. In each condition, the two lanes correspond to samples from two individual tumors (N = 2 mice) from a single experiment. We repeated this mouse experiment twice (total N = 4 tumors) and show a representative blot from these two independent experiments. Because this assay showed high reproducibility, we chose to perform the study with N = 4 in total for reasons of animal welfare. We have revised the figure legend to clarify these points (line 814 in the revised manuscript).

For Fig. 5C, we thank the reviewer for pointing this out. The arrow indicates the location of the subcutaneously transplanted tumor. We have now added this explanation to the figure legend (line 837 in the revised manuscript).

Reviewer #2 (Remarks to the Author):

I would like to congratulate the authors on their comprehensive experimental response to my concerns.

They may wish to consider mentioning the results obtained on Rap1a as "little to no obvious effect" and including the figure in the supplementary data.

Response 8:

We thank the reviewer very much for the positive and encouraging comment. Regarding Rap1A, the immunoblotting data we have obtained so far are preliminary, and we therefore prefer not to include these results in the main manuscript or the supplementary data at this stage.

We note that, if the manuscript is accepted and the peer-review file is made public, the present point-by-point response will be accessible to readers, so the Rap1A results will remain transparently documented even though they are not included in the main text or supplementary data.
